# rnaSPAdes: a *de novo* transcriptome assembler and its application to RNA-Seq data

**DOI:** 10.1093/gigascience/giz100

**Published:** 2019-09-03

**Authors:** Elena Bushmanova, Dmitry Antipov, Alla Lapidus, Andrey D Prjibelski

**Affiliations:** Center for Algorithmic Biotechnology, Institute of Translational Biomedicine, St. Petersburg State University, St. Petersburg, 199004, 6 linia V.O. 11d, Russia

**Keywords:** RNA-Seq, *de novo* assembly, transcriptome assembly

## Abstract

**Background:**

The possibility of generating large RNA-sequencing datasets has led to development of various reference-based and *de novo* transcriptome assemblers with their own strengths and limitations. While reference-based tools are widely used in various transcriptomic studies, their application is limited to the organisms with finished and well-annotated genomes. *De novo* transcriptome reconstruction from short reads remains an open challenging problem, which is complicated by the varying expression levels across different genes, alternative splicing, and paralogous genes.

**Results:**

Herein we describe the novel transcriptome assembler rnaSPAdes, which has been developed on top of the SPAdes genome assembler and explores computational parallels between assembly of transcriptomes and single-cell genomes. We also present quality assessment reports for rnaSPAdes assemblies, compare it with modern transcriptome assembly tools using several evaluation approaches on various RNA-sequencing datasets, and briefly highlight strong and weak points of different assemblers.

**Conclusions:**

Based on the performed comparison between different assembly methods, we infer that it is not possible to detect the absolute leader according to all quality metrics and all used datasets. However, rnaSPAdes typically outperforms other assemblers by such important property as the number of assembled genes and isoforms, and at the same time has higher accuracy statistics on average comparing to the closest competitors.

## Background

While reference-based methods for RNA-sequencing (RNA-Seq) analysis [1–6] are widely used in transcriptome studies, they are subjected to the following constraints: (i) they are not applicable when the genome is unknown, (ii) their performance deteriorates when the genome sequence or annotation is incomplete, and (iii) they may miss unusual transcripts such as fusion genes or genes with short unannotated exons. To address these constraints, *de novo* transcriptome assemblers [7–11] have emerged as a viable complement to the reference-based tools. Although *de novo* assemblers typically generate fewer complete transcripts than the reference-based methods for organisms with accurate reference sequences [12], they may provide additional insights on aberrant transcripts.

While transcriptome assembly may seem to be a simpler problem than genome assembly, RNA-Seq assemblers have to address the complications arising from highly uneven read coverage depth caused by variations in gene expression levels. However, this is the same challenge that we have addressed while developing the SPAdes assembler [13,14], which originally aimed at single-cell sequencing. Similarly to RNA-Seq, the multiple displacement amplification (MDA) technique [15], used for genome amplification of single bacterial cells, results in a highly uneven read coverage. In view of similarities between RNA-Seq and single-cell genome assemblies, we decided to test SPAdes without any modifications on transcriptomic data. Even though SPAdes is a genome assembler and was not optimized for RNA-Seq data, in some cases it generated decent assemblies of quality comparable to the state-of-the-art transcriptome assemblers.

To perform the benchmarking we have used the rnaQUAST tool [16], which was designed for quality evaluation of *de novo* assemblies with the support of a reference genome and its gene database. For the comparison, we selected a few representative metrics such as (i) total number of assembled transcripts (contigs), (ii) reference gene database coverage, (iii) number of 50%/95%-assembled genes/isoforms, (iv) number of misassemblies, and (v) duplication ratio. A detailed description of these metrics can be found in the Supplementary material (section S1).

Table 1 compares the performance of different assembly tools on a publicly available mouse RNA-Seq dataset. All transcriptome assemblers were launched with default parameters; SPAdes was run in single-cell mode owing to the uneven coverage depth of RNA-Seq data. Table 1 shows that SPAdes generated more 50%/95%-assembled genes than any other tool and yielded comparable gene database coverage. At the same time, SPAdes produced a rather high number of misassembled transcripts, which can be explained by the fact that algorithms for genome assembly tend to assemble longer contigs and may incorrectly join sequences corresponding to different genes when working with RNA-Seq data. In addition, SPAdes generated the same number of 95%-assembled genes and isoforms, which emphasizes the lack of an isoform detection step.

**Table 1:** Benchmarking of BinPacker, Bridger, IDBA-tran, RNA-Bloom, SOAPdenovo-Trans, SPAdes, Trans-ABySS, and Trinity on mouse RNA-Seq dataset (accession No. SRX648736)

Parameter	BinPacker	Bridger	IDBA	Bloom	SOAP	SPAdes	ABySS	Trinity
Transcripts	27,234	42,029	38,313	46,440	31,878	42,949	36,488	47,746
Misassemblies	947	923	387	732	37*	497	194	459
Duplication ratio	1.12	1.09	1.00*	1.33	1.00*	1.00*	1.09	1.15
Database coverage, %	14.4	16.3	16.9	13.8	15.1	17.7	16.2	18.2*
50%-assembled genes	6,005	6,090	6,558	4,859	6,241	6,890*	6,321	6,633
95%-assembled genes	1,917	1,909	1,602	1,256	1,653	2,450*	1,798	2,272
50%-assembled isoforms	6,360	6,451	6,790	5,591	6,376	7,053	6,931	7,386*
95%-assembled isoforms	1,992	1,982	1,602	1,346	1,655	2,450*	1,850	2,406

The annotated transcriptome of *Mus musculus* GRCm38.75 consists of 38,924 genes and 94,545 isoforms. All contigs shorter than 200 bp were filtered out prior to the analysis. The best values for each metric are indicated with asterisk.

Benchmarking on other datasets also showed that SPAdes successfully deals with non-uniform coverage depth and produces a relatively high number of 50%/95%-assembled genes in most cases. However, it also confirmed the problem of a large amount of erroneous transcripts as well as relatively few fully reconstructed alternative isoforms in SPAdes assemblies. On the basis of the obtained statistics we decided to adapt current SPAdes algorithms for RNA-Seq data with the goal of improving the quality of generated assemblies and develop a new transcriptomic assembler called rnaSPAdes. In this article we describe major pipeline modifications as well as several algorithmic improvements introduced in rnaSPAdes that allow it to avoid misassemblies and obtain sequences of alternatively spliced isoforms.

To perform sufficient benchmarking of rnaSPAdes and the other aforementioned transcriptome assemblers, we assembled several simulated and publicly available real RNA-Seq datasets from the organisms with various splicing complexity. For the generated assemblies we present quality assessment reports obtained with different *de novo* and reference-based evaluation approaches. In addition, based on these results we discuss strengths and disadvantages of various assembly tools and provide insights on their performance.

## Data Description

To compare the performance of rnaSPAdes with that of the state-of-the-art transcriptome assemblers we selected 2 simulated and 6 real publicly available RNA-Seq datasets (Table 2) with different (i) read length, (ii) library size, (iii) strand specificity, and (iv) organism splicing complexity. Simulated data were generated using the RSEM simulator [1] based on the real human and mouse datasets used in this study (the exact commands are provided in Supplementary material section S3).

**Table 2:** RNA-Seq datasets selected for comparison of different assembly tools

Dataset name	Organism	Tissue	No. of read pairs (millions)	Strand-specific	Read length (bp)	Insert size (bp)	Accession No.
Human	*Homo sapiens*	Prostate cancer cells	30	No	150	344	SRR5133163
Human large	*H. sapiens*	Blood	125	No	100	176	SRR1957703, SRR1957706
Mouse	*Mus musculus*	Pancreatic islets	11	No	101	173	SRX648736
Worm	*Caenorhabditis elegans*	NA	45	No	90	186	SRR1560107
Corn SS	*Zea mays*	Endosperm	35	RF	100	242	SRR1588569
Arabidopsis SS	*Arabidopsis thaliana*	NA	118	RF	130	245	SRR5344669, SRR5344670
Human simulated	*H. sapiens*	NA	30	No	150	340	NA
Mouse simulated	*M. musculus*	NA	11	No	101	170	NA

All datasets contain paired-end Illumina reads. RF stands for reverse-forward strand-specific data, i.e. when the first read in pair has the opposite strand from the gene strand. NA stands for not available.

All downloaded public datasets were analyzed using FastQC [17]. The reports showed that no dataset contains adaptors or overrepresented sequences. The Human large dataset was quality-trimmed using Trimmomatic [18] owing to a decrease in quality towards the reads' ends. All other datasets were assembled without additional preprocessing. Although the 8 datasets used in this article may not represent all kinds of transcriptomic data, they are sufficient for comparing different assembly tools and detecting their strengths and disadvantages.

## Analyses

Selected datasets were assembled with BinPacker [19], Bridger [20], IDBA-tran [10], RNA-Bloom [21], SOAPdenovo-Trans [11], Trans-ABySS [7], Trinity [8], and rnaSPAdes using default parameters, and SPAdes [13] in single-cell mode. While rnaSPAdes automatically calculates *k*-mer sizes based on the read length (see Methods for details), other assemblers have fixed default *k*-values. Indeed, varying *k*-value may affect the assembly in both positive and negative ways. However, because detecting the best *k*-mer sizes for all third-party assemblers requires additional large-scale analysis and is outside the scope of this work, it remains unclear how to properly select *k* for other tools. Thus, we decided to stick to default *k*-values, which were used in the original manuscripts or suggested in the user manuals by their developers and therefore are likely to be used by users.

For a fair comparison the same minimal contig length cut-off was used for all tools (200 bp). For assemblers that have no such option, sequences shorter than 200 bp were filtered out manually. To evaluate the resulting assemblies we used rnaQUAST [16], Transrate [22], BUSCO [23], and DETONATE [24]. From each quality report we selected a few representative metrics that would allow us to perform a complete comparison of different assemblers. To make the results reproducible, we also specify the software versions and command lines used in this study in the Supplementary material (sections S2 and S3).

In addition to statistics provided by different tools, we decided to compute the fraction of 95%-assembled genes relative to the number of genes detected by a reference-based method. For this purpose we used genes assembled by kallisto [25] that have nucleotide coverage >5. Coverage values were computed using estimated fragment counts. While it remains unclear how to select a proper coverage threshold for this experiment, the number of genes/isoforms with coverage >5 seemed to be the best upper bound estimate for most of the datasets (see Supplementary Table S15 for details). Using the fraction of assembled genes instead of raw numbers allows the data to be conveniently visualized in the same plot, average values to be computed across all datasets, and, at the same time, the relative performance of *de novo* assemblers to the reference-based tool to be estimated.

### Evaluating assemblers on simulated data

To simulate an RNA-Seq dataset we used RSEM simulator [1], which allows reads to be generated on the basis of the real RNA-Seq data. For this purpose we selected the Human and Mouse datasets (Table 2). Table 3 presents a short quality assessment report for Human simulated data. Complete evaluation reports for both simulated datasets are presented in the Supplementary material (Tables S1 and S2).

**Table 3:** Benchmarking of BinPacker, Bridger, IDBA-tran, RNA-Bloom, rnaSPAdes, SOAPdenovo-Trans, SPAdes, Trans-ABySS, and Trinity on Human simulated RNA-Seq dataset

Parameter	BinPacker	Bridger	IDBA	Bloom	rnaSPAdes	SOAP	SPAdes	ABySS	Trinity
Transcripts	76,736	52,151	58,466	65,968	37,730	35,096	42,264	67,511	62,831
Misassemblies	7,919	3,512	174	358	309	198	443	126*	1,554
Duplication ratio	2.19	1.38	1.01	1.93	1.26	1.08	1.00*	1.24	1.74
Database coverage, %	20.9	18.5	21.4	24.6*	23.2	19.4	20.5	23.1	24.4
50%-assembled genes	11,828	11,476	13,175	12,869	14,075*	12,610	13,569	12,740	13,289
95%-assembled genes	7,320	6,417	2,729	8,910	10,934*	7,685	8,526	7,225	9,049
50%-assembled isoforms	17,415	15,423	18,181	21,035*	19,531	15,638	16,437	19,250	20,965
95%-assembled isoforms	9,091	7,298	2,744	12,108	13,387*	8,151	8,638	7,662	12,301

The annotated transcriptome of *H. sapiens* GRCh37.p13 consists of 57,820 genes and 196,520 isoforms. All contigs shorter than 200 bp were filtered out prior to the analysis. The best values for each metric are indicated with asterisk.

Table 3 shows that rnaSPAdes produced the most 95%-assembled genes and isoforms, with Trinity and RNA-Bloom being the closest competitors. Trinity and RNA-Bloom also had the highest gene database coverage, while rnaSPAdes and Trans-ABySS are just slightly behind (1.5% difference at most). However, both Trinity and RNA-Bloom seemed to produce a lot of excessive sequences, resulting in high duplication ratios (1.74 and 1.93, respectively), and Trinity also seemed to be somewhat inaccurate in terms of misassembled sequences (5 times more than rnaSPAdes). Among the tools with a high number of assembled genes and isoforms, Trans-ABySS and SOAPdenovo-Trans are the most accurate (126 and 198 misassemblies, respectively); rnaSPAdes and RNA-Bloom follow with 309 and 358 misassembled contigs, respectively. Although IDBA also generated an accurate assembly (174 misassemblies), it seemed to be fragmented (few 95%-assembled genes and isoforms). Although both BinPacker and Bridger produced a comparable amount of assembled genes and isoforms, they had the most misassemblies (>3,500). BinPacker also had the highest duplication ratio (2.19).

**Figure 1:**
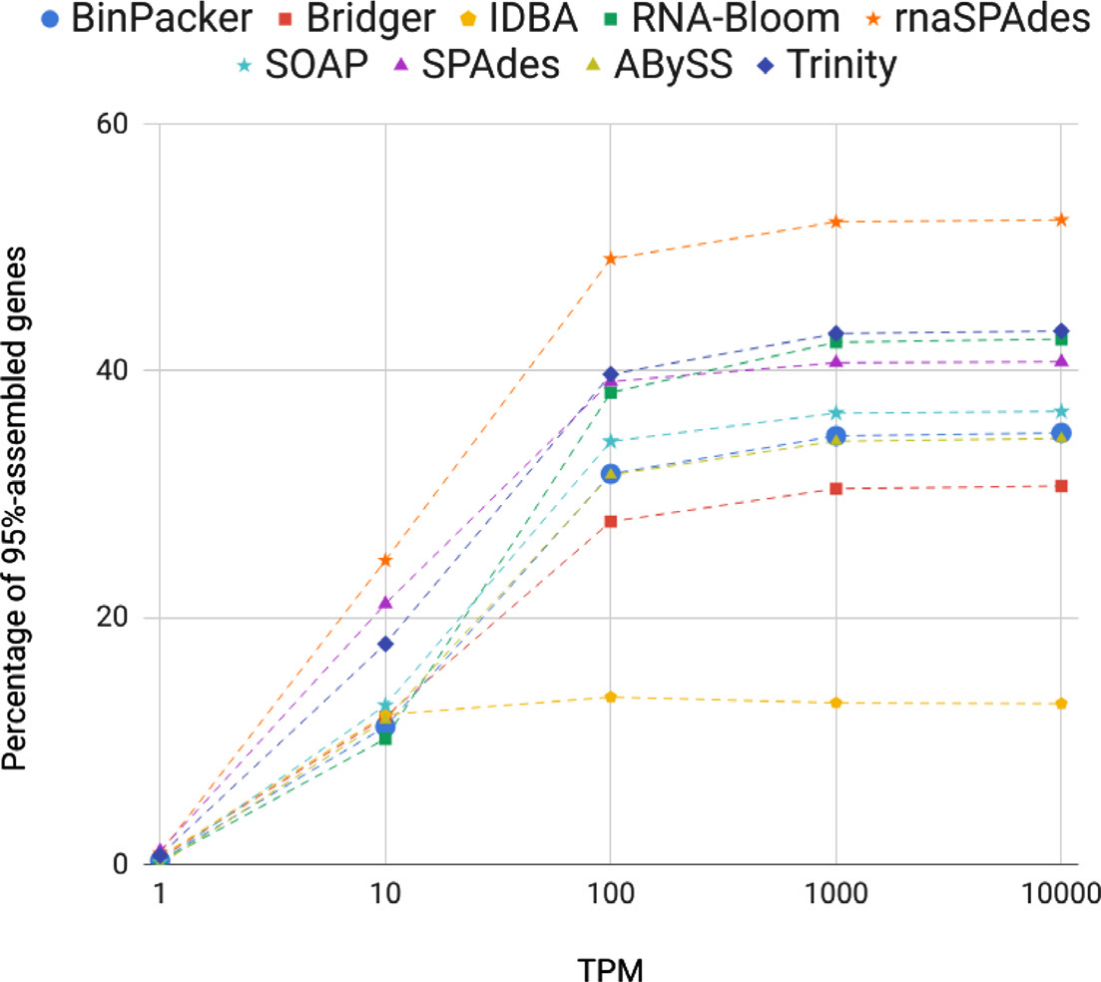
Cumulative plot showing how fraction of 95%-assembled genes in each assembly of human simulated dataset depends on the gene coverage by reads in TPM (transcripts per kilobase million) reported by RSEM simulator.

Because the RSEM simulator provides read count for each particular gene, we also computed the number of assembled genes reported by rnaQUAST depending on their read coverage (Fig. 1). The figure demonstrates that rnaSPAdes, SPAdes, and Trinity outperformed other tools on low-abundant transcripts, with rnaSPAdes reaching the highest fraction of total 95%-assembled genes (52.2%).

### Evaluating assemblers on real RNA-Seq data

**Figure 2:**
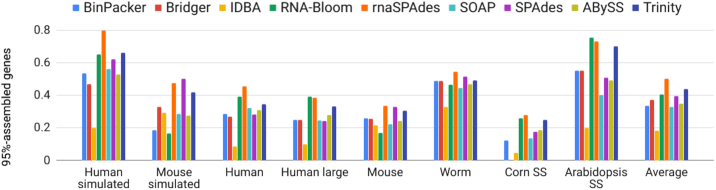
The fraction of 95%-assembled genes presented as bar plots for all generated assemblies. The values are computed relative to the number of genes reported by kallisto [25] that have per-base coverage depth >5. The last columns show average values over all datasets. Note that Bridger failed to assemble the corn dataset.

For comparison on real RNA-Seq reads we selected 4 non-stranded and 2 strand-specific datasets (Table 2). The summary for human assemblies is presented in Table 4, while complete reports for all data are presented in the Supplementary material (Tables S3–S8, respectively). In addition, we added BUSCO reports (Supplementary Fig. S2) and presented various metrics as bar plots (Fig. 2, Supplementary Figs S3–S5).

**Table 4:** Benchmarking of BinPacker, Bridger, IDBA-tran, RNA-Bloom, rnaSPAdes, SOAPdenovo-Trans, SPAdes, Trans-ABySS, and Trinity on real human dataset

Parameter	BinPacker	Bridger	IDBA	Bloom	rnaSPAdes	SOAP	SPAdes	ABySS	Trinity
Transcripts	144,598	191,459	173,330	239,912	167,710	140,769	223,917	190,798	234,074
Misassemblies	9,898	7,487	1,015	1,643	2,111	450*	3,190	916	5,183
Duplication ratio	2.03	1.61	1.02	2.75	1.36	1.12	1.01*	1.25	2.00
Database coverage, %	17.2	16.6	19.6	24.8*	21.3	18.5	18.4	22.5	24.2
50%-assembled genes	10,763	10,534	11,712	12,779	13,377*	12,154	12,395	12,621	12,902
95%-assembled genes	4,457	4,226	1,334	6,121	7,094*	5,051	4,427	4,844	5,398
50%-assembled isoforms	15,133	14,032	16,260	22,547*	18,619	15,302	15,533	19,817	21,876
95%-assembled isoforms	5,080	4,680	1,338	7,976	8,026*	5,259	4,455	5,046	6,753

The annotated transcriptome of *H. sapiens* GRCh38.82 consists of 57,820 genes and 196,520 isoforms. All contigs shorter than 200 bp were filtered out prior to the analysis. The best values for each metric are indicated with asterisk.

Table 4 indicates that while all assemblies had >10,000 genes that were 50%-assembled, the amount of 95%-assembled genes significantly differs. RnaSPAdes, RNA-Bloom, and Trinity were the best according to 95%-assembled genes and isoforms. Among these 3 assemblers, rnaSPAdes dominates with 16% and 31% more 95%-assembled genes than RNA-Bloom and Trinity, respectively. Although both RNA-Bloom and Trinity had the highest database coverage, they also have a very high duplication ratio (≥2). In addition, Trinity (along with BinPacker and Bridger) generated a significant amount of misassemblies (>5,000). SOAPdenovo-Trans and Trans-ABySS produced accurate assemblies according to these parameters but generated 2,043 and 2,250 fewer 95%-assembled genes than rnaSPAdes. IDBA-trans also had a rather small number of misassembled contigs (1,015) but output a very fragmented assembly with the fewest 95%-assembled genes/isoforms.

Fig. 2 demonstrates the fraction of 95%-assembled genes in all generated assemblies and mean values for each assembler across all datasets. RnaSPAdes, Trinity, and RNA-Bloom showed stable performance across different datasets and had the highest fraction of 95%-assembled genes on average (0.500, 0.438, and 0.406, respectively). While genomic SPAdes also had a high average value (0.397), it was mostly achieved by decent performance on simulated data. Fig. 2 shows that the fraction of 95%-assembled genes for simulated datasets was typically higher than the respective values for real data, most likely due to the absence of sequencing artifacts. Additionally, *de novo* assemblies of complex organisms, such as *H. sapiens* and *M. musculus*, tended to have lower fractions of assembled genes in comparison to *C. elegans* and *A. thaliana*. For example, the human large dataset had smaller values than the ones for worm assemblies, although the latter had almost 3× lower coverage.

### Computational performance

To compare selected assemblers in terms of computational performance, we measured their running time and RAM consumption on the 2 largest datasets using system utilities rather than log files. As Table 5 indicates, SOAPdenovo-Trans was ≥3 times faster than any other assembler and had one of the lowest memory requirements (<30 GB for both datasets). Trans-ABySS and rnaSPAdes had comparable performance, with rnaSPAdes being slightly faster and more greedy regarding RAM consumption. Other assemblers typically took longer (≥2 times longer than rnaSPAdes in most cases) and required more memory. Among all tools, BinPacker, Bridger, and Trinity had the highest peak RAM usage, e.g., >100 GB with the Arabidopsis dataset.

**Table 5:** Running time and peak RAM usage for BinPacker, Bridger, IDBA-tran, RNA-Bloom, rnaSPAdes, SOAPdenovo-Trans, SPAdes, Trans-ABySS, and Trinity on the Human large and Arabidopsis SS datasets (125 and 118 million read-pairs, respectively)

Assembler	Human large		Arabidopsis SS	
	Time	RAM (GB)	Time	RAM (GB)
BinPacker	46 h 59 m	91	88 h 25 m	131
Bridger	65 h 54 m	88	49 h 58 m	126
IDBA	9 h 35 m	35	26 h 24 m	42
Bloom	37 h 52 m	38	34 h 42 m	40
rnaSPAdes	5 h 4 m	32	7 h 24 m	40
SOAP	1 h 21 m	28	1 h 58 m	20
SPAdes	11 h 39 m	39	14 h 58 m	52
ABySS	6 h 49 m	25	8 h 9 m	35
Trinity	18 h 8 m	50	8 h 30 m	123

All assemblers were launched in 16 threads on a server with 128 GB of RAM and 56 Intel Xeon 2.0 GHz cores. BinPacker, which has no options for setting the number of threads, was launched with default parameters.

## Discussion

Quality reports provided in this article (Tables 3 and 4) and the Supplementary material (Tables S1–S8, Figs S1–S5) contain a large variety of metrics that reflect completely different assembly properties, the importance of which may vary depending on the further analysis and the entire pipeline being used. We believe that one of the key features of the *de novo* transcriptome assembler is the ability to correctly capture the entire transcript into a single contig (e.g., reflected by the number of 95%-assembled genes/isoforms, contig recall). On the other hand, such metrics as gene database coverage, number of covered reference proteins, and nucleotide recall do not reflect this significant property because they account *all* contigs mapped to a specific gene or protein and do not include assembly contiguity. For example, high database coverage or nucleotide recall can be achieved by a very fragmented assembly (or even raw reads), which, indeed, does not suit well for further reference-free analysis.

Below we attempt to summarize these results and highlight strong and weak points of different assemblers.

### Comparison between SPAdes and rnaSPAdes

In comparison with the original version of SPAdes, rnaSPAdes dominates by the majority of metrics. More precisely, it has significantly better assembly completeness metrics: 26% higher average fraction of 95%-assembled genes, 18% larger database coverage, 30% higher contig recall reported by REF-EVAL, and 18% more detected BUSCOs. It also shows 18% higher contig precision on average, better reference coverage metrics reported by Transrate (50%/95%-covered reference proteins, reference coverage) and typically fewer misassemblies (except for Corn SS and Human large datasets). Due to an aggressive overlap removal stage, SPAdes always has a smaller mean duplication ratio (2% vs 32% for rnaSPAdes), fewer duplicated BUSCOs (1% vs 16% on average), percentage of uncovered bases (2% vs 19%), and higher nucleotide precision (0.66 vs 0.56).

The simulated Mouse dataset is the only one where the original SPAdes generated more assembled genes and isoforms than rnaSPAdes. Detailed investigation showed that the key reasons are the low coverage of these data (11 million reads) and their artificial nature (rnaSPAdes assembles more genes on real mouse data). By using small *k* = 21 during the first iteration SPAdes manages to assemble extremely low-covered genes, where overlaps between reads are short. Pitfalls of using small *k*-mer sizes in transcriptome assembly are discussed in the Methods section.

Finally, due to the exclusion of the BayesHammer error correction module [26] and using only 2 *k*-mer sizes, the rnaSPAdes pipeline seems to be about twice as fast and consumes less RAM than usual SPAdes use.

### Assembly completeness

In comparison with other assemblers, rnaSPAdes shows the highest fraction of 95%-assembled genes and isoforms (0.50 and 0.32, respectively). Trinity (0.44 and 0.30) and RNA-Bloom (0.41 and 0.28) are ranked second and the third according to these metrics (Supplementary Fig. S3). These numbers correlate with the percentage of detected BUSCOs, for which rnaSPAdes also has the best average value across all datasets (74%), followed by Trinity (72%), Trans-ABySS (71%), and RNA-Bloom (68%).

The same assemblers typically form the top 4 according to various coverage metrics, such as database coverage provided by rnaQUAST, reference coverage, number of 50%/95%-covered reference proteins, and number of reference sequences with CRBB (Conditional Reciprocal Best BLAST [27) hits reported by Transrate (Supplementary Figs S3 and S5). For example, according to mean gene database coverage computed by rnaQUAST, Trinity has the highest value of 30.2%, with other assemblies having somewhat lower values: 29.6% for RNA-Bloom, 28.7% for rnaSPAdes, and 24.2% for Trans-ABySS. Exactly the same ranking is defined by Transrate reference coverage: Trinity (27.8%), RNA-Bloom (26.9%), rnaSPAdes (24.4%), and Trans-ABySS (23.4%). Other assemblers typically show smaller values for completeness-related metrics, generating fragmented assemblies, like IDBA-tran, or having lower database coverage, e.g., BinPacker.

Nucleotide and contig recall metrics reported by Detonate generally support the aforementioned conclusions (Supplementary Fig. S4). Thus, Trinity and rnaSPAdes have the best average nucleotide recall values (0.86 and 0.84, respectively). The maximal mean contig recall, however, is reported for RNA-Bloom (0.097), followed by Trinity (0.089), Trans-ABySS (0.087), and rnaSPAdes (0.079). To compute contig metrics Detonate keeps only the most reliable alignments with mapped fraction >99% (for both assembled and reference sequence). In contrast, rnaQUAST assigns contigs to known genes/isoforms and then counts those that have at least X% covered by a single assembled contig. However, no cutoff is applied for mapped fraction of the assembled sequences in rnaQUAST. This difference between algorithms might explain the absence of perfect correlation between contig recall and number of 95%-assembled isoforms.

### Assembly correctness

According to the number of misassembled contigs, the most accurate contigs are typically produced by SOAPdenovo-Trans, Trans-ABySS, and IDBA-tran (see Supplementary Fig. S3d). Among these 3, IDBA-tran, however, produces the most fragmented assemblies with the lowest average fraction of 95%-assembled genes equal to 0.18. Supplementary Fig. S3d also suggests that the highest numbers of misassemblies often belong to BinPacker, Bridger, RNA-Bloom, and Trinity.

IDBA-tran, usual SPAdes, and SOAPdenovo-Trans tend to provide assemblies with the fewest duplicated sequences, which is confirmed by rnaQUAST duplication ratio (average values are 1.02, 1.02, and 1.07, respectively), percentage of duplicated BUSCOs (0.8%, 1.0%, and 4.7%), fraction of uncovered bases reported by Transrate (0.018, 0.019, and 0.076), and Detonate’s nucleotide precision (0.68, 0.66, and 0.66). The highest contig precision equal to 0.133, however, belongs to rnaSPAdes, followed by 0.129 for SOAPdenovo-Trans. The most duplicated assemblies according to these metrics are produced by RNA-Bloom, Trinity, and BinPacker. In comparison with other assemblers, they have a significantly higher mean duplication ratio (2.5, 1.77, and 1.71, respectively) and fraction of duplicated BUSCOs (40.6%, 31.4%, and 29.7%), as well as lowest average nucleotide precision (0.37, 0.46, and 0.46). As for rnaSPAdes, according to duplication metrics and misassemblies, it neither fails nor dominates, showing a moderate average duplication ratio of 1.32 and fraction of duplicated BUSCOs equal to 16.7%.

Indeed, besides completeness-related metrics, such as number of assembled genes and isoforms, metrics discussed above should also be considered during transcriptome quality evaluation because erroneous and duplicated sequences may negatively affect further transcriptome analysis, such as gene annotation.

### Read-based scores

According to the read-based scores reported by Transrate and Detonate RSEM EVAL, which represent how well the assembly corresponds to the initial reads, rnaSPAdes also shows good results. Regarding the average Transrate contig score, conventional SPAdes has the highest average score equal to 0.31, followed by IDBA-trans and SOAPdenovo-Trans both having 0.17, and rnaSPAdes with 0.16. As for the Detonate score, rnaSPAdes has the best average (−3.45 · 10^9^), with RNA-Bloom (−3.46 · 10^9^) and Trinity (−3.84 · 10^9^) slightly behind. RNA-Bloom and Trinity, however, have the lowest Transrate average scores among all tools (0.026 and 0.084, respectively). Vice versa, SPAdes, IDBA, and SOAPdenovo-Trans, which are the top 3 assemblers according to mean Transrate score, have the lowest 3 RSEM EVAL scores. Based on the complete quality reports presented in the Supplementary material, it appears that Transrate score mostly correlates with correctness-related metrics and is negatively affected by the presence of duplicated sequences, which explains the highest average score for standard SPAdes. In contrast, RSEM-EVAL score seems to correlate with assembly completeness metrics.

### Conclusion

Although every transcriptome assembler presented in this study has its own benefits and drawbacks, the trade-off between assembly completeness and correctness can be significantly shifted by modifying the algorithms’ parameters. For example, various thresholds for transcript filtration in rnaSPAdes (Table S14 in the Supplementary material) result in assemblies with different properties. Also, varying *k*-mer size or incorporating iterative de Bruijn graph construction in rnaSPAdes may significantly affect the assembly quality (Tables S9–S11 in the Supplementary material). Thus, the parameters of the assembly algorithms can be varied in order to achieve the desired completeness or correctness characteristics and make the method dominant in a certain group of metrics.

While the developed algorithm, rnaSPAdes, typically shows stable results across analyzed RNA-Seq datasets and often allows the capture of more genes and isoforms than any other tool, there is no clear winner according to all metrics. Thus, the selection of the assembler may be varied depending on the goals of the particular research project and the sample preparation protocols being used, as well as secondary parameters, such as usability and computational performance. Even with the aid of specially developed tools, such as Transrate, DETONATE, BUSCO, and rnaQUAST, the choice of a suitable assembly tool remains a non-trivial problem and may require additional benchmarks in each particular case.

## Potential implications

Although the developed approach was initially designed for RNA-Seq data obtained from a single organism, it can potentially be applied for metatranscriptome assembly of samples collected from bacterial communities. Indeed, metatranscriptome assembly does not require reconstructing complex alternatively spliced isoforms but implies other computational challenges, such as repetitive patterns in different genes (including homologous genes from various strains) and extreme differences in messenger RNA (mRNA) quantities [28,29], which are caused by both varying expression levels and abundances of different species. Improving the assembly algorithms, as well as designing an appropriate pipeline for quality evaluation of metatranscriptomic assemblies, would be a way to build on this work.

Recently emerged long-read protocols for mRNA sequencing allow the capture of full-length transcripts without the assembly [30]. However, the high error rate of Oxford Nanopore and PacBio sequencers prevents using output reads directly as complete transcripts. Typically, mapping to the reference genome, additional error correction by short accurate Illumina reads, or consensus construction is performed to obtain and further analyze high-quality sequences [31–35]. Combining rnaSPAdes with the previously developed hybridSPAdes approach for joint assembly of short and long reads [36] may result in a viable alternative to the existing methods for processing long error-prone RNA reads.

In addition, benchmarking reports presented in this work can be used by researchers for selecting the appropriate assembly method that meets their specific criteria and for better understanding of transcriptome assembly quality evaluation, such as, for example, correlation of different metrics.

## Methods

Most of the modern *de novo* genome assembly algorithms for short reads rely on the concept of the de Bruijn graph [37]. While the initial study proposed looking for an Eulerian path traversing the de Bruijn graph in order to reconstruct genomic sequences, it seemed to be rather impractical owing to the presence of complex genomic repeats and sequencing artifacts, such as errors and coverage gaps. Instead, genome assemblers implement various heuristic approaches, most of which are based on coverage depth, graph topology, and the fact that the genome corresponds to 1 or more long paths traversing the graph [14,38]. Indeed, the latter observation is not correct for the case of transcriptome assembly, in which RNA sequences correspond to numerous shorter paths in the graph. Thus, to enable high-quality assemblies from RNA-Seq data the majority of procedures in the SPAdes pipeline have to undergo major alterations.

The SPAdes genome assembler consists of the following major steps: (i) construction of the condensed de Bruijn graph; (ii) graph simplification, which implies removing chimeric and erroneous edges; (iii) mapping read pairs to the assembly graph; and (iv) repeat resolution and scaffolding using aligned paired reads with the exSPAnder algorithm [39,40]. While graph construction and mapping paired reads do not depend on the dataset type and require no change for RNA-Seq data, graph simplification and repeat resolution procedures strongly rely on the properties of genomic sequences and thus require significant modifications and novel functionality for *de novo* transcriptome assembly. Below we describe the key changes introduced in rnaSPAdes.

### Simplification of the de Bruijn graph in rnaSPAdes

During the graph simplification stage erroneous edges are removed from the de Bruijn graph based on various criteria in order to obtain a clean graph containing only correct sequences (referred to hereafter as an "assembly graph"). In the SPAdes pipeline the simplification process includes multiple various procedures that can be classified into 3 types: (i) trimming "tips" (dead-end or dead-start edges), (ii) collapsing "bulges" (alternative paths), and (iii) removing *erroneous connections* (chimeric and other false edges). In this section we present alterations introduced in the rnaSPAdes simplification pipeline. We also provide comparison between initial and improved simplification procedures on several RNA-Seq datasets in the Supplementary material (Table S10).

#### Trimming tips

In the de Bruijn graph constructed from DNA reads the major fraction of tips (edges starting or ending at a vertex without other adjacent edges) typically correspond to sequencing errors and thus have to be removed. Because only a few tips are correct and either represent chromosome ends or are formed by coverage gaps, the existing genome assemblers implement rather aggressive tip-clipping procedures [13, 38], assuming that coverage gaps appear rather rarely. However, in the de Bruijn graph built from RNA-Seq data a significant amount of tips correspond to transcripts’ ends and thus have to be preserved. To keep the correct tips and obtain full-length transcripts, rnaSPAdes uses lower coverage and length thresholds for the tip-trimming procedure than SPAdes (see details below).

**Figure 3:**
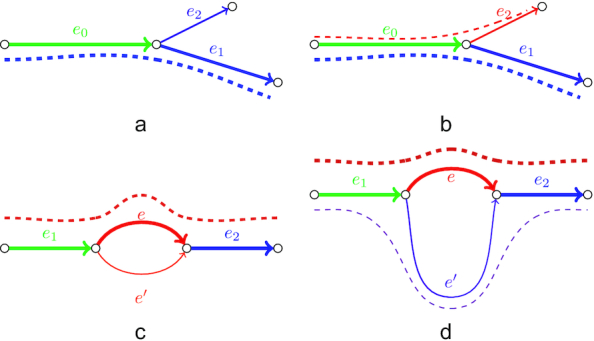
Examples of tips and bulges in the condensed de Bruijn graph. Edges with similar colors have similar sequences; line width represents the coverage depth. (a) Correct transcript (blue dashed line) traverses through edges *e*_0_ and *e*_1_. Edge *e*_2_ originates from the reads with the same sequencing error and thus has coverage depth high enough not to be trimmed. However, because the sequence of edge *e*_2_ is very similar to the sequence of the alternative edge *e*_1_ (detected by alignment), *e*_2_ is eventually removed as erroneous. (b) In this case both paths (*e*_0_, *e*_1_) and (*e*_0_, *e*_2_) correspond to correct isoforms (blue and red dashed lines). Because the sequences of *e*_1_ and *e*_2_ are likely to be different, neither of the correct tips is removed. (c) Correct sequence (red dashed line) traverses through edges *e*_1_, *e*, and *e*_2_. Edge *e*′ originates from reads containing sequencing errors and thus has sequence similar to *e* but significantly lower coverage. (d) Both paths (*e*_1_, *e, e*_2_) and (*e*_1_, *e*′, *e*_2_) correspond to different isoforms of the same gene (red and purple dashed lines); edges *e* and *e*′ typically have different length, coverage depth, and sequence.

In some cases, tips originate from sequencing errors in multiple reads from highly expressed isoforms and thus may have coverage above the threshold. While genome assemblers may also exploit a relative coverage cutoff to remove such tips, in transcriptome assembly this approach may result in trimming correct tips corresponding to the ends of low-expressed isoforms. However, erroneous tips typically have a small difference from the correct sequence without errors (e.g., 1–2 mismatches). To address this issue, we align tips to the alternative (correct) edges of the graph (Fig. 3a) and trim them if the identity exceeds a certain threshold (a similar procedure is implemented in truSPAdes, which was designed for True Synthetic Long Reads assembly [41]). In the case where 2 tips correspond to the starts/ends of alternatively spliced isoforms, it is highly unlikely for them to have similar nucleotide sequences (Fig. 3b). Such tips are preserved during the graph simplification procedure, thus allowing the restoration of isoforms that differ only by starting or terminating exons.

Another characteristic of RNA-Seq datasets is the large number of low-complexity regions that originate from poly-A tails resulting from polyadenylation at the ends of mRNAs. To avoid chimeric connections and non-informative sequences, we also remove low-complexity edges from the de Bruijn graph (see exact criterion below).

Below we summarize all conditions used in the tip-clipping procedure, parameters for which were optimized on the basis of our analysis of various RNA-Seq datasets. We define *l_T_* as the length of the tip that is being analyzed and *c_T_* as its mean *k*-mer coverage, and *c_A_* as the *k*-mer coverage of the alternative edge (which is presumably correct). A tip is removed if any of the following conditions is true:

- *l* < 2 · *k* and *c_T_* ≤ 1 (short tips with very low coverage);
- *l* < 4 · *k, c_T_* < *c_A_*/2, and the Hamming distance between the tip and the alternative edge ≤3 (the tip containing sequencing errors);
- the tip contains >80% of A/T nucleotides (low-complexity tip).

#### Collapsing bulges

A simple bulge (2 edges sharing starting and terminal vertices) in the de Burijn graph may correspond to 1 of the following events: (i) a sequencing error, (ii) a heterozygous mutation or another allele difference, or (iii) an alternative splicing event (typical for transcriptomic data). The first 2 cases are characterized by the bulge edges having similar lengths and sequences. However, edges formed by sequencing errors are typically short and have significantly different coverage depth because it is unlikely for the same error to occur numerous times at the same position (Fig. 3c). Vice versa, in the case of allele difference bulge edges usually have similar coverage. Thus, genome assembly algorithms for bulge removal typically rely on the coverage depth [13,38]. Because the most typical difference between 2 alternatively spliced isoforms of the same gene is the inclusion/exclusion of an exon (usually short), edges of the bulge originating from these isoforms have different lengths (Fig. 3d). At the same time, because the expression levels may vary for such isoforms, the coverage depth may significantly differ. To avoid missing alternatively spliced isoforms in the assembly, rnaSPAdes does not use any coverage threshold for bulge removal and collapses only bulges consisting of edges with similar lengths (<10% difference in length).

#### Removing chimeric connections

While undetected tips and bulges formed by sequencing errors result in mismatches and indels in the assembled contigs, chimeric reads (typically corresponding to a concatenation of sequences from distant regions of the original molecules) may trigger more serious errors, such as incorrect junctions in the resulting contigs (often referred to as misassemblies). In conventional genome assembly chimeric edges usually have low coverage and thus can be easily identified [38]. Single-cell datasets, however, feature multiple low-covered genomic regions and an elevated number of chimeric reads, which result in numerous erroneous connections having higher coverage depth than correct genomic edges. Similarly, because true edges representing low-expressed isoforms in the transcriptome assembly also have relatively low coverage depth, cleaning the graph using a coverage threshold will result in multiple missing transcripts in the assembly.

To detect chimeric connections in single-cell assemblies SPAdes implements various algorithms, which mostly rely on the assumption that each chromosome corresponds to a long contiguous path traversing through the de Bruijn graph [14]. Because this assumption does not hold for transcriptomes consisting of thousands isoforms, we had to disable most procedures for chimeric edge detection in SPAdes and implement a new erroneous edge removal algorithm that addresses the specifics of chimeric reads in RNA-Seq datasets.

**Figure 4:**
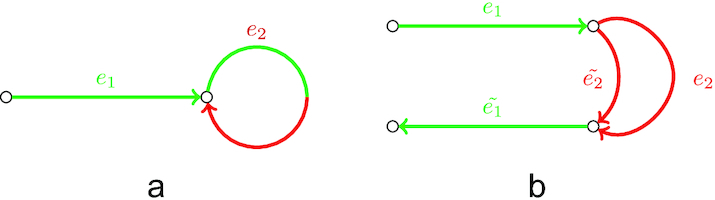
Examples of chimeric connections in the de Bruijn graph typical for transcriptome assembly. Red and green indicate erroneous and correct sequences, respectively. (a) A chimeric loop (edge *e*_2_) connecting the end of the correct transcriptomic edge *e*_1_ with itself. (b) An example of a chimeric hairpin, where erroneous edge *e*_2_ connects a correct edge *e*_1_ with its reverse-complement copy $\tilde{e_1}$. Because *e*_2_ connects a vertex and its reverse-complement, $\tilde{e_2}$ (the reverse-complement of *e*_2_) also connects these 2 vertices.

Our analysis revealed that most of the chimeric connections in RNA-Seq data can be divided into 2 groups: single-strand chimeric loops and double-strand hairpins. In the first case, a chimeric junction connects the end of a transcript sequence with itself (Fig. 4a). The erroneous hairpin connects a correct edge with its reverse-complement copy (Fig. 4b) and potentially may result in a chimeric palindromic sequence in the assembly. To avoid misassemblies, rnaSPAdes detects chimeric loops and hairpins by analyzing the graph topology rather than nucleotide sequences or coverage.

While it remains unclear whether these chimeric reads are formed during transcription, RNA-Seq sample preparation, or sequencing, similar chimeric connections have been observed in the context of single-cell MDA. For example, when a DNA fragment is amplified by MDA, the DNA polymerase moves along the DNA molecule and copies it, but sometimes (as described in [15]) the polymerase may jump to a close position (usually on the opposite DNA strand) and proceed to copy from the new position.

#### Removing isolated edges

Another type of excessive edges that appear in the assembly graph is isolated edges, i.e., that have no adjacent edges. They typically appear in regions of extremely low coverage (including DNA contamination), where overlaps between neighboring reads are smaller than *k*-mer size, or originate from reads containing zero correct *k*-mers owing to multiple sequencing errors. The first type of isolated edges can possibly be connected with other edges by means of a gap-closing procedure (described below). The second type, on the other hand, may result in excessive erroneous sequences in the assembly or even create ambiguities during gap closing. Thus, during graph simplification we additionally remove isolated edges that have both (i) low coverage (<2) and (ii) length smaller than or equal to read length.

#### Selecting optimal *k*-mer sizes

One of the key techniques that allows SPAdes to assemble contiguous genomic sequences from data with non-uniform coverage depth is the iterative de Bruijn graph construction. During each successive iteration, SPAdes builds the graph from the input reads and sequences obtained at the previous iteration, simplifies the graph, and provides its edges as input to the next iteration that uses larger *k*-mer size. The assembly graph obtained at the final iteration is used for repeat resolution and scaffolding procedures, which exploit read-pairs and long reads [36,39]. In this approach small *k*-mer sizes help to assemble low-covered regions where reads have short overlaps, and large *k*-values are useful for resolving repeats and therefore obtaining a less tangled graph. Although this method seems to be useful for restoring low-expressed isoforms from RNA-Seq data, our analysis revealed that it seems to be the main reason for the high number of misassembled contigs in SPAdes assemblies. Below we describe how these false junctions are formed.

**Figure 5:**
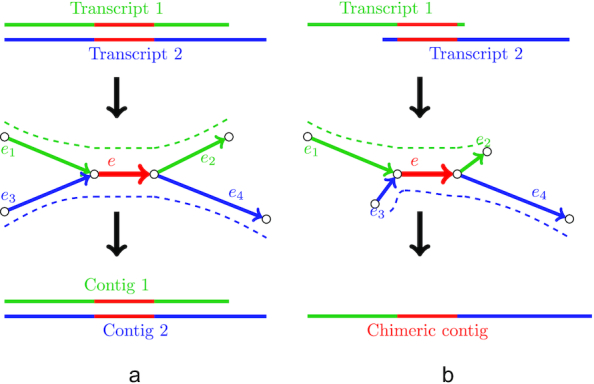
Examples of 2 transcripts having a common sequence (a) in the middle of the transcripts and (b) close to the start of one transcript and the end of another. While in the first case the isoforms can be resolved using read-pairs, the latter may potentially result in a chimeric contig.

When 2 transcripts (possibly from different genes) have a common sequence in the middle, they form a typical repeat structure in the de Bruijn graph (Fig. 5a), which may further be resolved, e.g., using paired reads. However, if a common sequence appears close to the ends of the transcripts (Fig. 5b), edges *e*_2_ and *e*_3_ appear to be rather short and may be trimmed as tips (because coverage depth often decreases near the transcripts' ends) or may not be present at all. In this case, the remaining edges *e*_1_, *e*, and *e*_4_ will be condensed into a single edge corresponding to chimeric sequence.

Indeed, because small *k*-mer size results in a higher chance of creating such a chimeric junction, we decided to modify the parameters for iterative graph construction. In rnaSPAdes we decided to use only 2 *k*-values: a smaller one for restoring low-covered regions with insufficient overlaps between reads and a larger one for obtaining a less tangled graph.

To estimate the optimal *k*-values, we ran rnaSPAdes on several RNA-Seq datasets with various read lengths sequenced from organisms with different gene complexity. Because it requires a tremendous amount of time to try all possible pairs of *k*-mer sizes on multiple datasets, we first estimated an upper *k*-value used for the main iteration and then selected the lower *k* with the fixed upper one.

We assembled a number of datasets using only a single *k*-mer size and selected the best assemblies according to number of assembled genes, database coverage, and number of misassemblies. Although it may not be possible to choose a single best *k*-value simultaneously for multiple datasets, nearly optimal *k*-mer size was estimated as half of the read length (more precisely, the largest odd number that does not exceed read_length/2 − 1). The smaller *k*-value was estimated in a similar manner with the fixed upper *k*-mer size. Optimal lower *k* was considered on the basis of the number of additional assembled genes and misassemblies. Experiments showed that small *k*-values (e.g., <29) tended to dramatically increase the number of erroneous contigs due to the higher probability of 2 transcripts sharing the same *k*-mer. Thus, the lower *k*-mer size was estimated approximately as read_length/3 with the minimum value set to 29. Although estimated *k*-values may not provide the best assembly for every dataset, they typically seem to be a good trade-off between the number of recovered genes and generated errors (see Supplementary Tables S7–S9).

In this work rnaSPAdes was launched with the default *k*-values. Indeed, rnaSPAdes retains the possibility of setting the *k*-mer sizes manually. While it is possible to set only 1 *k*-mer size, assemblies obtained with a single *k* typically capture fewer genes and isoforms (especially low-covered), but they also have fewer misassembled contigs (see Supplementary material Tables S9-S11 for comparison).

To preserve correct connections that could be restored using only small *k*-mer sizes, we carefully examined low-expressed transcripts that were not completely assembled using default *k*-mer sizes. The analysis revealed that the majority of such fragments can be joined by the small overlap, which is often confirmed by the read-pairs. To perform the gap-closing procedure rnaSPAdes glues 2 tips if 1 of the following conditions is true:

- tips have an exact overlap of length ≥*L*_ov_ and are connected by ≥*N*_ov_ read pairs;
- tips are connected by ≥*N*_min_ read pairs;

where the default parameters are *L*_ov_ = 8 bp, *N*_ov_ = 1, and *N*_min_ = 5. Although these parameters seem to be slightly ad hoc, such a gap-closing procedure seems to be a viable alternative to using small *k*-values and allows the restoration of more low-expressed transcripts without increasing the number of misassemblies. Using smaller thresholds for gap closing often creates false connections and increases the amount of erroneous transcripts, while larger values for these parameters result in a smaller increase of reconstructed sequences.

### Isoform reconstruction

#### Adapting repeat resolution algorithms

Genomic repeats present one of the key challenges in the *de novo* genome assembly problem. Although mRNA sequences typically do not contain complex repeats, transcriptome assembly has a somewhat similar problem of resolving alternatively spliced isoforms and transcripts from paralogous genes. Repeat resolution and scaffolding steps in the SPAdes genome assembler are implemented in the exSPAnder module [39], which is based on a simple path extension framework. Similar to other modules of SPAdes, exSPAnder was designed to deal with highly uneven coverage and thus can be adapted for the isoform detection procedure when assembling RNA-Seq data.

The key idea of the path extension framework is to iteratively construct paths in the assembly graph by selecting the best-supported extension edge at every step until no extension can be chosen. The extension is selected on the basis of the scoring function, which may exploit various kinds of linkage information between edges of the assembly graph (different scoring functions are implemented for different types of sequencing data). A situation when a path cannot be extended further is usually caused by the presence of a long genomic repeat or a large coverage gap. The extension procedure starts from the longest edge that is not yet included in any path and is repeated until all edges are covered.

More formally, a path extension step can be defined as follows. For a path *P* and its extension edges *e*_1_, …, *e_n_* (typically, edges that start at the terminal vertex of *P*) the procedure selects *e_i_* as a best-supported extension if

- Score_*P*_(*e_i_*) > *C* · Score_*P*_(*e_j_*) for all *j* ≠ *i*,
- Score_*P*_(*e_i_*) > Θ,

where *C* and Θ are the algorithm parameters and Score_*P*_(*e_i_*) is a score of edge *e_i_* relative to path *P* (described by Prjibelski et al. [39]).

In contrast to genome assembly, in which there is usually only 1 true extension edge, in transcriptome assembly multiple correct extensions are possible owing to the presence of alternatively spliced isoforms. Thus, the modified procedure is capable of selecting several edges $e_{k_1}, \ldots e_{k_m}$ among all possible extensions *e*_1_, …, *e_n_*, which satisfy the following conditions:

- $\mathrm{Score}_P({e_{k_i}}) \gt \mathrm{Score}_P({e_M}) / C$ for all *i* = 1…*m*,
  *M* = argmax_*j* = 1..*n*_Score_*P*_(*e_j_*),
- $\mathrm{Score}_P({e_{k_i}}) \gt \Theta$ for all *i* = 1…*m*.

Namely, all correct extension edges must have a score close to the maximal one (*C* = 1.5 by default), and the second condition remains the same. Afterwards, the algorithm extends path *P* by creating new paths $(P, e_{k_1}), \ldots , (P, e_{k_m})$, which are then extended independently. Because the scoring function implemented in exSPAnder does not strongly depend on the coverage depth, there is no danger that highly expressed isoforms will be preferred over the low-expressed ones.

Finally, to avoid duplicating sequences in the genome assemblies, exSPAnder performs a rather aggressive overlap removal procedure. However, because alternatively spliced isoforms may differ only by a short exon, in order to avoid missing similar transcripts the modified overlap detection procedure removes only exact duplicates and subpaths.

#### Exploiting coverage depth

Varying coverage depth may seem to be an additional challenge for *de novo* sequence assembly but can be also used as an advantage in some cases. For instance, if 2 alternatively spliced isoforms of the same gene have different expression levels, they can be resolved using coverage depth even when the read-pairs do not help (e.g., shared exon is longer than the insert size). Although using coverage values becomes more complicated when a gene has multiple different expressing isoforms, our analysis of several RNA-Seq datasets revealed that such cases are rather rare and most of the genes have 1 or 2 expressing isoforms within a single sample.

**Figure 6:**
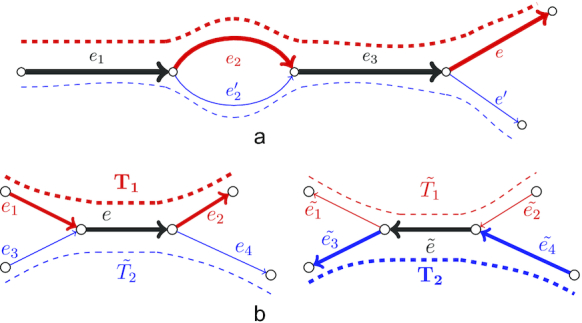
Using coverage depth for isoform reconstruction. Line width represents conventional and strand-specific coverage depths in panels (a) and (b), respectively. (a) Two isoforms of the same gene (red and blue dashed lines) have different expression levels and thus can be resolved using coverage depth. (b) Two transcripts *T*_1_ and *T*_2_ (red and blue bold dashed lines, respectively) share a reverse-complement sequence and thus can be resolved using strand-specific reads.

To exploit the coverage depth we decided to add a simple but reliable path extension rule. Let the path *P* = (*e*_1_, *e*_2_, *e*_3_) have extension edges *e* and *e*′ (Fig. 6a), such that cov(*e*) > cov(*e*′) and cov(*e*_2_) > cov($e_2^{\prime }$), where cov(*e*) denotes the *k*-mer coverage of edge *e*. To select a correct extension the algorithm detects a vertex closest to the end of path *P* that has 2 incoming alternative edges, 1 of which is included in *P* and the other is not (*e*_2_ and $e_2^{\prime }$ in this example). Because edge *e*_2_ ∈ *P* has higher coverage than the alternative edge $e_2^{\prime } \notin P$, we select extension edge *e* as the one with the higher coverage. However, if both isoforms have similar coverage, this simple approach may chose a false extension (because the coverage depth is rarely perfectly uniform even along a small region). Thus the difference in coverage should be significant enough to distinguish between the isoforms. More formally, the following conditions should be satisfied:

- cov(*e*) > Δ · cov(*e*′);
- cov(*e*_2_) > Δ · cov($e_2^{\prime }$);
- Ω > cov(*e*_2_)/cov(*e*) > 1/Ω;
- cov(*e*) > *C*_min_,

where the default values of the algorithm parameters are Δ = 2, Ω = 10, and *C*_min_ = 2. The first 2 conditions ensure that the extension edges (*e* and *e*′) and alternative edges (*e*_2_ and $e_2^{\prime }$) have significant coverage difference, condition iii requires the coverage depth to remain relatively persistent along the path, and condition iv prevents the algorithm from resolving low-covered isoforms (which may result in a misassembly). In the general case, this procedure also uses only the last pair of alternative edges and is applied only in the case when the path has 2 possible extension edges and the conventional read-pair extender fails to extend the path.

#### Assembling strand-specific data

Another possible way to improve a transcriptome assembly is to take advantage of strand-specific data when provided. To use stranded RNA-Seq we introduce "strand-specific coverage depths" cov^+^(*e*) and cov^−^(*e*), which denote *k*-mer coverage of edge *e* by forward and reverse reads, respectively. As opposed to the conventional coverage cov(*e*), which is calculated by aligning all reads and their reverse-complement copies to the edges of the assembly graph [thus making cov(*e*) = cov($\tilde{e}$)], strand-specific coverage is obtained by mapping reads according to their origin strand. For instance, if an RNA-Seq library is constructed in such a way that reads have the same strand as the transcript that they were sequenced from (sense/forward reads), we expect cov^+^(*e*) to be much higher than cov^−^(*e*) if the sequence of *e* corresponds to the transcript, and vice versa if *e* is the reverse-complement of the original transcript. Indeed, the situation becomes opposite when reads are sequenced from complementary DNAs that are reverse-complement to the original transcripts (anti-sense/reverse reads). When working with paired-end libraries, we assume that the type of library is defined by the first read’s strand (i.e., forward-reverse or reverse-forward). Thus, the second read in a pair is reverse-complemented before mapping in order to match the strand of the first read.

To extend the paths we apply the aforementioned path extension procedure for conventional coverage but use strand-specific coverage values instead. Fig. 6b demonstrates a situation when 2 transcripts correspond to paths *T*_1_ = (*e*_1_, *e, e*_2_) and $T_2 = (\tilde{e_4}, \tilde{e}, \tilde{e_3})$. If the repetitive edge *e* is longer than the insert size and the conventional coverage depth of these 2 transcripts is similar, the situation can be resolved neither by paired reads nor by coverage. However, in the case of stranded data, strand-specific coverage for actual transcripts’ paths will be much higher than for their reverse-complement copies, i.e., cov^+^(*T*_1_)≫ cov^+^($\tilde{T_1}$) and cov^+^(*T*_2_) ≫ cov^+^($\tilde{T_2}$) (in this example we assume that reads have the same stand as the transcripts they come from). Moreover, edges corresponding to the reverse-complement sequences only ($\tilde{e_1}$ and $\tilde{e_2}$ for $\tilde{T_1}$, *e*_3_ and *e*_4_ for $\tilde{T_2}$) will have cov^+^(*e*) values close to zero. Therefore, the conditions given for the coverage-based path extender (see previous subsection) will be satisfied for strand-specific coverage values, the repetitive edge *e* will be resolved, and both transcripts will be reconstructed.

To avoid collapsing transcripts from the opposite strands that share common sequences at their ends, we also split edges that have significantly different strand-specific coverage values at their ends. More formally, edge *e* is split at position *p* if cov^+^(*e*[0, *p*]) ≫ cov^−^(*e*[0, *p*]) and cov^−^(*e*[*p* + 1, length(*e*)]) ≫cov^+^(*e*[*p* + 1, length(*e*)]) (or vice versa), where *e*[*i, j*] is defined as a region of edge *e* starting from *i* and ending at *j*.

In addition, for stranded RNA-Seq data we output the paths constructed by the exSPAnder algorithm according to the original transcript’s strand. For example, in the example given in Fig. 6b rnaSPAdes will output paths *T*_1_ and *T*_2_ because they have higher strand-specific coverage than their reverse-complement copies ($\tilde{T_1}$ and $\tilde{T_2}$, respectively).

#### Filtering assembled transcripts

Before outputting the paths constructed by the exSPAnder module as contigs, we additionally apply various filtering procedures to remove non-mRNA contigs, such as intergenic sequences, which often contaminate RNA-Seq datasets. Our analysis showed that the majority of such unwanted sequences have low coverage and relatively small length and often correspond to isolated edges in the assembly graph. However, applying filters based on these criteria may also remove correct low-expressed transcripts in some cases. Thus, we decided to implement 3 different presets of parameters for the filtration procedure (soft, normal, and hard) and output 3 files with contigs (see exact parameters in Supplementary Table S12). Depending on the project goal the researcher may choose more sensitive (soft filtration) or more specific results (hard filtration). Table S13 in the Supplementary material shows how the assembly quality depends on the filtration parameters. In other tables we use default transcripts with the normal level of filtering.

## Availability of supporting source code and requirements

Project name: rnaSPAdes

Project home page: cab.spbu.ru/software/rnaspades/, github.com/ablab/spades

Operating systems: Linux and MacOS

Programming language: C++, Python

Other requirements: no requirements for precompiled binaries; g++ 5.3.1+, cmake 2.8.12+, zlib, and libbz2 are required for compiling from source code

License:GPLv2

RRID:SCR_016992

## Availability of supporting data and materials

All real RNA-Seq datasets are available at the NCBI SRA (https://www.ncbi.nlm.nih.gov/sra) with the following accession numbers:

- Human: SRR5133163
- Human large: SRR1957703, SRR1957706
- Mouse: SRX648736
- Worm: SRR1560107
- Corn: SRR1588569
- Arabidopsis: SRR5344669, SRR5344670

Simulated data are available on the server:

*H. sapiens*: http://spades.bioinf.spbau.ru/rnaspades/simulated_data/human/

*M. musculus*: http://spades.bioinf.spbau.ru/rnaspades/simulated_data/mouse/

An archival copy of the code and other supporting data is available via the *GigaScience database*, GigaDB [42].

## Additional files

**Supplementary information**: Supplementary Methods and Results are available via the additional file associated with this article. Addtional file contains the following information:

Section S1: Transcriptome assembly quality evaluation metrics;

Section S2: Software versions used in this work;

Section S3: Command lines for reproducing the analysis;

Tables S1–S2: Complete quality reports for all simulated datasets;

Tables S3–S7: Complete quality reports for all real datasets;

Figure S1: Additional statistics for simulated datasets;

Figure S2: BUSCO results for all datasets;

Figures S3–S5: The most representative metrics reported by rnaQUAST, DETONATE and Transrate across all datasets;

Tables S9–S11: rnaSPAdes results for different k-mer sizes;

Table S12: Graph simplification statistics;

Table S13: Path filtration parameters;

Table S14: rnaSPAdes results for different filtration levels;

Table S15: Kallisto results for all datasets.

giz100_GIGA-D-18-00456_Original_Submission

giz100_GIGA-D-18-00456_Revision_1

giz100_GIGA-D-18-00456_Revision_2

giz100_GIGA-D-18-00456_Revision_3

giz100_Response_to_Reviewer_Comments_Original_Submission

giz100_Response_to_Reviewer_Comments_Revision_1

giz100_Response_to_Reviewer_Comments_Revision_2

giz100_Reviewer_1_Report_Original_Submission Camille Marchet -- 12/10/2018 Reviewed

giz100_Reviewer_2_Report_Original_Submission Zhong Wang -- 12/17/2018 Reviewed

giz100_Reviewer_3_Report_Original_Submission Marcel Schulz -- 12/19/2018 Reviewed

giz100_Reviewer_3_Report_Revision_1 Marcel Schulz -- 5/17/2019 Reviewed

giz100_Reviewer_4_Report_Original_Submission Christian Cole -- 12/20/2018 Reviewed

giz100_Reviewer_4_Report_Revision_1 Christian Cole -- 12/20/2018 Reviewed

giz100_Supplemental_File

## Abbreviations

BUSCO: Benchmarking Universal Single-Copy Orthologs; MDA: multiple displacement amplification; mRNA: messenger RNA; CRBB: Conditional Reciprocal Best BLAST; NCBI: National Center for Biotechnology Information; RAM: random access memory; RNA-Seq: RNA sequencing; SPAdes: St. Petersburg genome assembler; SRA: Sequence Read Archive.

## Competing interests

The authors declare that they have no competing interests.

## Funding

This work was supported by the Russian Foundation for Basic Research (grant No. 19-04-01074) and St. Petersburg State University (grant No. 15.61.951.2015).

## Authors' contributions

Software design and implementation was performed by E.B., D.A., and A.D.P. E.B. was responsible for data curation, assembler benchmarking, and manuscript editing. A.L. supervised the project and performed funding acquisition. A.D.P. wrote the manuscript and managed the project. All authors read and approved the final manuscript.
