# Supplementary material for: rnaSPAdes: a de novo transcriptome assembler and its application to RNA-Seq data
Source: Gigascience. 2019 Sep 3;8(9):giz100. doi: 10.1093/gigascience/giz100 (PMC6736328; doi:10.1093/gigascience/giz100)
Supplement: giz100_GIGA-D-18-00456_Original_Submission [file giz100_giga-d-18-00456_original_submission.pdf]

GigaScience

rnaSPAdes: a de novo transcriptome assembler and its application to RNA-Seq data

--Manuscript Draft--

**Manuscript Number:**	GIGA-D-18-00456
**Full Title:**	rnaSPAdes: a de novo transcriptome assembler and its application to RNA-Seq data
**Article Type:**	Research
**Funding Information:**	Russian Science Foundation (RU) (14-50-00069) — Not applicable
**Abstract:**	Possibility to generate large RNA-Seq datasets has led to development of various reference-based and de novo transcriptome assemblers with their own strengths and limitations. While reference-based tools are widely used in various transcriptomic studies, their application is limited to the organisms with finished and well-annotated genomes. De novo transcriptome reconstruction from short reads remains an open challenging problem, which is complicated by the varying expression levels across different genes, alternative splicing and paralogous genes. In this paper we describe a novel transcriptome assembler called rnaSPAdes, which is developed on top of SPAdes genome assembler and explores surprising computational parallels between assembly of transcriptomes and single-cell genomes. We also present quality assessment reports for rnaSPAdes assemblies, compare it with modern transcriptome assembly tools using several evaluation approaches on various RNA-Seq datasets, and briefly highlight strong and weak points of different assemblers.
**Corresponding Author:**	Andrey D. Prjibelski, M.Sc.<br>SPbU<br>St. Petersburg, Russia RUSSIAN FEDERATION
**Corresponding Author Secondary Information:**	
**Corresponding Author's Institution:**	SPbU
**Corresponding Author's Secondary Institution:**	
**First Author:**	Elena Bushmanova, M.Sc.
**First Author Secondary Information:**	
**Order of Authors:**	Elena Bushmanova, M.Sc.
	Dmitry Antipov, M.Sc.
	Alla Lapidus, Ph. D.
	Andrey D. Prjibelski, M.Sc.
**Order of Authors Secondary Information:**	
**Additional Information:**	
**Question**	**Response**
Are you submitting this manuscript to a special series or article collection?	No
**Experimental design and statistics**<br><br>Full details of the experimental design and statistical methods used should be given in the Methods section, as detailed in our Minimum Standards Reporting Checklist. Information essential to interpreting the	Yes

Powered by Editorial Manager® and ProduXion Manager® from Aries Systems Corporation

data presented should be made available in the figure legends.<br><br>Have you included all the information requested in your manuscript?	
**Resources**<br><br>A description of all resources used, including antibodies, cell lines, animals and software tools, with enough information to allow them to be uniquely identified, should be included in the Methods section. Authors are strongly encouraged to cite Research Resource Identifiers (RRIDs) for antibodies, model organisms and tools, where possible.<br><br>Have you included the information requested as detailed in our Minimum Standards Reporting Checklist?	Yes
**Availability of data and materials**<br><br>All datasets and code on which the conclusions of the paper rely must be either included in your submission or deposited in publicly available repositories (where available and ethically appropriate), referencing such data using a unique identifier in the references and in the “Availability of Data and Materials” section of your manuscript.<br><br>Have you have met the above requirement as detailed in our Minimum Standards Reporting Checklist?	Yes

Click here to download Manuscript rnaSPAdes.pdf

OXFORD

(GIGA)^n SCIENCE

GigaScience, 2018, 1–11

doi: xx.xxxx/xxxx
Manuscript in Preparation
Paper

PAPER

rnaSPAdes: a *de novo* transcriptome assembler and its application to RNA-Seq data

Elena Bushmanova[1], Dmitry Antipov[1], Alla Lapidus[1] and Andrey D. Prjibelski[1*]

[1]Center for Algorithmic Biotechnology, Institute of Translational Biomedicine, St. Petersburg State University, St. Petersburg, Russia

[*]To whom correspondence should be addressed: andrewprzh@gmail.com

Abstract

Possibility to generate large RNA-Seq datasets has led to development of various reference-based and *de novo* transcriptome assemblers with their own strengths and limitations. While reference-based tools are widely used in various transcriptomic studies, their application is limited to the organisms with finished and well-annotated genomes. *De novo* transcriptome reconstruction from short reads remains an open challenging problem, which is complicated by the varying expression levels across different genes, alternative splicing and paralogous genes. In this paper we describe a novel transcriptome assembler called rnaSPAdes, which is developed on top of SPAdes genome assembler and explores surprising computational parallels between assembly of transcriptomes and single-cell genomes. We also present quality assessment reports for rnaSPAdes assemblies, compare it with modern transcriptome assembly tools using several evaluation approaches on various RNA-Seq datasets, and briefly highlight strong and weak points of different assemblers.

Key words: RNA-Seq; *de novo* assembly; transcriptome assembly

Background

While reference-based methods for RNA-Seq analysis [1, 2, 3, 4, 5, 6] are widely used in transcriptome studies, they are subjected to the following constraints: (i) they are not applicable in the case when the genome is unknown, (ii) their performance deteriorates when the genome sequence or annotation are incomplete, and (iii) they may miss unusual transcripts such as fusion genes or genes with short unannotated exons. To address these constraints, *de novo* transcriptome assemblers [7, 8, 9, 10, 11] have emerged as a viable complement to the reference-based tools. Although *de novo* assemblers typically generate fewer complete transcripts than the reference-based methods for the organisms with accurate reference sequences [12], they may provide additional insights on aberrant transcripts.

While the transcriptome assembly may seem to be a simpler problem than the genome assembly, RNA-Seq assemblers have to address the complications arising from highly uneven read coverage depth caused by variations in gene expression levels. However, this is the same challenge that we have addressed while developing SPAdes assembler [13, 14], which originally aimed at single-cell sequencing. Similarly to RNA-Seq, the Multiple Displacement Amplification (MDA) technique [15], used for genome amplification of single bacterial cells, results in a highly uneven read coverage. In the view of similarities between RNA-seq and single-cell genome assemblies, we decided to test SPAdes without any modifications on transcriptomic data. Even though SPAdes is a genome assembler and was not optimized for RNA-seq data, in some cases it generated decent assemblies of quality comparable to the state-of-the-art transcriptome assemblers.

To perform the benchmarking we have used rnaQUAST tool [16], which was designed for quality evaluation of *de novo* assemblies with the support of reference genome and its gene database. For the comparison, we selected a few representative metrics such as (i) total number of assembled transcripts (contigs), (ii) reference gene database coverage, (iii) number of

Compiled on: November 16, 2018.
Draft manuscript prepared by the author.

Table 1. Benchmarking of **BinPacker**, **Bridger**, **IDBA**-tran, **RNA-Bloom**, **SOAP**denovo-Trans, Trans-**ABySS**, **Trinity**, and **SPAdes** on *C. elegans* RNA-seq dataset (accession number SRR1560107, 9 million Illumina 90 bp long paired-end reads). All contigs shorter than 200 bp were filtered out prior to the analysis. The annotated transcriptome of *C. elegans* WBcel235.82 consists of 46748 genes and 57834 isoforms. The best values for each metric are highlighted with bold.

	BinPacker	Bridger	IDBA	RNA-Bloom	SOAPdenovo	ABySS	Trinity	**SPAdes**
Transcripts	20460	22147	23172	29995	18801	46145	24428	21235
Database coverage, %	33.3	33.7	33.2	**37.9**	32.1	37.7	36.1	33.3
50%-assembled genes	9907	9950	10075	10028	9867	10130	**10394**	10378
95%-assembled genes	5666	5654	3786	5390	5155	5413	5682	**5948**
50%-assembled isoforms	10398	10352	10227	**11639**	9995	11058	11127	10426
95%-assembled isoforms	5910	5874	3788	**6100**	5198	5487	6030	5949
Misassemblies	294	256	40	96	**28**	37	217	130
Duplication ratio	1.20	1.14	1.01	1.59	1.26	1.68	1.69	**1.0**

50% / 95%-assembled genes/isoforms, (iv) number of misassemblies and (v) duplication ratio. The detailed description for these metrics can be found in the Supplementary material.

Table 1 demonstrates the comparison between different assembly tools on publicly available *C. elegans* RNA-Seq dataset. All transcriptome assemblers were launched with default parameters, SPAdes was run in single-cell mode due to the uneven coverage depth of RNA-Seq data. Table 1 shows that SPAdes generates more 95%-assembled genes than any other tool, and has comparable number of 50%-assembled genes and moderate gene database coverage. At the same time, SPAdes produces rather high number of misassembled transcripts, which can be explained by the fact that algorithms for genome assembly tend to assemble longer contigs and may incorrectly join sequences corresponding to different genes when working with RNA-Seq data. In addition, SPAdes generates almost the same number of 95%-assembled genes and isoforms, which emphasizes the lack of isoform detection step.

Benchmarking on other datasets also showed that SPAdes successfully deals with non-uniform coverage depth and produces relatively high number of 50% / 95%-assembled genes in most cases. However, it also confirmed the problem of large amount of erroneous transcripts as well as relatively low number of fully reconstructed alternative isoforms in SPAdes assemblies. Based on the obtained statistics we decided to adapt current SPAdes algorithms for RNA-Seq data with the goal to improve quality of generated assemblies and develop a new transcriptomic assembler called rnaSPAdes. In this manuscript we describe major pipeline modifications as well as several algorithmic improvements introduced in rnaSPAdes that allow to avoid misassemblies and obtain sequences of alternatively spliced isoforms.

To perform sufficient benchmarking of rnaSPAdes and other transcriptome assemblers mentioned above, we assembled several simulated and publicly available real RNA-Seq datasets from the organisms with various splicing complexity. For the generated assemblies we present quality assessment reports obtained with different *de novo* and reference-based evaluation approaches. In addition, based on these results we discuss superiorities and disadvantages of various assembly tools and provide insights on their performance.

Data Description

To compare rnaSPAdes performance with the state-of-the-art transcriptome assemblers we selected 2 simulated and 4 real publicly available RNA-Seq datasets with different characteristics, which include (i) read length, (ii) dataset size, (iii) strand-specificity and (iv) organism splicing complexity.

Data, simulated using RSEM simulator [1]:

- *H. sapiens*, non-strand-specific, 30 million simulated Illumina 150 bp paired-end reads;
- *M. musculus*, non-strand-specific, 11 million simulated Illumina 101 bp paired-end reads.

Real RNA-Seq datasets:

- *H. sapiens*, non-strand-specific, 30 million Illumina 150 bp paired-end reads;
- *M. musculus*, non-strand-specific, 11 million Illumina 101 bp paired-end reads;
- *C. elegans* non-strand-specific, 9 million Illumina 90 bp paired-end reads;
- *Z. mays* strand-specific, 8 million Illumina 100 bp long paired-end reads.

During the development rnaSPAdes was tested on the larger variety of RNA-Seq data. Although 6 datasets used in this manuscript may not represent all kinds of transcriptomic data, they are sufficient for comparing different assembly tools and detecting their superiorities and disadvantages.

Analyses

Selected datasets were assembled with BinPacker [17], Bridger [18], IDBA-tran [10], RNA-Bloom [19], SOAPdenovo-Trans [11], Trans-ABySS [7], Trinity [8] and rnaSPAdes using default parameters, and SPAdes [13] in single-cell mode. For a fair comparison the same minimal contig length cutoff was used for all tools (200 bp). For the assemblers that have no such option, sequences shorter than 200 bp were filtered out manually. To evaluate the resulting assemblies we used rnaQUAST [16], Transrate [20], BUSCO [21] and DETONATE [22]. From each tools we selected a few representative metrics that would allow to perform complete comparison between different tools. To make the results reproducible, we also provide software versions and command lines used in this study in the Supplementary material.

Evaluating assemblers on simulated data

To simulate RNA-Seq dataset we used RSEM simulator [1], which allows to generate reads based on the real RNA-Seq data. For this purpose we selected *H. sapiens* and *M. musculus* datasets (SRR5133163 and SRX648736). The resulting simulated datasets contain 30M and 11M paired-end reads respectively. Table 2 shows short quality assessment report for *H. sapiens* data. Complete evaluation reports for both simulated dataset are presented in the Supplementary material.

Table 2 shows that rnaSPAdes pipeline produces the highest number of 95%-assembled genes and isoforms, with Trinity and RNA-Bloom being the closest competitors. Trinity and RNA-Bloom also have the highest gene database cover-

Table 2. Benchmarking of **BinPacker**, **Bridger**, **IDBA**-tran, **RNA-Bloom**, **SOAP**denovo-Trans, Trans-**ABySS**, **Trinity**, **SPAdes** and **rnaSPAdes** on *H. sapiens* simulated RNA-seq dataset (30 million Illumina 150 bp long paired-end reads). All contigs shorter than 200 bp were filtered out prior to the analysis. The annotated transcriptome of *H. sapiens* GRCh37.p13 consists of 57820 genes and 196520 isoforms. The best values for each metric are highlighted with bold.

	BinPacker	Bridger	IDBA	RNA-Bloom	SOAP	ABySS	Trinity	SPAdes	rnaSPAdes
Transcripts	76736	52151	58466	65968	35096	67511	62831	42264	40262
Database coverage, %	20.9	18.5	21.4	**24.6**	19.4	23.1	24.4	20.5	23.0
50%-assembled genes	11828	11476	13175	12869	12610	12740	13289	13569	**14341**
95%-assembled genes	7320	6417	2729	8910	7685	7225	9049	8526	**10640**
50%-assembled isoforms	17415	15423	18181	**21035**	15638	19250	20965	16437	19452
95%-assembled isoforms	9091	7298	2744	12108	8151	7662	12301	8638	**12604**
Misassemblies	7919	3512	174	358	198	**126**	1554	443	292
Duplication ratio	2.19	1.38	1.01	1.93	1.08	1.24	1.74	**1.0**	1.18

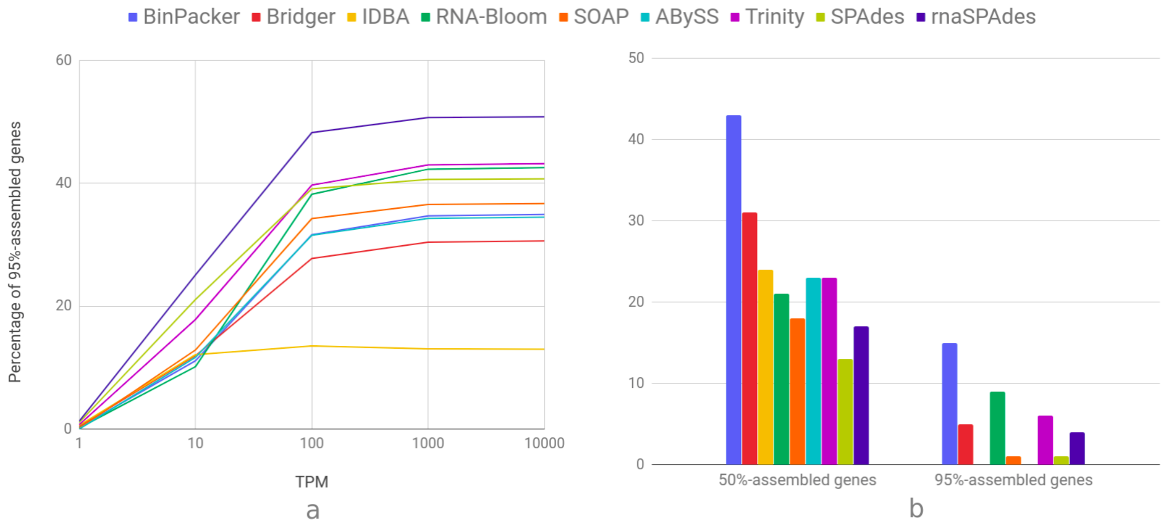

Figure 1. (a) Cumulative plot showing how fraction of 95%-assembled genes in each assembly depends on the gene coverage by reads in TPM (Transcripts Per Kilobase Million) reported by RSEM simulator. (b) Number of 50%/95%-assembled genes in each assembly that have zero reads generated by RSEM simulator (i.e. falsely assembled genes). The plots are constructed for *H. sapiens* simulated dataset.

age, while rnaSPAdes and Trans-ABySS are just slightly behind (about 1.5% less nucleotides covered). However, both Trinity and RNA-Bloom seem to produce a lot of excessive sequences (high duplication ratio), and Trinity also appears to be somewhat inaccurate in terms of misassembled sequences (5 times more than rnaSPAdes). Among the tools with high number of assembled genes and isoforms, Trans-ABySS and SOAPdenovo-Trans are the most accurate, while rnaSPAdes and RNA-Bloom follow with the moderate number of misassembled contigs. Although IDBA also generates an accurate assembly, it appears to be fragmented (small number of 95%-assembled genes and isoforms). Both BinPacker and Bridger produce vast number of errors and relatively high duplication ratio.

Since RSEM simulator provides read count for each particular gene, we also computed the number of assembled genes reported by rnaQUAST depending on their read coverage (Fig. 1a). The figure demonstrates that rnaSPAdes, SPAdes and Trinity outperform other tools on low-abundant transcripts, with rnaSPAdes reaching the highest fraction of total 95%-assembled genes (50.8%). In addition, for each assembler we provide the number of genes that were assembled, but in fact were not simulated by RSEM, i.e. have no corresponding reads (Fig. 1b). Although BinPacker and Bridger have the highest absolute amount of such genes, all tools produce insignificant faction of falsely assembled genes (far below 1%).

Evaluating assemblers on real RNA-Seq data

For comparison on real RNA-Seq reads we selected *H. sapiens*, *M. musculus* and *C. elegans* non-stranded datasets and *Z .mays* strand-specific dataset. Short report for *M. musculus* assemblies is shown in Table 3, while complete reports for all data are presented in the Supplementary material (Tables S3-S6 respectively). In addition, we added BUSCO reports (Figure 2, Supplementary Figure S1) and presented various metrics as bar plots (Figure 3, Supplementary Figures S3-S5).

Table 3 indicates, that while all assemblies have comparable amount of 50%-assembled genes and isoforms, rnaSPAdes, SPAdes and Trinity have the best values for database coverage and number of 95%-assembled genes and isoforms. Among these assemblers, rnaSPAdes dominates by these parameters: 14% and 5% more 95%-assembled genes, 8% and 6% more 95%-assembled isoforms than Trinity and SPAdes respectively. Figure 3 shows that Trinity, SPAdes and rnaSPAdes are among top assemblers regarding the amount of 95%-assembled genes across all datasets, while other tools seem to be less stable.

Similarly to the simulated dataset (Table 2), Table 3 shows that BinPacker, Bridger, RNA-Bloom and Trinity generate transcripts with high misassembly and duplication rates. SOAPdenovo-Trans produces the most accurate assembly according to these parameters, but has rather fragmented assembly (one of the smallest values for assembled genes and isoforms). rnaSPAdes manages to maintains the appropriate levels of duplication ratio (13% less than Trinity) and the number of misassemblies (being third after SOAPdenovo-Trans and

Table 3. Benchmarking of **BinPacker**, **Bridger**, **IDBA**-tran, **RNA-Bloom**, **SOAP**denovo-Trans, Trans-**ABySS**, **Trinity**, **SPAdes** and **rnaSPAdes** on *M. musculus* non-strand-specific RNA-seq dataset (accession number SRX648736, 11 million Illumina 101 bp long paired-end reads). All contigs shorter than 200 bp were filtered out prior to the analysis. The annotated transcriptome of *M. musculus* GRCm38.75 consists of 38924 genes and 94545 isoforms. The best values for each metric are highlighted with bold.

	BinPacker	Bridger	IDBA	RNA-Bloom	SOAP	ABySS	Trinity	SPAdes	rnaSPAdes
Transcripts	27234	42029	38313	46440	31878	36488	47746	42949	47852
Database coverage, %	14.4	16.3	16.9	13.8	15.1	16.2	18.2	17.7	**18.7**
50%-assembled genes	6005	6090	6558	4859	6241	6321	6633	6890	**7103**
95%-assembled genes	1917	1909	1602	1256	1653	1798	2272	2450	**2587**
50%-assembled isoforms	6360	6451	6790	5591	6376	6931	**7386**	7053	7371
95%-assembled isoforms	1992	1982	1602	1346	1655	1850	2406	2450	**2609**
Misassemblies	947	923	387	732	**37**	194	459	497	236
Duplication ratio	1.12	1.09	**1.00**	1.33	**1.00**	1.09	1.15	**1.00**	1.02

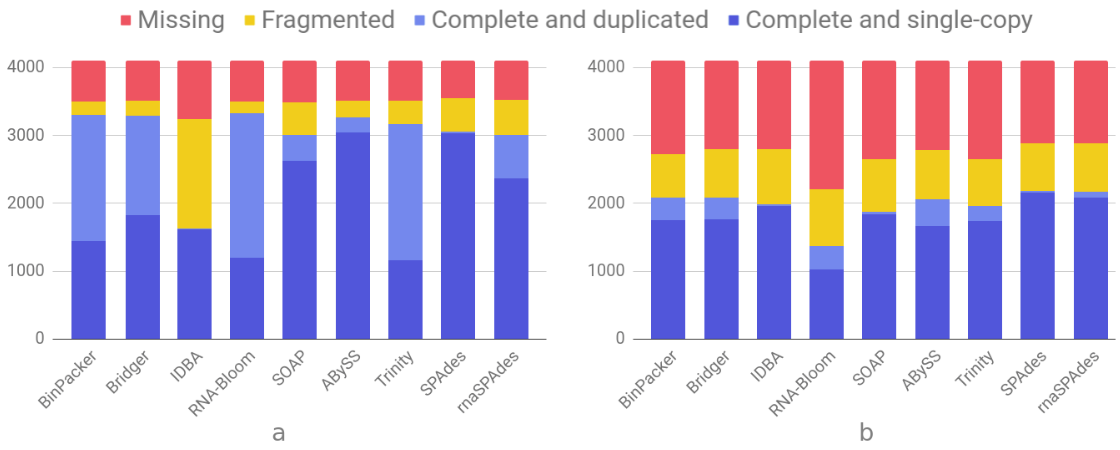

Figure 2. BUSCO results for (a) *H. sapiens* and (b) *M. musculus* assemblies. Dark blue indicates complete and single-copy genes, light blue — complete and duplicated, yellow — fragmented and red corresponds to missing BUSCOs. For both organisms BUSCO mammalian gene database was used

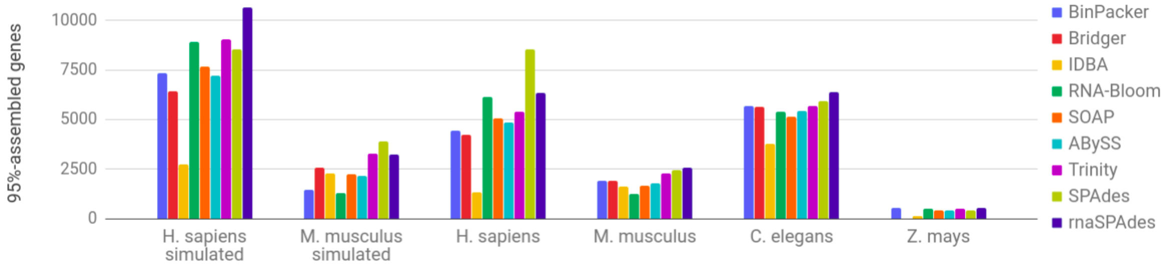

Figure 3. The number of 95%-assembled genes reported by rnaQUAST presented as bar plots for all generated assemblies. Note, that Bridger failed to assemble *Z. mays* dataset.

Trans-ABySS). Benchmarking with BUSCO (Figure 2) does not provide a clear winner, but confirms the same problem of high duplication levels for BinPacker, Bridger, RNA-Bloom and Trinity.

Computational performance

To compare selected assemblers in terms of computational performance, we measured their running time and RAM consumption using system utilities (rather than using log files). As Table 4 indicates, SOAPdenovo-Trans is significantly faster than any other assembler. Trans-ABySS, IDBA-tran, RNA-Bloom and rnaSPAdes have comparable performance on average, with rnaSPAdes being slightly faster and more greedy regarding RAM consumption on average. The other three tools showed longer running time and higher memory requirements.

Discussion

Quality reports provided in this manuscript (Tables 2 and 3) and Supplementary material (Tables S1-S6, Figures S1-S5) contain huge variety of different assembly characteristics. Below we attempt to summarize these results and highlight strong and weak points of different assemblers.

Sensitivity

rnaSPAdes typically shows high number of captured genes, which is indicated by the number of 50%/95%-assembled genes, amount of reference proteins with CRBB hits and complete BUSCOs detected. Based on the simulated datasets, both SPAdes and rnaSPAdes allow to restore more low-covered genes, which proves the efficiency of algorithms designed for highly uneven coverage depths. In comparison to original genomic SPAdes, rnaSPAdes typically assembles more genes and isoforms, has higher database coverage and fewer misassem-

Table 4. Running time and peak RAM usage for different assemblers on *M. musculus* and *H. sapiens* RNA-seq datasets (accession numbers SRX648736 and SRR5133163 respectively). The mouse dataset contains 11 million Illumina 101 bp long paired-end reads; the human dataset consists of 30 million Illumina 150 bp long paired-end reads. All assemblers we launched in 16 threads on a server with 128 GB of RAM and 56 Intel Xeon 2.0 GHz cores. BinPacker, which has no options for setting the number of threads, was launched with default parameters. For rnaSPAdes we also provide running time and peak memory for its submodules: read error **correction** using BayesHammer [23] and the **assembly** itself. The best values for time and RAM are highlighted with bold.

	M. musculus		*H. sapiens*	
Assembler	Time	RAM	Time	RAM
BinPacker	3 h 57 m	21 GB	21 h 48 m	65 GB
Bridger	2 h 36 m	19 GB	19 h 39 m	63 GB
IDBA	51 m	7 GB	2 h 56 m	20 GB
RNA-Bloom	56 m	12 GB	5 h 46 m	**19 GB**
SOAP	**9 m**	10 GB	**33 m**	28 GB
ABySS	31 m	**5 GB**	3 h 54 m	25 GB
Trinity	2 h 42 m	7 GB	6 h 24 m	64 GB
rnaSPAdes	30 m	9 GB	2 h 54 m	47 GB
Correction	16 m	9 GB	1 h 58 m	47 GB
Assembly	14 m	9 GB	56 m	28 GB

blies (except for the human assembly where SPAdes outperforms all tools). However, rnaSPAdes always has larger number of duplicated sequences and lower Detonate precision metrics.

Also, rnaSPAdes assemblies have rather decent characteristics with respect to gene coverage (i.e. how well reference genes are covered by *all* contigs), which is represented by such metrics as gene database coverage (rnaQUAST), nucleotide recall (Detonate), reference coverage and 50%/95%-covered proteins (Transrate). The best values for these metrics are often belong to RNA-Bloom, Trans-ABySS and Trinity. However, these tools with rather high sensitivity have their own drawbacks (described below). For example, Trans-ABySS assemblies appear to be rather fragmented (in comparison to rnaSPAdes and Trinity), with typically lower number of 95%-assembled genes and isoforms. Other assemblers have comparable database coverage and other sensitivity characteristics, with IDBA-trans producing the smallest number of 95%-assembled genes and isoforms in most cases.

Nucleotide and contig recall metrics reported by Detonate generally support the conclusions stated above. Thus, rnaSPAdes and Trinity have the best nucleotide recall values. Although most of the tools have comparable contig recall, Trinity, RNA-Bloom and Trans-ABySS have the highest values on average. To compute contig metrics Detonate keeps only the most reliable alignments with mapped fraction more than 99% (for both assembled and reference sequence). To compute the number of X%-assembled genes/isoforms rnaQUAST, on the other hand, assigns contigs to known genes/isoforms and then counts ones that have at least X% covered by a single assembled contig. However, no cutoff is applied for mapped fraction of the assembled sequences. This difference in algorithms might explain the absence of perfect correlation between contig recall and number of 95%-assembled isoforms.

Accuracy and specificity

The most accurate assemblies are generated by SOAPdenovo-Trans, which is confirmed by the values of Detonate precision metrics, duplication ratio, amount of duplicated BUSCOs, number of potentially segmented contigs reported by Transrate and missassemblies detected by rnaQUAST. IDBA, Bridger and genomic SPAdes also have high nucleotide precision, but feature significantly more assembly errors than SOAPdenovo-Trans. In addition, Bridger produces duplicated sequences (high duplication ration, a lot of duplicated BUSCOs) and IDBA has lowest average contig precision (due to fragmented assembly).

BinPacker, RNA-Bloom Trinity and Bridger (and Trans-ABySS in some cases) tend to produce a lot of excessive sequences, which result in the large numbers of duplicated BUSCOs, highest duplication ratios (rnaQUAST) and percentage of contig bases not covered by reads (Transrate). In addition, Trinity, BinPacker and Bridger generate relatively large number of misassemblies, while RNA-Bloom, rnaSPAdes and Trans-ABySS keep the moderate amount of chimeric sequences on average. Indeed, erroneous and duplicated sequences may negatively affect further transcriptome analysis, such as annotation.

Read-based scores

According to the read-based scores reported by Transrate and Detonate RSEM EVAL, which represent how well the assembly corresponds to the initial reads, rnaSPAdes also produces decent results. Regarding the average Transrate contig score, rnaSPAdes (average score 0.277) only loses to usual SPAdes (0.318). As to Detonate score, rnaSPAdes has the third-best average ($-1.57 \cdot 10^9$) and appears to be behind RNA-Bloom ($-1.47 \cdot 10^9$) and Trinity ($-1.51 \cdot 10^9$), which, on the other hand, have the lowest Transrate average scores among all tools (0.101 and 0.152 respectively). Vice versa, SOAPdenovo-Trans and SPAdes are among top 3 by Transrate score (which seems to correlate with duplication level), but the rather low RSEM EVAL scores.

Conclusion

Although every transcriptome assembler presented in this study has its own benefits and drawbacks, the trade-off between specificity and sensitivity can be significantly shifted by modifying the algorithms' parameters. For example, various thresholds for transcripts filtration in rnaSPAdes (Table S11 in the Supplementary material) result in assemblies with different properties (more specific assemblies for aggressive filtration and more sensitive for relaxed parameters). Also, varying k-mer size or incorporating iterative de Bruijn graph construction in rnaSPAdes may significantly vary accuracy and sensitivity (Tables S7-S9 in the Supplementary material). Thus, the parameters of the assembly algorithms can be varied in order to achieve the desired sensitivity or specificity characteristics and make the method to dominate by a certain group of metrics.

Reports presented in this manuscript include large variety of metrics, which reflect completely different assembly properties, importance of which may vary depending on the further analysis and the entire pipeline being used. We believe that one of the key features of the *de novo* transcriptome assembler is the ability to correctly capture the entire transcript into a single contig (e.g. reflected by the number of 95%-assembled genes/isoforms, contig recall). On the other hand, such metrics as gene database coverage, number of covered reference proteins and nucleotide recall do not reflect this significant property, since they account all contigs mapped to a specific gene or protein and do not include assembly contiguity. For example, high database coverage or nucleotide recall can be achieved by a very fragmented assembly (or even raw reads), which, indeed, does not suit well for further reference-free analysis.

While the developed algorithm, rnaSPAdes, typically shows stable and decent results across analyzed RNA-Seq datasets, there is no clear winner according to all metrics. Thus, the selection of the assembler may be varied depending on the goals

of the particular research project and the sample preparation protocols being used, as well as secondary parameters, such as usability and computational performance. Even with the aid of specially developed tools, such as Transrate, DETONATE, BUSCO and rnaQUAST, the choice of a suitable assembly tool remains a non-trivial problem and may require additional benchmarks in each particular case.

Potential implications

Although the developed approach was initially designed for RNA-Seq data obtained from a single organism, it can be also applied for metatranscriptome assembly of samples collected from bacterial communities. Indeed, metatranscriptome assembly does not require reconstructing complex alternatively spliced isoforms, but implies other computational challenges, such as repetitive patterns in different genes (including homologous genes from various strains) and extreme differences in mRNA quantities [24, 25], which are caused by both — varying expression levels and abundances of different species. rnaSPAdes was launched on real and synthetic metatranscriptomic data, and produced rather decent assemblies according to the initial analysis. Thus, improving the assembly algorithms, as well as designing an appropriate pipeline for quality evaluation of metatranscriptomic assemblies, are the main possible further implications of this work.

Recently emerged long read protocols for mRNA sequencing allow to capture full-length transcripts without the assembly [26]. However, high error rate of Oxford Nanopore and PacBio sequencers prevents using output reads directly as complete transcripts. Typically, mapping to the reference genome, additional error-correction by short accurate Illumina reads or consensus construction is performed to obtain and further analyze high-quality sequences [27, 28, 29, 30, 31]. Combining rnaSPAdes with previously developed hybridSPAdes approach for joint assembly of short and long reads [32] may result into a viable alternative to the existing methods for processing long error-prone RNA reads.

In addition, benchmarking reports presented in this work can be used by the researchers for selecting the appropriate assembly method that meets their specific criteria and for better understanding of transcriptome assembly quality evaluation.

Methods

Most of the modern *de novo* genome assembly algorithms for short reads rely on the concept of the de Bruijn graph [33]. While the initial study proposed to look for an Eulerian path traversing the de Bruijn graph in order to reconstruct genomic sequences, it appeared to be rather impractical due to the presence of complex genomic repeats and sequencing artifacts, such as errors and coverage gaps. Instead, genome assemblers implement various heuristic approaches, most of which are based on coverage depth, graph topology and the fact that the genome corresponds to one or more long paths traversing through the graph [34, 14]. Indeed, the later observation is not correct for the case of transcriptome assembly, in which RNA sequences correspond to numerous shorter path in the graph. Thus, to enable high-quality assemblies from RNA-Seq data the majority of procedures in the SPAdes pipeline have to undergo major alternations.

SPAdes pipeline for genome assembly consists of the following major steps: (i) sequencing error correction using BayesHammer module [23], (ii) construction of the condensed de Bruijn graph, (iii) graph simplification, which implies removing chimeric and erroneous edges, and (iv) repeat resolution and scaffolding with exSPAnder algorithm [35, 36]. While BayesHammer works well on the data with highly uneven coverage depth and requires no change for RNA-Seq datasets, graph simplification and repeat resolution procedures strongly rely on the properties of genomic sequences and thus require significant modifications and novel functionality for *de novo* transcriptome assembly. Below we describe the key changes introduced in rnaSPAdes.

Simplification of the de Bruijn graph in rnaSPAdes

During the graph simplification stage erroneous edges are removed from the de Bruijn graph based on various criteria in order to obtain clean graph containing only correct sequences (further referred to as an *assembly graph*). In the SPAdes pipeline the simplification process includes multiple various procedures that can be classified into three types: (i) trimming *tips* (dead-end or dead-start edges), (ii) collapsing *bulges* (alternative paths) and (iii) removing *erroneous connections* (chimeric and other false edges). In this section we present alternations introduced in rnaSPAdes simplification pipeline. We also provide comparison between initial and improved simplification procedures on several RNA-Seq datasets in the Supplementary material (Table S10).

Trimming tips

In the de Bruijn graph constructed from DNA reads the major fraction of tips (edges starting or ending at a vertex without other adjacent edges) typically correspond to sequencing errors and thus have to be removed. Since only a few tips are correct and either represent chromosome ends or formed by coverage gaps, the existing genome assemblers implement rather aggressive tip clipping procedures [34, 13] assuming that coverage gaps appear rather rarely. However, in the de Bruijn graph built from RNA-Seq data a significant amount of tips correspond to transcripts' ends and thus have to be preserved. In order to keep correct tips and obtain full-length transcripts, rnaSPAdes uses lower coverage and length thresholds for tip trimming procedure than SPAdes (see details below).

In some cases, tips originate from sequencing errors in multiple reads from highly-expressed isoforms and thus may have coverage above the threshold. While genome assemblers may also exploit relative coverage cutoff to remove such tips, in transcriptome assembly this approach may result in trimming correct tips corresponding to the ends of low-expressed isoforms. However, erroneous tips typically have a small difference from the correct sequence without errors (e.g. 1-2 mismatches). To address this issue, we align tips to the alternative (correct) edges of the graph (Fig. 4a) and trim them if the identity exceeds a certain threshold (similar procedure is implemented in truSPAdes, which was designed for True Synthetic Long Reads assembly [37]). In case when two tips correspond to the ends of an alternatively spliced isoforms, it is highly unlikely for them to have similar nucleotide sequences (Fig. 4b).

Another specifics of RNA-seq datasets is the large number of low-complexity regions that originate from poly-A tails resulting from polyadenylation at the ends of mRNAs. In order to avoid chimeric connections and non-informative sequences, we also remove low-complexity edges from the de Bruijn graph (see exact criterion below).

Below we summarize all conditions used in tip clipping procedure, parameters for which were optimized based on our analysis of various RNA-seq datasets. We define l_T as the length of the tip that is being analyzed and c_T as its mean k-mer coverage, and c_A as the k-mer coverage of the alternative edge (which is presumably correct) A tip is removed if any of the following conditions is true:

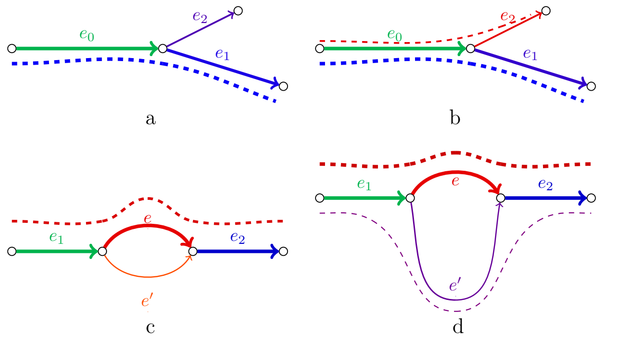

Figure 4. Examples of tips and bulges in the condensed de Bruijn graph. Edges with similar colors have similar sequences; line width represents the coverage depth. (a) Correct transcript (blue dashed line) traverses through edges e_0 and e_1. Edge e_2 is originated from the reads with the same sequencing error and thus has coverage depth high enough not to be trimmed. However, since the sequence of edge e_2 is very similar to the sequence of the alternative edge e_1 (detected by alignment), e_2 is eventually removed as erroneous. (b) In this case both paths (e_0, e_1) and (e_0, e_2) correspond to correct isoforms (blue and red dashed lines). Since the sequences of e_1 and e_2 are likely to be different, none of the correct tips is removed. (c) Correct sequence (red dashed line) traverses through edges e_1, e and e_2. Edge e' is originated from reads containing sequencing errors, and thus has sequence similar to e, but significantly lower coverage. (d) Both paths (e_1, e, e_2) and (e_1, e', e_2) correspond to different isoforms of the same gene (red and purple dashed lines); edges e and e' typically have different length, coverage depth and sequence.

- $l < 2 \cdot k$ and $c_T \leq 1$ (short tips with very low coverage);
- $l < 4 \cdot k$, $c_T < c_A/2$ and the Hamming distance between the tip and the alternative edge does not exceed 3 (the tip containing a sequencing error);
- the tip contains more than 80% of A/T nucleotides (low complexity tip).

Collapsing bulges

A simple bulge (two edges sharing starting and terminal vertices) in the de Burijn graph may correspond to one of the following events: (i) a sequencing error, (ii) a heterozygous mutation or another allele difference, or (iii) an alternative splicing event (typical for transcriptomic data). The first two cases are characterized by the bulge edges having similar lengths and sequences. However, edges formed by sequencing errors are typically short and have significantly different coverage depth, since it is unlikely for the same error to occur numerous times at the same position (Fig. 4c). Vice versa, in the case of allele difference bulge edges usually have similar coverage. Thus, genome assembly algorithms for bulge removal typically rely on the coverage depth [34, 13]. Since the most typical difference between two alternatively spliced isoforms of the same gene is the inclusion/exclusion of a an exon (usually short), edges of the bulge originated from these isoforms have different lengths (Fig. 4d). At the same time, since the expression levels may vary for such isoforms, the coverage depth may significantly differ. To avoid missing alternatively spliced isoforms in the assembly, rnaSPAdes does not use any coverage threshold for bulge removal and collapses only bulges consisting of edges with the similar lengths (less than 10% difference in length).

Removing chimeric connections

While undetected tips and bulges formed by sequencing errors result in mismatches and indels in the assembled contigs, chimeric reads (typically corresponding to a concatenation of sequences from distant regions of the original molecules) may trigger more serious errors, such as incorrect junctions in the resulting contigs (often referred to as misassemblies). In conventional genome assembly chimeric edges usually have low coverage and thus can be easily identified [34]. Single-cell datasets, however, feature multiple low-covered genomic regions and elevated number of chimeric reads, which result in numerous erroneous connections having higher coverage depth than correct genomic edges. Similarly, since true edges representing low-expressed isoforms in the transcriptome assembly also have relatively low coverage depth, cleaning the graph using coverage threshold will result in multiple missing transcripts in the assembly.

To detect chimeric connections in single-cell assemblies SPAdes implements various algorithms, which mostly rely on the assumption that each chromosome corresponds to a long contiguous path traversing through the de Bruijn graph [14]. Since this assumption does not hold for transcriptomes consisting of thousands isoforms, we had to disable most procedures for the chimeric edge detection in SPAdes and implement a new erroneous edge removal algorithm that addresses the specifics of chimeric reads in RNA-seq data sets.

Our analysis revealed that most of the chimeric connections in RNA-seq data can be divided into two groups: single-strand chimeric loops and double-strand hairpins. In the first case, a chimeric junctions connects the end of a transcript sequence with itself (Fig. 5a). The erroneous hairpin connects correct edge with its reverse-complement copy (Fig. 5b) and potentially may result in chimeric palindromic sequence in the assembly. To avoid misassemblies, rnaSPAdes detects chimeric loops and hairpins by analyzing the graph topology rather than nucleotide sequences or coverage.

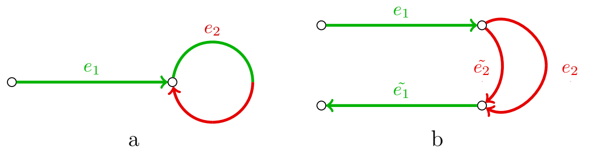

Figure 5. Examples of chimeric connections in the de Bruijn graph typical for transcriptome assembly. Red and green indicate erroneous and correct sequences respectively. (a) A chimeric loop (edge e_2) connecting end of the correct transcriptomic edge e_1 with itself. (b) An example of chimeric hairpin, where erroneous edge e_2 connects a correct edge e_1 with its reverse-complement copy $\tilde{e_1}$. Since e_2 connects a vertex and its reverse-complement, $\tilde{e_2}$ (the reverse-complement of e_2) also connects these two vertices.

While it remains unclear whether these chimeric reads are formed during transcription, RNA-seq sample preparation or sequencing, similar chimeric connections have been observed in the context of single-cell MDA. E.g., when a DNA fragment is amplified by MDA, the DNA polymerase moves along DNA molecule and copies it, but sometimes (as described in [15]), the polymerase may jump to a close position (usually on the opposite DNA strand) and proceed to copy from the new position.

Selecting optimal k-mer sizes

One of the key techniques that allows SPAdes to assemble contiguous genomic sequences from the data with non-uniform coverage depth is the iterative de Bruijn graph construction. During each next iteration, SPAdes builds the graph from the input reads and sequences obtained at the previous iteration, simplifies the graph and provides its edges as an input to the next iteration that uses larger k-mer size. Assembly graph obtained at the final iteration is used for repeat resolution and scaffolding procedures, which exploit read-pairs and long reads [35, 32]. In this approach small k-mer sizes help to assemble low-covered regions where reads have short overlaps, and large k values are useful for resolving repeats and therefore obtaining less tangled graph. Although this method seems to

be useful for restoring low-expressed isoforms from RNA-Seq data, our analysis revealed that it appears to be the main reason of the high number of misassembled contigs in SPAdes assemblies. Below we describe how these false junctions are formed.

When two transcripts (possibly from different genes) have a common sequence in the middle, they form a typical repeat structure in the de Bruijn graph (Fig. 6a), which may further be resolved, e.g. using paired reads. However, if a common sequence appears close to the ends of the transcripts (Fig. 6b), edges e_2 and e_3 appear to be rather short and may be trimmed as tips (since coverage depth often drops near the transcripts ends), or may not be present at all. In this case, the remaining edges e_1, e and e_4 will be condensed into a single edge corresponding to chimeric sequence.

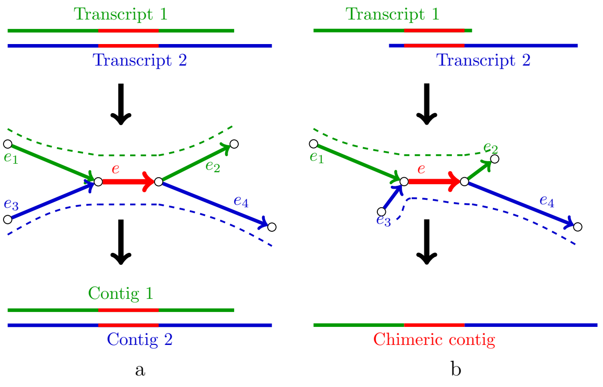

Figure 6. Examples of two transcripts having a common sequence (a) in the middle of the transcripts and (b) close to the start of one transcript and the end of another. While in the first case the isoforms can be resolved using read-pairs, the latter one may potentially result in a chimeric contig.

Indeed, since small k-mer size results in a higher chance of creating such kind of chimeric junction, we decided to modify the parameters for the iterative graph construction. In rnaSPAdes we decided to use only two k values: smaller one for restoring low-covered regions with insufficient overlaps between reads and larger one for obtaining less tangled graph.

To estimate the optimal k values, we ran rnaSPAdes on several RNA-Seq datasets with various read lengths sequenced from organisms with different gene complexity. Since it requires tremendous amount of time to try all possible pairs of k-mer sizes on multiple datasets, we first estimated upper k value used for the main iteration, and then selected lower k with the fixed upper one.

We assembled a number of datasets using only a single k-mer size and selected the best assemblies according to number of assembled genes, database coverage and number of misassemblies. Although it may be not possible to choose a single best k value simultaneously for multiple datasets, nearly optimal k-mer size was estimated as half of the read length (more precisely, the largest odd number that does not exceed $read\_length/2 - 1$). The smaller k value was estimated in a similar manner with the fixed upper k-mer size. Optimal lower k was considered based on number of additional assembled genes and misassemblies. Experiments showed that small k values (e.g. below 29) tend to dramatically increase the number of erroneous contigs due to the higher probability of two transcripts sharing the same k-mer. Thus, the lower k-mer size was estimated approximately as $read\_length/3$ with the minimal possible value set to 29. Although estimated k values may not provide the best assembly for every dataset, they typically appear to be a good trade-off between the number of recovered genes and generated errors (see Supplementary Tables S7-S9).

In this work rnaSPAdes was launched with the default k values. Indeed, rnaSPAdes keeps the possibility to set the k-mer sizes manually. In addition, we introduced the `--fast` option, which forces the assembler to use only a single k value (the optimal upper k). Typically, assemblies obtained with a single k capture fewer genes and isoforms (especially low-covered), but also have smaller number of misassembled contigs (see Supplementary material for comparison).

In order to preserve correct connections that could be restored using only small k-mer sizes, we carefully examined low-expressed transcripts that were not completely assembled using default k-mer sizes. The analysis revealed that the majority of such fragments can be joined by the small overlap, which is often confirmed by the read-pairs. To perform the gap closing procedure rnaSPAdes glues two tips if one of the following conditions is true:

- tips have an exact overlap of length at least L_{ov} and are connected by at least N_{ov} read pairs;
- tips are connected by at least N_{min} read pairs.

where the default parameters are $L_{ov} = 8$ bp, $N_{ov} = 1$ and $N_{min} = 5$. Although these parameters seem to be slightly ad-hoc, such gap closing procedure appears to be a viable alternative to using small k values and allows to restore more low-expressed transcripts without increasing the number of misassemblies. Using smaller thresholds for gap closing often create false connections and increase the amount of erroneous transcripts, while larger values for these parameters result in a smaller increase of reconstructed sequences.

Isoform reconstruction

Adapting repeat resolution algorithms

Genomic repeats present one of the key challenges in the *de novo* genome assembly problem. Although mRNA sequences typically do not contain complex repeats, transcriptome assembly has a somewhat similar problem of resolving alternatively spliced isoforms and transcripts from paralogous genes. Repeat resolution and scaffolding steps in SPAdes genome assembler are implemented in the exSPAnder module [35], which is based on simple path-extension framework. Similar to other modules of SPAdes, exSPAnder was designed to deal with highly uneven coverage and thus can be adapted for isoform detection procedure when assembling RNA-Seq data.

The key idea of the path-extension framework is to iteratively construct paths in the assembly graph by selecting the best-supported extension edge at every step until no extension can be chosen. The extension is selected based on the scoring function that may exploit various kinds of linkage information between edges of the assembly graph (different scoring functions are implemented for different types of sequencing data). A situation when a path cannot be extended further is usually caused by the presence of long genomic repeat or a large coverage gap. The extension procedure starts from the longest edge that is not yet included in any path and is repeated until all edges are covered.

More formally, a path extension step can be defined as follows. For a path P and its extension edges $e_1, \ldots, e_n$ (typically, edges that start at the terminal vertex of P) the procedure selects e_i as a best-supported extension if

i. $Score_P(e_i) > C \cdot Score_P(e_j)$ for all $j \neq i$
ii. $Score_P(e_i) > \Theta$

where C and Θ are the algorithm parameters, and $Score_P(e_i)$ is

a score of edge e_i relative to path P (described in [35]).

In contrast to genome assembly, in which there is usually only one true extension edge, in transcriptome assembly multiple correct extensions are possible due to the presence of alternatively spliced isoforms. Thus, the modified procedure is capable of selecting several edges $e_{k_1}, \ldots e_{k_m}$ among all possible extensions $e_1, \ldots, e_n$, which satisfy the following conditions:

i. $Score_P(e_{k_i}) > Score_P(e_M)/C$ for all $i = 1 \ldots m$,
$M = argmax_{j=1..n} Score_P(e_j)$
ii. $Score_P(e_{k_i}) > \Theta$ for all $i = 1 \ldots m$

Namely, all correct extension edges must have a score close to the maximal one ($C = 1.5$ by default), and the second condition remains the same. Afterwards, the algorithm extends path P by creating new paths $(P, e_{k_1}), \ldots, (P, e_{k_m})$, which are then extended independently. Since the scoring function implemented in exSPAnder does not strongly depend on the coverage depth, there is no danger that highly-expressed isoforms will be preferred over the low-expressed ones.

Finally, to avoid duplications in the genome assemblies, exSPAnder performs rather aggressive overlap removal procedure. However, since alternatively spliced isoforms may differ only by a short exon, in order to avoid missing similar transcripts the modified overlap detection procedure removes only exact duplicates and sub-paths.

Exploiting coverage depth

Varying coverage depth may seem to be an additional challenge for *de novo* sequence assembly, but can be also used as an advantage in some cases. For instance, if two alternatively spliced isoforms of the same gene have different expression levels, they can be resolved using coverage depth even when the read-pairs do not help (e.g. shared exon is longer than the insert size). Although using coverage values becomes more complicated when a gene has multiple different expressing isoforms, our analysis of several RNA-Seq datasets revealed that such cases are rather rare and most of the genes have one or two expressing isoforms within a single sample.

To exploit the coverage depth we decided to add a simple, but reliable path-extension rule. Let the path $P = (e_1, e_2, e_3)$ have extension edges e and e' (Fig. 7a), such that $cov(e) > cov(e')$ and $cov(e_2) > cov(e'_2)$, where $cov(e)$ denotes the k-mer coverage of edge e. To select a correct extension the algorithm detects a vertex closest to the end of path P that has two incoming alternative edges, one of which is included in P and another is not (e_2 and e'_2 in this example). Since edge $e_2 \in P$ has higher coverage than the alternative edge $e'_2 \notin P$, we select extension edge e as the one with the higher coverage. However, if both isoforms have similar coverage, this simple approach may chose a false extension (since the coverage depth is rarely perfectly uniform even along a small region). Thus the difference in coverage should be significant enough to distinguish between the isoforms. More formally, the following conditions should be satisfied:

i. $cov(e) > \Delta \cdot cov(e')$
ii. $cov(e_2) > \Delta \cdot cov(e'_2)$
iii. $\Delta > cov(e_2)/cov(e) > 1/\Delta$
iv. $cov(e) > C_{min}$

where the default values of the algorithm parameters are $\Delta = 2$ and $C_{min} = 2$. The first two conditions ensure that the extension edges (e and e') and alternative edges (e_2 and e'_2) have significant coverage difference, the third one requires the coverage depth to remain relatively persistent along the path and the latter one prevents the algorithm from resolving low-covered isoforms (which may result in a misassembly). In general case, this procedure also utilizes only the last pair of alternative edges, and is applied only in case when the path has two possible extension edges and conventional read-pair extender fails to extend the path.

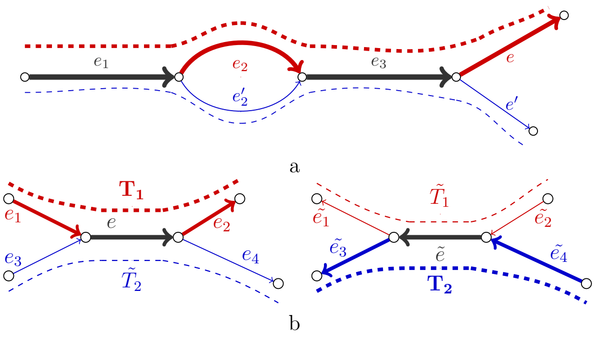

Figure 7. Using coverage depth for isoform reconstruction. Line width represents conventional and strand-specific coverage depths in figures (a) and (b) respectively. (a) Two isoforms of the same gene (red and blue dashed lines) have different expression levels and thus can be resolved using coverage depth. (b) Two transcripts T_1 and T_2 (red and blue bold dashed lines respectively) share a reverse-complement sequence and thus can be resolved using strand-specific reads.

Assembling strand-specific data

Another possible way to improve a transcriptome assembly is to take the benefit of strand-specific data when provided. To utilize stranded RNA-Seq we introduce *strand-specific coverage depths* $cov^+(e)$ and $cov^-(e)$, which denote k-mer coverage of edge e by forward and reverse reads respectively. As opposed to the conventional coverage $cov(e)$, which is calculated by aligning all reads and their reverse-complement copies to the edges of the assembly graph (thus making $cov(e) = cov(\tilde{e})$), strand-specific coverage is obtained by mapping reads according to their origin strand. For instance, if an RNA-Seq library is constructed in such way that reads have the same strand as the transcript which they were sequenced from, we expect $cov^+(e)$ to be much higher than $cov^-(e)$ if the sequence of e corresponds to the transcript, and vice versa if e is the reverse-complement of the original transcript. Indeed, the situation becomes opposite when reads are sequenced from cDNAs that are reverse-complement to the original transcripts. When working with paired-end libraries, we assume that the type of the library is defined by the first read's strand. For example, if paired-end reads have common forward-reverse orientation, the second read in pair is reverse-complemented before mapping in order to match the strand of the first read.

To extend the paths we apply the same path-extension procedure described above for conventional coverage, but use strand-specific coverage values instead. Fig. 7b demonstrates a situation, when two transcript correspond to paths $T_1 = (e_1, e, e_2)$ and $T_2 = (\tilde{e_4}, \tilde{e}, \tilde{e_3})$. If the repetitive edge e is longer than the insert size and the conventional coverage depth of these two transcripts is similar, the situation cannot be resolve neither by paired reads, nor by coverage. However, in case of stranded data, strand-specific coverage for actual transcripts' paths will be much higher than for their reverse-complement copies, i.e. $cov^+(T_1) >> cov^+(\tilde{T_1})$ and $cov^+(T_2) >> cov^+(\tilde{T_2})$ (in this example we assume that reads have the same stand as the transcripts they come from). Moreover, edges corresponding to the reverse-complement sequences only ($\tilde{e_1}$ and $\tilde{e_2}$ for $\tilde{T_1}$, e_3 and e_4 for $\tilde{T_2}$) will have $cov^+(e)$ values close to zero. Therefore, the conditions given for coverage-based path extender (see previous subsection) will be satisfied for strand-specific coverage values, the repetitive edge e will be resolved and both

transcripts will be reconstructed.

In addition, for stranded RNA-Seq data we output the paths constructed by the exSPAnder algorithm according to the original transcript's strand. E.g. in the example given in Figure 7b rnaSPAdes will output paths T_1 and T_2, since they have higher strand-specific coverage than their reverse complement copies ($\tilde{T_1}$ and $\tilde{T_2}$ respectively).

Filtering assembled transcripts

Before outputting the paths constructed by the exSPAnder module as contigs, we additionally apply various filtering procedures in order to remove non-mRNA contigs, such as intergenic sequences, which often contaminate RNA-Seq datasets. Our analysis showed that the majority of such unwanted sequences have low coverage, relatively small length and often correspond to isolated edges in the assembly graph (i.e. have no adjacent edges). However, applying filters based on these criteria may also remove correct low-expressed transcripts in some cases. Thus, we decided to implement three different presets of parameters for the filtration procedure (soft, normal and hard) and output three files with contigs. Depending on the project goal the researcher may choose more sensitive (soft filtration) or more specific results (hard filtration). Table S11 in the Supplementary material shows how the assembly quality depends on the filtration parameters. In other tables we use default transcripts with the normal level of filtering.

Availability of source code and requirements

rnaSPAdes is implemented in C++ and Python and is freely available for Linux and MacOS under GPLv2 license at cab.spbu.ru/software/rnaspades/ and github.com/ablab/spades.

Availability of supporting data and materials

All real RNA-Seq datasets are available at short read archive (https://www.ncbi.nlm.nih.gov/sra) with the following accession numbers

- *H. sapiens*: SRR5133163
- *M. musculus*: SRX648736
- *C. elegans*: SRR1560107
- *Z. mays*: SRR1588569

Simulated data is available on the server

- *H. sapiens*: http://spades.bioinf.spbau.ru/rnaspades/simulated_data/human/
- *M. musculus*: http://spades.bioinf.spbau.ru/rnaspades/simulated_data/mouse/

Declarations

Consent for publication

Not applicable.

Competing Interests

The authors declare that they have no competing interests.

Funding

The work was supported by Russian Science Foundation (grant 14-50-00069).

Author's Contributions

Software design and implementation was performed by EB, DA and AP. EB was responsible for data curation, assemblers benchmarking and manuscript editing. AL supervised the project and performed funding acquisition. AP wrote the manuscript and managed the project. All authors read and approved the final manuscript.

Acknowledgements

Not applicable.

References

1. Li B, Dewey CN. RSEM: accurate transcript quantification from RNA-Seq data with or without a reference genome. BMC bioinformatics 2011;12(1):323.
2. Trapnell C, et al. Differential gene and transcript expression analysis of RNA-Seq experiments with TopHat and Cufflinks. Nature protocols 2012;7(3):562.
3. Dobin A, et al. STAR: ultrafast universal RNA-Seq aligner. Bioinformatics 2013;29(1):15–21.
4. Kim D, et al. TopHat2: accurate alignment of transcriptomes in the presence of insertions, deletions and gene fusions. Genome Biol 2013;14(4):R36.
5. Love MI, Huber W, Anders S. Moderated estimation of fold change and dispersion for RNA-seq data with DESeq2. Genome biology 2014;15(12):550.
6. Pertea M, et al. StringTie enables improved reconstruction of a transcriptome from RNA-seq reads. Nature biotechnology 2015;33(3):290.
7. Robertson G, et al. De novo assembly and analysis of RNA-seq data. Nat Methods 2010;7(11):909–912.
8. Grabherr MG, et al. Full-length transcriptome assembly from RNA-Seq data without a reference genome. Nat Biotechnol 2011 Jul;29(7):644–652.
9. Schulz MH, et al. Oases: robust de novo RNA-seq assembly across the dynamic range of expression levels. Bioinformatics 2012;28(8):1086–1092.
10. Peng Y, et al. IDBA-tran: a more robust de novo de Bruijn graph assembler for transcriptomes with uneven expression levels. Bioinformatics 2013;29(13):i326–i334.
11. Xie Y, et al. SOAPdenovo-Trans: De novo transcriptome assembly with short RNA-Seq reads. Bioinformatics 2014;p. btu077.
12. Martin JA, Wang Z. Next-generation transcriptome assembly. Nat Rev Genet 2011 Oct;12(10):671–682.
13. Bankevich A, et al. SPAdes: A New Genome Assembly Algorithm and Its Applications to Single-Cell Sequencing. Journal of Computational Biology 2012;19:455–477. http://dx.doi.org/10.1089/cmb.2012.0021.
14. Nurk S, et al. Assembling Single-Cell Genomes and Mini-Metagenomes From Chimeric MDA Products. Journal of Computational Biology 2013;20:1–24.
15. Lasken RS. Single-cell genomic sequencing using Multiple Displacement Amplification. Current opinion in microbiology 2007 Oct;10:510–516. http://dx.doi.org/10.1016/j.mib.2007.08.005.
16. Bushmanova E, et al. rnaQUAST: a quality assessment

tool for de novo transcriptome assemblies. Bioinformatics 2016;32(14):2210–2212.
17. Liu J, Li G, Chang Z, Yu T, Liu B, McMullen R, et al. BinPacker: packing-based de novo transcriptome assembly from RNA-seq data. PLoS computational biology 2016;12(2):e1004772.
18. Chang Z, Li G, Liu J, Zhang Y, Ashby C, Liu D, et al. Bridger: a new framework for de novo transcriptome assembly using RNA-seq data. Genome biology 2015;16(1):30.
19. Nip KM. RNA-Bloom: de novo RNA-seq assembly with Bloom filters. PhD thesis, University of British Columbia; 2017.
20. Smith-Unna R, et al. TransRate: reference-free quality assessment of de novo transcriptome assemblies. Genome research 2016;2(8):1134–1144.
21. Simão FA, et al. BUSCO: assessing genome assembly and annotation completeness with single-copy orthologs. Bioinformatics 2015;31(19):3210–3212.
22. Li B, et al. Evaluation of de novo transcriptome assemblies from RNA-Seq data. Genome Biol 2014;15(12):553.
23. Nikolenko SI, Korobeynikov AI, Alekseyev MA. BayesHammer: Bayesian clustering for error correction in single-cell sequencing. BMC Genomics 2013;14.
24. Leung HC, Yiu SM, Parkinson J, Chin FY. IDBA-MT: de novo assembler for metatranscriptomic data generated from next-generation sequencing technology. Journal of Computational Biology 2013;20(7):540–550.
25. Leung HC, Yiu SM, Chin FY. IDBA-MTP: a hybrid Metatranscriptomic assembler based on protein information. Journal of Computational Biology 2015;22(5):367–376.
26. Garalde DR, Snell EA, Jachimowicz D, Sipos B, Lloyd JH, Bruce M, et al. Highly parallel direct RNA sequencing on an array of nanopores. Nature methods 2018;15(3):201.
27. Tilgner H, Grubert F, Sharon D, Snyder MP. Defining a personal, allele-specific, and single-molecule long-read transcriptome. Proceedings of the National Academy of Sciences 2014;p. 201400447.
28. Byrne A, Beaudin AE, Olsen HE, Jain M, Cole C, Palmer T, et al. Nanopore long-read RNAseq reveals widespread transcriptional variation among the surface receptors of individual B cells. Nature Communications 2017;8:16027.
29. Minoche AE, Dohm JC, Schneider J, Holtgräwe D, Viehöver P, Montfort M, et al. Exploiting single-molecule transcript sequencing for eukaryotic gene prediction. Genome biology 2015;16(1):184.
30. Abdel-Ghany SE, Hamilton M, Jacobi JL, Ngam P, Devitt N, Schilkey F, et al. A survey of the sorghum transcriptome using single-molecule long reads. Nature communications 2016;7:11706.
31. Gordon SP, Tseng E, Salamov A, Zhang J, Meng X, Zhao Z, et al. Widespread polycistronic transcripts in fungi revealed by single-molecule mRNA sequencing. PloS one 2015;10(7):e0132628.
32. Antipov D, et al. hybridSPAdes: an algorithm for hybrid assembly of short and long reads. Bioinformatics 2015;32(7):1009–1015.
33. Pevzner PA, et al. An Eulerian path approach to DNA fragment assembly. Proceedings of the National Academy of Sciences 2001;98:9748–9753. http://dx.doi.org/10.1073/pnas.171285098.
34. Zerbino DR, Birney E. Velvet: Algorithms for de novo short read assembly using de Bruijn graphs. Genome Research 2008;18:821–829. http://dx.doi.org/10.1101/gr.074492.107.
35. Prjibelski AD, et al. ExSPAnder: a universal repeat resolver for DNA fragment assembly. Bioinformatics 2014;30(12):i293–i301.
36. Vasilinetc I, et al. Assembling short reads from jumping libraries with large insert sizes. Bioinformatics 2015;31(20):3262–3268.
37. Bankevich A, Pevzner PA. TruSPAdes: barcode assembly of TruSeq synthetic long reads. Nature methods 2016;13(3):248.

Click here to access/download
Supplementary Material
rnaSPAdes_supplement.pdf
