# Supplementary material for: rnaSPAdes: a de novo transcriptome assembler and its application to RNA-Seq data
Source: Gigascience. 2019 Sep 3;8(9):giz100. doi: 10.1093/gigascience/giz100 (PMC6736328; doi:10.1093/gigascience/giz100)
Supplement: giz100_GIGA-D-18-00456_Revision_1 [file giz100_giga-d-18-00456_revision_1.pdf]

GigaScience

rnaSPAdes: a de novo transcriptome assembler and its application to RNA-Seq data

--Manuscript Draft--

**Manuscript Number:**	GIGA-D-18-00456R1
**Full Title:**	rnaSPAdes: a de novo transcriptome assembler and its application to RNA-Seq data
**Article Type:**	Research
**Funding Information:**	Russian Foundation for Basic Research (19-04-01074) — Mr Andrey D. Prjibelski<br>St. Petersburg State University (15.61.951.2015) — Not applicable
**Abstract:**	Possibility to generate large RNA-Seq datasets has led to development of various reference-based and de novo transcriptome assemblers with their own strengths and limitations. While reference-based tools are widely used in various transcriptomic studies, their application is limited to the organisms with finished and well-annotated genomes. De novo transcriptome reconstruction from short reads remains an open challenging problem, which is complicated by the varying expression levels across different genes, alternative splicing and paralogous genes. In this paper we describe a novel transcriptome assembler called rnaSPAdes, which is developed on top of SPAdes genome assembler and explores computational parallels between assembly of transcriptomes and single-cell genomes. We also present quality assessment reports for rnaSPAdes assemblies, compare it with modern transcriptome assembly tools using several evaluation approaches on various RNA-Seq datasets, and briefly highlight strong and weak points of different assemblers.
**Corresponding Author:**	Andrey D. Prjibelski, M.Sc.<br>SPbU<br>St. Petersburg, Russia RUSSIAN FEDERATION
**Corresponding Author Secondary Information:**	
**Corresponding Author's Institution:**	SPbU
**Corresponding Author's Secondary Institution:**	
**First Author:**	Elena Bushmanova, M.Sc.
**First Author Secondary Information:**	
**Order of Authors:**	Elena Bushmanova, M.Sc.<br>Dmitry Antipov, M.Sc.<br>Alla Lapidus, Ph. D.<br>Andrey D. Prjibelski, M.Sc.
**Order of Authors Secondary Information:**	
**Response to Reviewers:**	Attached as PDF
**Additional Information:**	
**Question**	**Response**
Are you submitting this manuscript to a special series or article collection?	No
**Experimental design and statistics**<br><br>Full details of the experimental design and statistical methods used should be given	Yes

Powered by Editorial Manager® and ProduXion Manager® from Aries Systems Corporation

in the Methods section, as detailed in our Minimum Standards Reporting Checklist. Information essential to interpreting the data presented should be made available in the figure legends.<br><br>Have you included all the information requested in your manuscript?	
**Resources**<br><br>A description of all resources used, including antibodies, cell lines, animals and software tools, with enough information to allow them to be uniquely identified, should be included in the Methods section. Authors are strongly encouraged to cite Research Resource Identifiers (RRIDs) for antibodies, model organisms and tools, where possible.<br><br>Have you included the information requested as detailed in our Minimum Standards Reporting Checklist?	Yes
**Availability of data and materials**<br><br>All datasets and code on which the conclusions of the paper rely must be either included in your submission or deposited in publicly available repositories (where available and ethically appropriate), referencing such data using a unique identifier in the references and in the "Availability of Data and Materials" section of your manuscript.<br><br>Have you have met the above requirement as detailed in our Minimum Standards Reporting Checklist?	Yes

Main manuscript

Click here to access/download;Manuscript;Bushmanova.pdf

Click here to view linked References

GigaScience, 2019, 1–12

doi: xx.xxxx/xxxx
Manuscript in Preparation
Paper

PAPER

rnaSPAdes: a *de novo* transcriptome assembler and its application to RNA-Seq data

Elena Bushmanova[1], Dmitry Antipov[1], Alla Lapidus[1] and Andrey D. Prjibelski[1*]

[1]Center for Algorithmic Biotechnology, Institute of Translational Biomedicine, St. Petersburg State University, St. Petersburg, Russia

[*]To whom correspondence should be addressed: a.przhibelsky@spbu.ru

Abstract

Possibility to generate large RNA-Seq datasets has led to development of various reference-based and *de novo* transcriptome assemblers with their own strengths and limitations. While reference-based tools are widely used in various transcriptomic studies, their application is limited to the organisms with finished and well-annotated genomes. *De novo* transcriptome reconstruction from short reads remains an open challenging problem, which is complicated by the varying expression levels across different genes, alternative splicing and paralogous genes. In this paper we describe a novel transcriptome assembler called rnaSPAdes, which is developed on top of SPAdes genome assembler and explores computational parallels between assembly of transcriptomes and single-cell genomes. We also present quality assessment reports for rnaSPAdes assemblies, compare it with modern transcriptome assembly tools using several evaluation approaches on various RNA-Seq datasets, and briefly highlight strong and weak points of different assemblers.

Key words: RNA-Seq; *de novo* assembly; transcriptome assembly

Background

While reference-based methods for RNA-Seq analysis [1, 2, 3, 4, 5, 6] are widely used in transcriptome studies, they are subjected to the following constraints: (i) they are not applicable in the case when the genome is unknown, (ii) their performance deteriorates when the genome sequence or annotation are incomplete, and (iii) they may miss unusual transcripts such as fusion genes or genes with short unannotated exons. To address these constraints, *de novo* transcriptome assemblers [7, 8, 9, 10, 11] have emerged as a viable complement to the reference-based tools. Although *de novo* assemblers typically generate fewer complete transcripts than the reference-based methods for the organisms with accurate reference sequences [12], they may provide additional insights on aberrant transcripts.

While the transcriptome assembly may seem to be a simpler problem than the genome assembly, RNA-Seq assemblers have to address the complications arising from highly uneven read coverage depth caused by variations in gene expression levels. However, this is the same challenge that we have addressed while developing SPAdes assembler [13, 14], which originally aimed at single-cell sequencing. Similarly to RNA-Seq, the Multiple Displacement Amplification (MDA) technique [15], used for genome amplification of single bacterial cells, results in a highly uneven read coverage. In the view of similarities between RNA-seq and single-cell genome assemblies, we decided to test SPAdes without any modifications on transcriptomic data. Even though SPAdes is a genome assembler and was not optimized for RNA-seq data, in some cases it generated decent assemblies of quality comparable to the state-of-the-art transcriptome assemblers.

To perform the benchmarking we have used rnaQUAST tool [16], which was designed for quality evaluation of *de novo* assemblies with the support of reference genome and its gene database. For the comparison, we selected a few representative metrics such as (i) total number of assembled transcripts (contigs), (ii) reference gene database coverage, (iii) number of

Compiled on: April 20, 2019.
Draft manuscript prepared by the author.

Table 1. Benchmarking of **BinPacker**, **Bridger**, **IDBA**-tran, **RNA-Bloom**, **SOAP**denovo-Trans, **SPAdes**, Trans-**ABySS** and **Trinity** on Mouse RNA-seq dataset (accession number SRX648736). The annotated transcriptome of *M. musculus* GRCm38.75 consists of 38924 genes and 94545 isoforms. All contigs shorter than 200 bp were filtered out prior to the analysis. The best values for each metric are highlighted with bold.

	BinPacker	Bridger	IDBA	Bloom	SOAP	SPAdes	ABySS	Trinity
Transcripts	27234	42029	38313	46440	31878	42949	36488	47746
Misassemblies	947	923	387	732	**37**	497	194	459
Duplication ratio	1.12	1.09	**1.00**	1.33	**1.00**	**1.00**	1.09	1.15
Database coverage, %	14.4	16.3	16.9	13.8	15.1	17.7	16.2	**18.2**
50%-assembled genes	6005	6090	6558	4859	6241	**6890**	6321	6633
95%-assembled genes	1917	1909	1602	1256	1653	**2450**	1798	2272
50%-assembled isoforms	6360	6451	6790	5591	6376	7053	6931	**7386**
95%-assembled isoforms	1992	1982	1602	1346	1655	**2450**	1850	2406

50% / 95%-assembled genes/isoforms, (iv) number of misassemblies and (v) duplication ratio. The detailed description for these metrics can be found in the Supplementary material.

Table 1 demonstrates comparison between different assembly tools on publicly available Mouse RNA-Seq dataset. All transcriptome assemblers were launched with default parameters, SPAdes was run in single-cell mode due to the uneven coverage depth of RNA-Seq data. Table 1 shows that SPAdes generates more 50% / 95%-assembled genes than any other tool and has comparable gene database coverage. At the same time, SPAdes produces rather high number of misassembled transcripts, which can be explained by the fact that algorithms for genome assembly tend to assemble longer contigs and may incorrectly join sequences corresponding to different genes when working with RNA-Seq data. In addition, SPAdes generates the same number of 95%-assembled genes and isoforms, which emphasizes the lack of isoform detection step.

Benchmarking on other datasets also showed that SPAdes successfully deals with non-uniform coverage depth and produces relatively high number of 50% / 95%-assembled genes in most cases. However, it also confirmed the problem of large amount of erroneous transcripts as well as relatively low number of fully reconstructed alternative isoforms in SPAdes assemblies. Based on the obtained statistics we decided to adapt current SPAdes algorithms for RNA-Seq data with the goal to improve quality of generated assemblies and develop a new transcriptomic assembler called rnaSPAdes. In this manuscript we describe major pipeline modifications as well as several algorithmic improvements introduced in rnaSPAdes that allow to avoid misassemblies and obtain sequences of alternatively spliced isoforms.

To perform sufficient benchmarking of rnaSPAdes and other transcriptome assemblers mentioned above, we assembled several simulated and publicly available real RNA-Seq datasets from the organisms with various splicing complexity. For the generated assemblies we present quality assessment reports obtained with different *de novo* and reference-based evaluation approaches. In addition, based on these results we discuss strengths and disadvantages of various assembly tools and provide insights on their performance.

Data Description

To compare rnaSPAdes performance with the state-of-the-art transcriptome assemblers we selected 2 simulated and 6 real publicly available RNA-Seq datasets (Table 2) with different (i) read length, (ii) library size, (iii) strand-specificity and (iv) organism splicing complexity. Simulated data was generated using RSEM simulator [1] based on real Human and Mouse datasets used in this study (the exact commands are provided in the Supplementary material section S3).

All downloaded public datasets were analysed using FastQC [17]. The reports showed that no dataset contains adapters or overrepresented sequences. Human large dataset was quality-trimmed using Trimmomatic [18] due to quality drop towards reads ends. All other datasets were assembled without additional preprocessing. Although 8 datasets used in this manuscript may not represent all kinds of transcriptomic data, they are sufficient for comparing different assembly tools and detecting their strengths and disadvantages.

Analyses

Selected datasets were assembled with BinPacker [19], Bridger [20], IDBA-tran [10], RNA-Bloom [21], SOAPdenovo-Trans [11], Trans-ABySS [7], Trinity [8] and rnaSPAdes using default parameters, and SPAdes [13] in single-cell mode. For a fair comparison the same minimal contig length cutoff was used for all tools (200 bp). For assemblers that have no such option, sequences shorter than 200 bp were filtered out manually. To evaluate the resulting assemblies we used rnaQUAST [16], Tran-

Table 2. RNA-Seq datasets selected for comparison of different assembly tools. All datasets contain paired-end Illumina reads; **# reads** stands for number of read pairs.

Dataset name	Organism	Tissue	# reads	Strand-specific	Read length	Insert size	Accession #
Human	*H. sapiens*	Prostate cancer cells	30 M	No	150 bp	344 bp	SRR5133163
Human large	*H. sapiens*	Blood	125 M	No	100 bp	176 bp	SRR1957703, SRR1957706
Mouse	*M. musculus*	Pancreatic islets	11 M	No	101 bp	173 bp	SRX648736
Worm	*C. elegans*	-	45 M	No	90 bp	186 bp	SRR1560107
Corn SS	*Z. mays*	Endosperm	35 M	RF	100 bp	242 bp	SRR1588569
Arabidopsis SS	*A. thaliana*	-	118 M	RF	130 bp	245 bp	SRR5344669, SRR5344670
Human simulated	*H. sapiens*	-	30 M	No	150 bp	340 bp	-
Mouse simulated	*M. musculus*	-	11 M	No	101 bp	170 bp	-

Table 3. Benchmarking of **BinPacker**, **Bridger**, **IDBA**-tran, RNA-**Bloom**, **rnaSPAdes**, **SOAP**denovo-Trans, **SPAdes**, Trans-**ABySS** and **Trinity** on Human simulated RNA-seq dataset. The annotated transcriptome of *H. sapiens* GRCh37.p13 consists of 57820 genes and 196520 isoforms. All contigs shorter than 200 bp were filtered out prior to the analysis. The best values for each metric are highlighted with bold.

	BinPacker	Bridger	IDBA	Bloom	rnaSPAdes	SOAP	SPAdes	ABySS	Trinity
Transcripts	76736	52151	58466	65968	37730	35096	42264	67511	62831
Misassemblies	7919	3512	174	358	309	198	443	**126**	1554
Duplication ratio	2.19	1.38	1.01	1.93	1.26	1.08	**1.00**	1.24	1.74
Database coverage, %	20.9	18.5	21.4	**24.6**	23.2	19.4	20.5	23.1	24.4
50%-assembled genes	11828	11476	13175	12869	**14075**	12610	13569	12740	13289
95%-assembled genes	7320	6417	2729	8910	**10934**	7685	8526	7225	9049
50%-assembled isoforms	17415	15423	18181	**21035**	19531	15638	16437	19250	20965
95%-assembled isoforms	9091	7298	2744	12108	**13387**	8151	8638	7662	12301

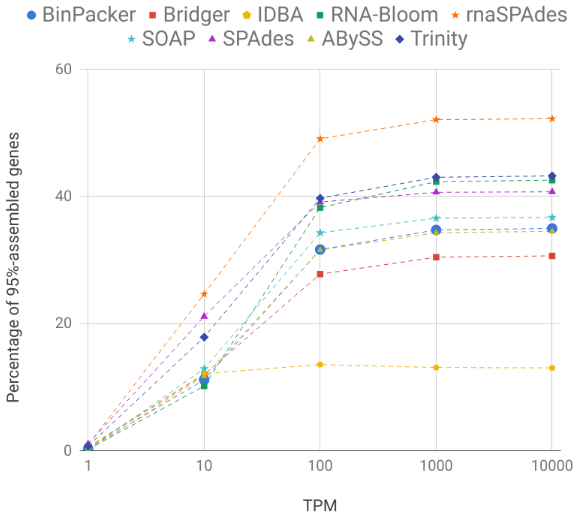

Figure 1. Cumulative plot showing how fraction of 95%-assembled genes in each assembly of Human simulated dataset depends on the gene coverage by reads in TPM (Transcripts Per Kilobase Million) reported by RSEM simulator.

srate [22], BUSCO [23] and DETONATE [24]. From each quality report we selected a few representative metrics that would allow to perform complete comparison of different assemblers. To make the results reproducible, we also provide software versions and command lines used in this study in the Supplementary material (section S3).

In addition to statistics provided by different tools, we decided to compute faction of 95%-assembled genes relative to the number of genes detected by a reference-based method. For this purpose we used genes assembled by kallisto [25] that have nucleotide coverage > 5. Coverage values were computed using estimated fragment counts. While it remains unclear how to select a proper coverage threshold for this experiment, number of genes/isoforms with coverage > 5 appeared to be the best upper bound estimate for most of the datasets (see Supplementary Table S15 for details). Using fraction of assembled genes instead of raw numbers allows to conveniently visualize the data in the same plot, compute average values across all datasets and, at the same time, estimate how well *de novo* assemblers perform relatively to the reference-based tool.

Evaluating assemblers on simulated data

To simulate RNA-Seq dataset we used RSEM simulator [1], which allows to generate reads based on the real RNA-Seq data. For this purpose we selected Human and Mouse datasets (Table 2). Table 3 shows short quality assessment report for Human simulated data. Complete evaluation reports for both simulated dataset are presented in the Supplementary material (Tables S1 and S2).

Table 3 shows that rnaSPAdes produces the highest number of 95%-assembled genes and isoforms, with Trinity and RNA-Bloom being the closest competitors. Trinity and RNA-Bloom also have the highest gene database coverage, while rnaSPAdes and Trans-ABySS are just slightly behind (1.5% difference at most). However, both Trinity and RNA-Bloom seem to produce a lot of excessive sequences resulting in high duplication ratios (1.74 and 1.93 respectively), and Trinity also appears to be somewhat inaccurate in terms of misassembled sequences (5 times more than rnaSPAdes). Among the tools with high number of assembled genes and isoforms, Trans-ABySS and SOAPdenovo-Trans are the most accurate (126 and 198 misassemblies respectively), rnaSPAdes and RNA-Bloom follow with 309 and 358 of misassembled contigs respectively. Although IDBA also generates an accurate assembly (174 misassemblies), it appears to be fragmented (small number of 95%-assembled genes and isoforms). Although both BinPacker and Bridger produce comparable amount of assembled genes and isoforms, they have the largest number of misassemblies (> 3500). BinPacker also has the highest duplication ratio (2.19).

Since RSEM simulator provides read count for each particular gene, we also computed the number of assembled genes reported by rnaQUAST depending on their read coverage (Fig. 1). The figure demonstrates that rnaSPAdes, SPAdes and Trinity outperform other tools on low-abundant transcripts, with rnaSPAdes reaching the highest fraction of total 95%-assembled genes (52.2%).

Evaluating assemblers on real RNA-Seq data

For comparison on real RNA-Seq reads we selected 4 non-stranded and 2 strand-specific datasets (Table 2). Short report for Human assemblies is shown in Table 4, while complete reports for all data are presented in the Supplementary material (Tables S3-S8 respectively). In addition, we added BUSCO reports (Supplementary Figure S2) and presented various metrics as bar plots (Figure 2, Supplementary Figures S3-S5).

Table 4 indicates, that while all assemblies have more than ten thousand of 50%-assembled genes, amount of 95%-assembled genes significantly differs. RnaSPAdes, RNA-Bloom and Trinity are the best according to 95%-assembled genes and isoforms. Among these three assemblers, rnaSPAdes dominates with 16% and 31% more 95%-assembled genes than RNA-Bloom and Trinity respectively. Although both RNA-Bloom and Trinity have the highest database coverage, they also have very high duplication ratio (≥ 2). In addition, Trinity (along with BinPacker and Bridger) generate significant amount of misassemblies (> 5000). SOAPdenovo-Trans and Trans-ABySS produce accurate assemblies according to these parameters, but generate 2043 and 2250 fewer 95%-assembled genes than rnaSPAdes. IDBA-trans also has rather small number of misassembled contigs (1015), but outputs a very frag-

Table 4. Benchmarking of **BinPacker**, **Bridger**, **IDBA**-tran, RNA-**Bloom**, **rnaSPAdes**, **SOAP**denovo-Trans, **SPAdes**, Trans-**ABySS** and **Trinity** on real Human dataset. The annotated transcriptome of *H. sapiens* GRCh38.82 consists of 57820 genes and 196520 isoforms. All contigs shorter than 200 bp were filtered out prior to the analysis. The best values for each metric are highlighted with bold.

	BinPacker	Bridger	IDBA	Bloom	rnaSPAdes	SOAP	SPAdes	ABySS	Trinity
Transcripts	144598	191459	173330	239912	167710	140769	223917	190798	234074
Misassemblies	9898	7487	1015	1643	2111	**450**	3190	916	5183
Duplication ratio	2.03	1.61	1.02	2.75	1.36	1.12	**1.01**	1.25	2.00
Database coverage, %	17.2	16.6	19.6	**24.8**	21.3	18.5	18.4	22.5	24.2
50%-assembled genes	10763	10534	11712	12779	**13377**	12154	12395	12621	12902
95%-assembled genes	4457	4226	1334	6121	**7094**	5051	4427	4844	5398
50%-assembled isoforms	15133	14032	16260	**22547**	18619	15302	15533	19817	21876
95%-assembled isoforms	5080	4680	1338	7976	**8026**	5259	4455	5046	6753

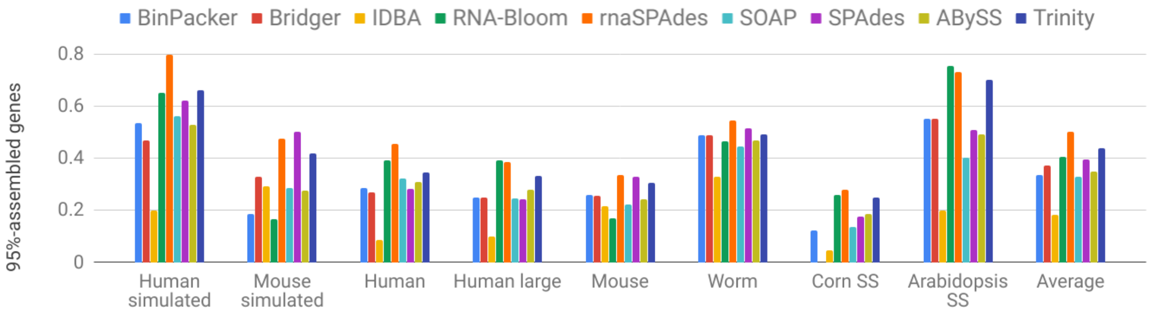

Figure 2. The fraction of 95%-assembled genes presented as bar plots for all generated assemblies. The values are computed relative to the number of genes reported by kallisto [25] that have per-base coverage depth > 5. The last columns show average values over all datasets. Note, that Bridger failed to assemble Corn dataset.

mented assembly with the lowest number of 95%-assembled genes/isoforms.

Figure 2 demonstrates fraction of 95%-assembled genes in all generated assemblies and mean values for each assembler across all datasets. RnaSPAdes, Trinity and RNA-Bloom show stable performance across different datasets and have the highest fraction of 95%-assembled genes on average (0.5, 0.438 and 0.406 respectively). While genomic SPAdes also has high value on average (0.397), it is mostly achieved by decent performance of simulated data. Figure 2 shows that the fraction of 95%-assembled genes for simulated datasets is typically higher comparing to the respective values for real data, most likely due to the absence of sequencing artifacts. Additionally, *de novo* assemblies of complex organisms, such as *H. sapiens* and *M. musculus*, tend to have lower fractions of assembled genes in comparison to *C. elegans* and *A. thaliana*. For example, Human large dataset has smaller values than the ones for Worm assemblies, although the later one have almost 3x lower coverage.

Table 5. Running time and peak RAM usage for **BinPacker**, **Bridger**, **IDBA**-tran, RNA-**Bloom**, **rnaSPAdes**, **SOAP**denovo-Trans, **SPAdes**, Trans-**ABySS** and **Trinity** on Human large and Arabidopsis datasets (125 and 118 million read-pairs respectively). All assemblers we launched in 16 threads on a server with 128 GB of RAM and 56 Intel Xeon 2.0 GHz cores. BinPacker, which has no options for setting the number of threads, was launched with default parameters. The best values for time and RAM are highlighted with bold.

	Human large		Arabidopsis	
Assembler	Time	RAM	Time	RAM
BinPacker	46 h 59 m	91 GB	88 h 25 m	131 GB
Bridger	65 h 54 m	88 GB	49 h 58 m	126 GB
IDBA	9 h 35 m	35 GB	26 h 24 m	42 GB
Bloom	37 h 52 m	38 GB	34 h 42 m	40 GB
rnaSPAdes	5 h 4 m	32 GB	7 h 24 m	40 GB
SOAP	1 h 21 m	28 GB	1 h 58 m	20 GB
SPAdes	11 h 39 m	39 GB	14 h 58 m	52 GB
ABySS	6 h 49 m	25 GB	8 h 9 m	35 GB
Trinity	18 h 8 m	50 GB	8 h 30 m	123 GB

Computational performance

To compare selected assemblers in terms of computational performance, we measured their running time and RAM consumption on two largest datasets using system utilities rather than using log files. As Table 5 indicates, SOAPdenovo-Trans is at least 3 times faster than any other assembler and have one of the lowest memory requirements (less than 30 GB for both datasets). Trans-ABySS and rnaSPAdes have comparable performance, with rnaSPAdes being slightly faster and more greedy regarding RAM consumption. Other assemblers typically have longer running time (at least 2 times more than rnaSPAdes in most cases) and higher memory requirements. Among all tools, BinPacker, Bridger and Trinity have the the highest peak RAM, e.g. more than 100 GB of Arabidopsis dataset.

Discussion

Quality reports provided in this manuscript (Tables 3 and 4) and Supplementary material (Tables S1-S8, Figures S1-S5) contain large variety of metrics that reflect completely different assembly properties, importance of which may vary depending on the further analysis and the entire pipeline being used. We believe that one of the key features of the *de novo* transcriptome assembler is the ability to correctly capture the entire transcript into a single contig (e.g. reflected by the number of 95%-assembled genes/isoforms, contig recall). On the other hand, such metrics as gene database coverage, number of covered reference proteins and nucleotide recall do not reflect this significant property, since they account *all* contigs mapped to a specific gene or protein and do not include assembly contiguity. For example, high database coverage or nucleotide recall can be achieved by a very fragmented assembly (or even raw

reads), which, indeed, does not suit well for further reference-free analysis.

Below we attempt to summarize these results and highlight strong and weak points of different assemblers.

Comparison between SPAdes and rnaSPAdes

In comparison to the original version of SPAdes, rnaSPAdes dominates by the majority of metrics. More precisely, it has significantly better assembly completeness metrics: 26% higher average fraction of 95%-assembled genes, 18% larger database coverage, 30% higher contig recall reported by REF-EVAL and 18% more detected BUSCOs. It also shows 18% higher contig precision on average, better reference coverage metrics reported by Transrate (50% / 95%-covered reference proteins, reference coverage) and typically fewer misassemblies (except for Corn SS and Human large datasets). Due to aggressive overlap removal stage, SPAdes always has smaller mean duplication ratio (2% vs 32% for rnaSPAdes), fewer duplicated BUSCOs (1% vs 16% on average), percentage of uncovered bases (2% vs 19%) and higher nucleotide precision (0.66 vs 0.56).

Simulated Mouse dataset is the only one where original SPAdes generates more assembled genes and isoforms than rnaSPAdes. Detailed investigation showed that the key reasons are the low coverage of this data (11 million reads) and its artificial nature (rnaSPAdes assembles more genes on real Mouse data). By using small $k = 21$ during the first iteration SPAdes manages to assemble extremely low-covered genes, where overlaps between reads are short. Pitfalls of using small k-mer sizes in transcriptome assembly are discussed in the Methods section.

Finally, due to the exclusion of BayesHammer error correction module [26] and using only two k-mer sizes, rnaSPAdes pipeline appears to be about twice faster and consumes less RAM than usual SPAdes.

Assembly completeness

In comparison to other assemblers, rnaSPAdes shows the highest fraction of 95%-assembled genes and isoforms (0.5 and 0.32 respectively). Trinity (0.44 and 0.3) and RNA-Bloom (0.41 and 0.28) are ranked the second and the third according to these metrics (Supplementary Figure S3). These numbers correlate with the percentage of detected BUSCOs, for which rnaSPAdes also has the best average value across all datasets (74%), followed by Trinity (72%), Trans-ABySS (71%) and RNA-Bloom (68%).

The same assemblers typically form the top four according to various coverage metrics, such as database coverage provided by rnaQUAST, reference coverage, number of 50% / 95%-covered reference proteins and number of reference sequences with CBBR hits reported by Transrate (Supplementary Figures S3 and S5). For example, according to mean gene database coverage computed by rnaQUAST, Trinity has the highest value of 30.2%, with other assemblies having somewhat lower values: 29.6% for RNA-Bloom, 28.7% for rnaSPAdes and 24.2% for Trans-ABySS. Exactly the same ranking is defined by Transrate reference coverage: Trinity (27.8%), RNA-Bloom (26.9%), rnaSPAdes (24.4%) and Trans-ABySS (23.4%). Other assemblers typically show smaller values for completeness-related metrics, generating fragmented assemblies, like IDBA-tran, or having lower database coverage, e.g. BinPacker.

Nucleotide and contig recall metrics reported by Detonate generally support the conclusions stated above (Supplementary Figure S4). Thus, Trinity and rnaSPAdes have the best average nucleotide recall values (0.86 and 0.84 respectively). The maximal mean contig recall, however, is reported for RNA-Bloom (0.097), followed by Trinity (0.089), Trans-ABySS (0.087) and rnaSPAdes (0.079). To compute contig metrics Detonate keeps only the most reliable alignments with mapped fraction more than 99% (for both assembled and reference sequence). In contrast, rnaQUAST assigns contigs to known genes/isoforms and then counts ones that have at least X% covered by a single assembled contig. However, no cutoff is applied for mapped fraction of the assembled sequences in rnaQUAST. This difference between algorithms might explain the absence of perfect correlation between contig recall and number of 95%-assembled isoforms.

Assembly correctness

According to the number of misassembled contigs, the most accurate contigs are typically produced by SOAPdenovo-Trans, Trans-ABySS and IDBA-tran (see Supplementary figure S3d). Among these three, IDBA-tran, however, produces the most fragmented assemblies with the lowest average fraction of 95%-assembled genes equal to 0.18. Supplementary figure S3d also suggests that the highest numbers of misassemblies often belong to BinPacker, Bridger, RNA-Bloom and Trinity.

IDBA-tran, usual SPAdes and SOAPdenovo-Trans tend to provide assemblies with the smallest amount of duplicated sequences, which is confirmed by rnaQUAST duplication ratio (average values are 1.02, 1.02 and 1.07 respectively), percentage of duplicated BUSCOs (0.8%, 1% and 4.7%), fraction of uncovered bases reported by Transrate (0.018, 0.019 and 0.076) and Detonate's nucleotide precision (0.68, 0.66 and 0.66). Highest contig precision equal to 0.133, however, belongs to rnaSPAdes, followed by 0.129 for SOAPdenovo-Trans. The most duplicated assemblies according to these metrics are produced by RNA-Bloom, Trinity and BinPacker. In comparison to other assemblers, they have significantly higher mean duplication ratio (2.5, 1.77 and 1.71 respectively) and fraction of duplicated BUSCOs (40.6%, 31.4% and 29.7%), as well as lowest average nucleotide precision (0.37, 0.46 and 0.46). As to rnaSPAdes, according to duplication metrics and misassemblies, it neither fails, nor dominates, showing moderate average duplication ratio of 1.32 and fraction of duplicated BUSCOs equal 16.7%.

Indeed, beside completeness-related metrics, such as number of assembled genes and isoforms, metrics discussed above should be also considered during transcriptome quality evaluation, since erroneous and duplicated sequences may negatively affect further transcriptome analysis, such as gene annotation.

Read-based scores

According to the read-based scores reported by Transrate and Detonate RSEM EVAL which represent how well the assembly corresponds to the initial reads, rnaSPAdes also shows good results. Regarding the average Transrate contig score, conventional SPAdes has the highest average score equal to 0.31, followed by IDBA-trans and SOAPdenovo-Trans both having 0.17, and rnaSPAdes with 0.16. As to Detonate score, rnaSPAdes has the best average ($-3.45 \cdot 10^9$) with RNA-Bloom ($-3.46 \cdot 10^9$) and Trinity ($-3.84 \cdot 10^9$) being slightly behind. RNA-Bloom and Trinity, however, have the lowest Transrate average scores among all tools (0.026 and 0.084 respectively). Vice versa, SPAdes, IDBA and SOAPdenovo-Trans, which are the top three assemblers according to mean Transrate score, have the lowest three RSEM EVAL scores. Based on the complete quality reports presented in the Supplementary material, it appears that Transrate score mostly correlates with correctness-related metrics and is negatively affected by the presence of duplicated sequences, which explains highest average score for standard

SPAdes. In contrast, RSEM-EVAL score seems to correlate with assembly completeness metrics.

Conclusion

Although every transcriptome assembler presented in this study has its own benefits and drawbacks, the trade-off between assembly completeness and correctness can be significantly shifted by modifying the algorithms' parameters. For example, various thresholds for transcripts filtration in rnaSPAdes (Table S14 in the Supplementary material) result in assemblies with different properties. Also, varying *k*-mer size or incorporating iterative de Bruijn graph construction in rnaSPAdes may significantly affect the assembly quality (Tables S9-S11 in the Supplementary material). Thus, the parameters of the assembly algorithms can be varied in order to achieve the desired completeness or correctness characteristics and make the method to dominate by a certain group of metrics.

While the developed algorithm, rnaSPAdes, typically shows stable results across analyzed RNA-Seq datasets and often allows to capture more genes and isoforms than any other tool, there is no clear winner according to all metrics. Thus, the selection of the assembler may be varied depending on the goals of the particular research project and the sample preparation protocols being used, as well as secondary parameters, such as usability and computational performance. Even with the aid of specially developed tools, such as Transrate, DETONATE, BUSCO and rnaQUAST, the choice of a suitable assembly tool remains a non-trivial problem and may require additional benchmarks in each particular case.

Potential implications

Although the developed approach was initially designed for RNA-Seq data obtained from a single organism, it can be potentially applied for metatranscriptome assembly of samples collected from bacterial communities. Indeed, metatranscriptome assembly does not require reconstructing complex alternatively spliced isoforms, but implies other computational challenges, such as repetitive patterns in different genes (including homologous genes from various strains) and extreme differences in mRNA quantities [27, 28], which are caused by both — varying expression levels and abundances of different species. Improving the assembly algorithms, as well as designing an appropriate pipeline for quality evaluation of metatranscriptomic assemblies, are the possible further implications of this work.

Recently emerged long read protocols for mRNA sequencing allow to capture full-length transcripts without the assembly [29]. However, high error rate of Oxford Nanopore and PacBio sequencers prevents using output reads directly as complete transcripts. Typically, mapping to the reference genome, additional error-correction by short accurate Illumina reads or consensus construction is performed to obtain and further analyze high-quality sequences [30, 31, 32, 33, 34]. Combining rnaSPAdes with previously developed hybridSPAdes approach for joint assembly of short and long reads [35] may result into a viable alternative to the existing methods for processing long error-prone RNA reads.

In addition, benchmarking reports presented in this work can be used by the researchers for selecting the appropriate assembly method that meets their specific criteria and for better understanding of transcriptome assembly quality evaluation, such as, for example, correlation of different metrics.

Methods

Most of the modern *de novo* genome assembly algorithms for short reads rely on the concept of the de Bruijn graph [36]. While the initial study proposed to look for an Eulerian path traversing the de Bruijn graph in order to reconstruct genomic sequences, it appeared to be rather impractical due to the presence of complex genomic repeats and sequencing artifacts, such as errors and coverage gaps. Instead, genome assemblers implement various heuristic approaches, most of which are based on coverage depth, graph topology and the fact that the genome corresponds to one or more long paths traversing through the graph [37, 14]. Indeed, the later observation is not correct for the case of transcriptome assembly, in which RNA sequences correspond to numerous shorter path in the graph. Thus, to enable high-quality assemblies from RNA-Seq data the majority of procedures in the SPAdes pipeline have to undergo major alterations.

SPAdes genome assembler consists of the following major steps: (i) construction of the condensed de Bruijn graph, (ii) graph simplification, which implies removing chimeric and erroneous edges, (iii) mapping read pairs to the assembly graph, and (iv) repeat resolution and scaffolding using aligned paired reads with exSPAnder algorithm [38, 39]. While graph construction and mapping paired reads do not depend on the dataset type and requires no change for RNA-Seq data, graph simplification and repeat resolution procedures strongly rely on the properties of genomic sequences and thus require significant modifications and novel functionality for *de novo* transcriptome assembly. Below we describe the key changes introduced in rnaSPAdes.

Simplification of the de Bruijn graph in rnaSPAdes

During the graph simplification stage erroneous edges are removed from the de Bruijn graph based on various criteria in order to obtain clean graph containing only correct sequences (further referred to as an *assembly graph*). In the SPAdes pipeline the simplification process includes multiple various procedures that can be classified into three types: (i) trimming *tips* (dead-end or dead-start edges), (ii) collapsing *bulges* (alternative paths) and (iii) removing *erroneous connections* (chimeric and other false edges). In this section we present alterations introduced in rnaSPAdes simplification pipeline. We also provide comparison between initial and improved simplification procedures on several RNA-Seq datasets in the Supplementary material (Table S10).

Trimming tips

In the de Bruijn graph constructed from DNA reads the major fraction of tips (edges starting or ending at a vertex without other adjacent edges) typically correspond to sequencing errors and thus have to be removed. Since only a few tips are correct and either represent chromosome ends or formed by coverage gaps, the existing genome assemblers implement rather aggressive tip clipping procedures [37, 13] assuming that coverage gaps appear rather rarely. However, in the de Bruijn graph built from RNA-Seq data a significant amount of tips correspond to transcripts' ends and thus have to be preserved. In order to keep correct tips and obtain full-length transcripts, rnaSPAdes uses lower coverage and length thresholds for tip trimming procedure than SPAdes (see details below).

In some cases, tips originate from sequencing errors in multiple reads from highly-expressed isoforms and thus may have coverage above the threshold. While genome assemblers may also exploit relative coverage cutoff to remove such tips, in transcriptome assembly this approach may result in trimming

correct tips corresponding to the ends of low-expressed isoforms. However, erroneous tips typically have a small difference from the correct sequence without errors (e.g. 1-2 mismatches). To address this issue, we align tips to the alternative (correct) edges of the graph (Fig. 3a) and trim them if the identity exceeds a certain threshold (similar procedure is implemented in truSPAdes, which was designed for True Synthetic Long Reads assembly [40]). In case when two tips correspond to the starts/ends of an alternatively spliced isoforms, it is highly unlikely for them to have similar nucleotide sequences (Fig. 3b). Such tips are preserved during graph simplification procedure thus allowing to restore isoforms that differ only by starting or terminating exons.

Another specifics of RNA-seq datasets is the large number of low-complexity regions that originate from poly-A tails resulting from polyadenylation at the ends of mRNAs. In order to avoid chimeric connections and non-informative sequences, we also remove low-complexity edges from the de Bruijn graph (see exact criterion below).

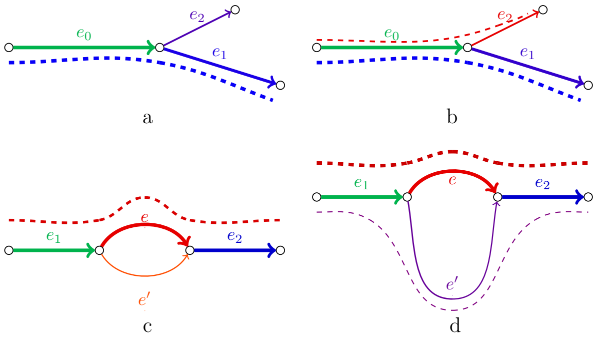

Figure 3. Examples of tips and bulges in the condensed de Bruijn graph. Edges with similar colors have similar sequences; line width represents the coverage depth. (a) Correct transcript (blue dashed line) traverses through edges e_0 and e_1. Edge e_2 is originated from the reads with the same sequencing error and thus has coverage depth high enough not to be trimmed. However, since the sequence of edge e_2 is very similar to the sequence of the alternative edge e_1 (detected by alignment), e_2 is eventually removed as erroneous. (b) In this case both paths (e_0, e_1) and (e_0, e_2) correspond to correct isoforms (blue and red dashed lines). Since the sequences of e_1 and e_2 are likely to be different, none of the correct tips is removed. (c) Correct sequence (red dashed line) traverses through edges e_1, e and e_2. Edge e' is originated from reads containing sequencing errors, and thus has sequence similar to e, but significantly lower coverage. (d) Both paths (e_1, e, e_2) and (e_1, e', e_2) correspond to different isoforms of the same gene (red and purple dashed lines); edges e and e' typically have different length, coverage depth and sequence.

Below we summarize all conditions used in tip clipping procedure, parameters for which were optimized based on our analysis of various RNA-seq datasets. We define l_T as the length of the tip that is being analyzed and c_T as its mean k-mer coverage, and c_A as the k-mer coverage of the alternative edge (which is presumably correct) A tip is removed if any of the following conditions is true:

- $l < 2 \cdot k$ and $c_T \leq 1$ (short tips with very low coverage);
- $l < 4 \cdot k$, $c_T < c_A/2$ and the Hamming distance between the tip and the alternative edge does not exceed 3 (the tip containing sequencing errors);
- the tip contains more than 80% of A/T nucleotides (low complexity tip).

Collapsing bulges

A simple bulge (two edges sharing starting and terminal vertices) in the de Burijn graph may correspond to one of the following events: (i) a sequencing error, (ii) a heterozygous mutation or another allele difference, or (iii) an alternative splicing event (typical for transcriptomic data). The first two cases are characterized by the bulge edges having similar lengths and sequences. However, edges formed by sequencing errors are typically short and have significantly different coverage depth, since it is unlikely for the same error to occur numerous times at the same position (Fig. 3c). Vice versa, in the case of allele difference bulge edges usually have similar coverage. Thus, genome assembly algorithms for bulge removal typically rely on the coverage depth [37, 13]. Since the most typical difference between two alternatively spliced isoforms of the same gene is the inclusion/exclusion of a an exon (usually short), edges of the bulge originated from these isoforms have different lengths (Fig. 3d). At the same time, since the expression levels may vary for such isoforms, the coverage depth may significantly differ. To avoid missing alternatively spliced isoforms in the assembly, rnaSPAdes does not use any coverage threshold for bulge removal and collapses only bulges consisting of edges with the similar lengths (less than 10% difference in length).

Removing chimeric connections

While undetected tips and bulges formed by sequencing errors result in mismatches and indels in the assembled contigs, chimeric reads (typically corresponding to a concatenation of sequences from distant regions of the original molecules) may trigger more serious errors, such as incorrect junctions in the resulting contigs (often referred to as misassemblies). In conventional genome assembly chimeric edges usually have low coverage and thus can be easily identified [37]. Single-cell datasets, however, feature multiple low-covered genomic regions and elevated number of chimeric reads, which result in numerous erroneous connections having higher coverage depth than correct genomic edges. Similarly, since true edges representing low-expressed isoforms in the transcriptome assembly also have relatively low coverage depth, cleaning the graph using coverage threshold will result in multiple missing transcripts in the assembly.

To detect chimeric connections in single-cell assemblies SPAdes implements various algorithms, which mostly rely on the assumption that each chromosome corresponds to a long contiguous path traversing through the de Bruijn graph [14]. Since this assumption does not hold for transcriptomes consisting of thousands isoforms, we had to disable most procedures for the chimeric edge detection in SPAdes and implement a new erroneous edge removal algorithm that addresses the specifics of chimeric reads in RNA-seq data sets.

Our analysis revealed that most of the chimeric connections in RNA-seq data can be divided into two groups: single-strand chimeric loops and double-strand hairpins. In the first case, a chimeric junctions connects the end of a transcript sequence with itself (Fig. 4a). The erroneous hairpin connects correct edge with its reverse-complement copy (Fig. 4b) and potentially may result in chimeric palindromic sequence in the assembly. To avoid misassemblies, rnaSPAdes detects chimeric loops and hairpins by analyzing the graph topology rather than nucleotide sequences or coverage.

While it remains unclear whether these chimeric reads are formed during transcription, RNA-seq sample preparation or sequencing, similar chimeric connections have been observed in the context of single-cell MDA. E.g., when a DNA fragment is amplified by MDA, the DNA polymerase moves along DNA molecule and copies it, but sometimes (as described in [15]), the polymerase may jump to a close position (usually on the opposite DNA strand) and proceed to copy from the new position.

Removing isolated edges

Another type of excessive edges that appear in the assembly graph are isolated edges, i.e. that have no adjacent edges. They

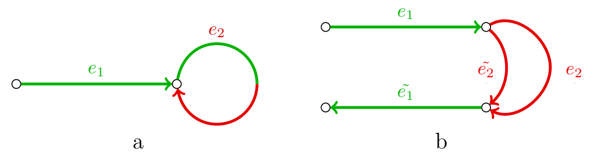

Figure 4. Examples of chimeric connections in the de Bruijn graph typical for transcriptome assembly. Red and green indicate erroneous and correct sequences respectively. (a) A chimeric loop (edge e_2) connecting end of the correct transcriptomic edge e_1 with itself. (b) An example of chimeric hairpin, where erroneous edge e_2 connects a correct edge e_1 with its reverse-complement copy $\tilde{e_1}$. Since e_2 connects a vertex and its reverse-complement, $\tilde{e_2}$ (the reverse-complement of e_2) also connects these two vertices.

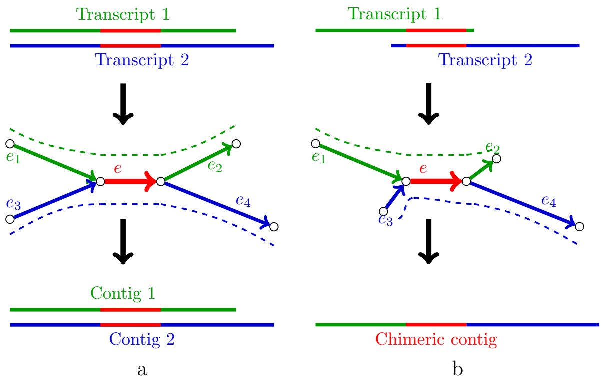

Figure 5. Examples of two transcripts having a common sequence (a) in the middle of the transcripts and (b) close to the start of one transcript and the end of another. While in the first case the isoforms can be resolved using read-pairs, the latter one may potentially result in a chimeric contig.

typically appear in the regions of extremely low-coverage (including DNA contamination), where overlaps between neighboring reads are smaller than k-mer size, or originate from reads containing zero correct k-mers due to multiple sequencing errors. The first type of isolated edges can possibly be connected with other edges by gap closing procedure (described below). The second type, on the other hand, may result in excessive erroneous sequences in the assembly or even create ambiguities during gap closing. Thus, during graph simplification we additionally remove isolated edges that have low coverage (< 2) and have length smaller or equal to read length.

Selecting optimal k-mer sizes

One of the key techniques that allows SPAdes to assemble contiguous genomic sequences from the data with non-uniform coverage depth is the iterative de Bruijn graph construction. During each next iteration, SPAdes builds the graph from the input reads and sequences obtained at the previous iteration, simplifies the graph and provides its edges as an input to the next iteration that uses larger k-mer size. Assembly graph obtained at the final iteration is used for repeat resolution and scaffolding procedures, which exploit read-pairs and long reads [38, 35]. In this approach small k-mer sizes help to assemble low-covered regions where reads have short overlaps, and large k values are useful for resolving repeats and therefore obtaining less tangled graph. Although this method seems to be useful for restoring low-expressed isoforms from RNA-Seq data, our analysis revealed that it appears to be the main reason of the high number of misassembled contigs in SPAdes assemblies. Below we describe how these false junctions are formed.

When two transcripts (possibly from different genes) have a common sequence in the middle, they form a typical repeat structure in the de Bruijn graph (Fig. 5a), which may further be resolved, e.g. using paired reads. However, if a common sequence appears close to the ends of the transcripts (Fig. 5b), edges e_2 and e_3 appear to be rather short and may be trimmed as tips (since coverage depth often drops near the transcripts ends), or may not be present at all. In this case, the remaining edges e_1, e and e_4 will be condensed into a single edge corresponding to chimeric sequence.

Indeed, since small k-mer size results in a higher chance of creating such kind of chimeric junction, we decided to modify the parameters for the iterative graph construction. In rnaSPAdes we decided to use only two k values: smaller one for restoring low-covered regions with insufficient overlaps between reads and larger one for obtaining less tangled graph.

To estimate the optimal k values, we ran rnaSPAdes on several RNA-Seq datasets with various read lengths sequenced from organisms with different gene complexity. Since it requires tremendous amount of time to try all possible pairs of k-mer sizes on multiple datasets, we first estimated upper k value used for the main iteration, and then selected lower k with the fixed upper one.

We assembled a number of datasets using only a single k-mer size and selected the best assemblies according to number of assembled genes, database coverage and number of misassemblies. Although it may be not possible to choose a single best k value simultaneously for multiple datasets, nearly optimal k-mer size was estimated as half of the read length (more precisely, the largest odd number that does not exceed $read\_length/2 - 1$). The smaller k value was estimated in a similar manner with the fixed upper k-mer size. Optimal lower k was considered based on number of additional assembled genes and misassemblies. Experiments showed that small k values (e.g. below 29) tend to dramatically increase the number of erroneous contigs due to the higher probability of two transcripts sharing the same k-mer. Thus, the lower k-mer size was estimated approximately as $read\_length/3$ with the minimal possible value set to 29. Although estimated k values may not provide the best assembly for every dataset, they typically appear to be a good trade-off between the number of recovered genes and generated errors (see Supplementary Tables S7-S9).

In this work rnaSPAdes was launched with the default k values. Indeed, rnaSPAdes keeps the possibility to set the k-mer sizes manually. In addition, we introduced the `--fast` option, which forces the assembler to use only a single k value (the optimal upper k). Typically, assemblies obtained with a single k capture fewer genes and isoforms (especially low-covered), but also have smaller number of misassembled contigs (see Supplementary material for comparison).

In order to preserve correct connections that could be restored using only small k-mer sizes, we carefully examined low-expressed transcripts that were not completely assembled using default k-mer sizes. The analysis revealed that the majority of such fragments can be joined by the small overlap, which is often confirmed by the read-pairs. To perform the gap closing procedure rnaSPAdes glues two tips if one of the following conditions is true:

- tips have an exact overlap of length at least L_{ov} and are connected by at least N_{ov} read pairs;
- tips are connected by at least N_{min} read pairs.

where the default parameters are $L_{ov} = 8$ bp, $N_{ov} = 1$ and $N_{min} = 5$. Although these parameters seem to be slightly ad-hoc, such gap closing procedure appears to be a viable alternative to using small k values and allows to restore more low-expressed transcripts without increasing the number of misassemblies. Using smaller thresholds for gap closing often create false connections and increase the amount of erroneous tran-

scripts, while larger values for these parameters result in a smaller increase of reconstructed sequences.

Isoform reconstruction

Adapting repeat resolution algorithms

Genomic repeats present one of the key challenges in the *de novo* genome assembly problem. Although mRNA sequences typically do not contain complex repeats, transcriptome assembly has a somewhat similar problem of resolving alternatively spliced isoforms and transcripts from paralogous genes. Repeat resolution and scaffolding steps in SPAdes genome assembler are implemented in the exSPAnder module [38], which is based on simple path-extension framework. Similar to other modules of SPAdes, exSPAnder was designed to deal with highly uneven coverage and thus can be adapted for isoform detection procedure when assembling RNA-Seq data.

The key idea of the path-extension framework is to iteratively construct paths in the assembly graph by selecting the best-supported extension edge at every step until no extension can be chosen. The extension is selected based on the scoring function that may exploit various kinds of linkage information between edges of the assembly graph (different scoring functions are implemented for different types of sequencing data). A situation when a path cannot be extended further is usually caused by the presence of long genomic repeat or a large coverage gap. The extension procedure starts from the longest edge that is not yet included in any path and is repeated until all edges are covered.

More formally, a path extension step can be defined as follows. For a path P and its extension edges $e_1, \ldots, e_n$ (typically, edges that start at the terminal vertex of P) the procedure selects e_i as a best-supported extension if

i. $Score_P(e_i) > C \cdot Score_P(e_j)$ for all $j \neq i$
ii. $Score_P(e_i) > \Theta$

where C and Θ are the algorithm parameters, and $Score_P(e_i)$ is a score of edge e_i relative to path P (described in [38]).

In contrast to genome assembly, in which there is usually only one true extension edge, in transcriptome assembly multiple correct extensions are possible due to the presence of alternatively spliced isoforms. Thus, the modified procedure is capable of selecting several edges $e_{k_1}, \ldots e_{k_m}$ among all possible extensions $e_1, \ldots, e_n$, which satisfy the following conditions:

i. $Score_P(e_{k_i}) > Score_P(e_M)/C$ for all $i = 1 \ldots m$,
$M = argmax_{j=1..n} Score_P(e_j)$
ii. $Score_P(e_{k_i}) > \Theta$ for all $i = 1 \ldots m$

Namely, all correct extension edges must have a score close to the maximal one ($C = 1.5$ by default), and the second condition remains the same. Afterwards, the algorithm extends path P by creating new paths $(P, e_{k_1}), \ldots, (P, e_{k_m})$, which are then extended independently. Since the scoring function implemented in exSPAnder does not strongly depend on the coverage depth, there is no danger that highly-expressed isoforms will be preferred over the low-expressed ones.

Finally, to avoid duplicating sequences in the genome assemblies, exSPAnder performs rather aggressive overlap removal procedure. However, since alternatively spliced isoforms may differ only by a short exon, in order to avoid missing similar transcripts the modified overlap detection procedure removes only exact duplicates and sub-paths.

Exploiting coverage depth

Varying coverage depth may seem to be an additional challenge for *de novo* sequence assembly, but can be also used as an advantage in some cases. For instance, if two alternatively spliced isoforms of the same gene have different expression levels, they can be resolved using coverage depth even when the read-pairs do not help (e.g. shared exon is longer than the insert size). Although using coverage values becomes more complicated when a gene has multiple different expressing isoforms, our analysis of several RNA-Seq datasets revealed that such cases are rather rare and most of the genes have one or two expressing isoforms within a single sample.

To exploit the coverage depth we decided to add a simple, but reliable path-extension rule. Let the path $P = (e_1, e_2, e_3)$ have extension edges e and e' (Fig. 6a), such that $cov(e) > cov(e')$ and $cov(e_2) > cov(e'_2)$, where $cov(e)$ denotes the k-mer coverage of edge e. To select a correct extension the algorithm detects a vertex closest to the end of path P that has two incoming alternative edges, one of which is included in P and another is not (e_2 and e'_2 in this example). Since edge $e_2 \in P$ has higher coverage than the alternative edge $e'_2 \notin P$, we select extension edge e as the one with the higher coverage. However, if both isoforms have similar coverage, this simple approach may chose a false extension (since the coverage depth is rarely perfectly uniform even along a small region). Thus the difference in coverage should be significant enough to distinguish between the isoforms. More formally, the following conditions should be satisfied:

i. $cov(e) > \Delta \cdot cov(e')$
ii. $cov(e_2) > \Delta \cdot cov(e'_2)$
iii. $\Omega > cov(e_2)/cov(e) > 1/\Omega$
iv. $cov(e) > C_{min}$

where the default values of the algorithm parameters are $\Delta = 2$, $\Omega = 10$ and $C_{min} = 2$. The first two conditions ensure that the extension edges (e and e') and alternative edges (e_2 and e'_2) have significant coverage difference, the third one requires the coverage depth to remain relatively persistent along the path and the latter one prevents the algorithm from resolving low-covered isoforms (which may result in a misassembly). In general case, this procedure also utilizes only the last pair of alternative edges, and is applied only in case when the path has two possible extension edges and conventional read-pair extender fails to extend the path.

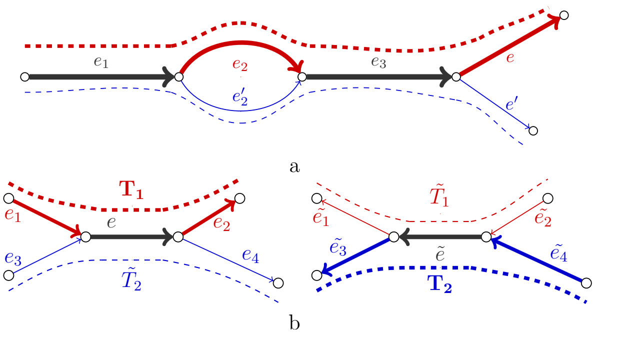

Figure 6. Using coverage depth for isoform reconstruction. Line width represents conventional and strand-specific coverage depths in figures (a) and (b) respectively. (a) Two isoforms of the same gene (red and blue dashed lines) have different expression levels and thus can be resolved using coverage depth. (b) Two transcripts T_1 and T_2 (red and blue bold dashed lines respectively) share a reverse-complement sequence and thus can be resolved using strand-specific reads.

Assembling strand-specific data

Another possible way to improve a transcriptome assembly is to take the benefit of strand-specific data when provided. To utilize stranded RNA-Seq we introduce *strand-specific coverage depths* $cov^+(e)$ and $cov^-(e)$, which denote k-mer coverage of edge e by forward and reverse reads respectively. As opposed to the conventional coverage $cov(e)$, which is calculated by aligning all reads and their reverse-complement copies to the edges of the assembly graph (thus making $cov(e) = cov(\tilde{e})$), strand-specific coverage is obtained by mapping reads according to their origin strand. For instance, if an RNA-Seq library is constructed in such way that reads have the same strand as the transcript which they were sequenced from (sense/forward reads), we expect $cov^+(e)$ to be much higher than $cov^-(e)$ if the sequence of e corresponds to the transcript, and vice versa if e is the reverse-complement of the original transcript. Indeed, the situation becomes opposite when reads are sequenced from cDNAs that are reverse-complement to the original transcripts (anti-sense/reverse reads). When working with paired-end libraries, we assume that the type of the library is defined by the first read's strand (i.e. forward-reverse or reverse-forward). Thus, the second read in pair is reverse-complemented before mapping in order to match the strand of the first read.

To extend the paths we apply the same path-extension procedure described above for conventional coverage, but use strand-specific coverage values instead. Fig. 6b demonstrates a situation, when two transcript correspond to paths $T_1 = (e_1, e, e_2)$ and $T_2 = (\tilde{e_4}, \tilde{e}, \tilde{e_3})$. If the repetitive edge e is longer than the insert size and the conventional coverage depth of these two transcripts is similar, the situation cannot be resolve neither by paired reads, nor by coverage. However, in case of stranded data, strand-specific coverage for actual transcripts' paths will be much higher than for their reverse-complement copies, i.e. $cov^+(T_1) >> cov^+(\tilde{T_1})$ and $cov^+(T_2) >> cov^+(\tilde{T_2})$ (in this example we assume that reads have the same stand as the transcripts they come from). Moreover, edges corresponding to the reverse-complement sequences only ($\tilde{e_1}$ and $\tilde{e_2}$ for $\tilde{T_1}$, e_3 and e_4 for $\tilde{T_2}$) will have $cov^+(e)$ values close to zero. Therefore, the conditions given for coverage-based path extender (see previous subsection) will be satisfied for strand-specific coverage values, the repetitive edge e will be resolved and both transcripts will be reconstructed.

To avoid collapsing transcripts from the opposite strands that share common sequences at their ends, we also split edges that have significantly different strand-specific coverage values at their ends. More formally, edge e is splitted at position p if $cov^+(e[0, p]) >> cov^-(e[0, p])$ and $cov^-(e[p + 1, length(e)]) >> cov^+(e[p + 1, length(e)])$ (or vise versa), where $e[i, j]$ is defined as a region of edge e starting from i and ending at j.

In addition, for stranded RNA-Seq data we output the paths constructed by the exSPAnder algorithm according to the original transcript's strand. E.g. in the example given in Figure 6b rnaSPAdes will output paths T_1 and T_2, since they have higher strand-specific coverage than their reverse complement copies ($\tilde{T_1}$ and $\tilde{T_2}$ respectively).

Filtering assembled transcripts

Before outputting the paths constructed by the exSPAnder module as contigs, we additionally apply various filtering procedures in order to remove non-mRNA contigs, such as intergenic sequences, which often contaminate RNA-Seq datasets. Our analysis showed that the majority of such unwanted sequences have low coverage, relatively small length and often correspond to isolated edges in the assembly graph. However, applying filters based on these criteria may also remove correct low-expressed transcripts in some cases. Thus, we decided to implement three different presets of parameters for the filtration procedure (soft, normal and hard) and output three files with contigs (see exact parameters in the Supplementary Table S12). Depending on the project goal the researcher may choose more sensitive (soft filtration) or more specific results (hard filtration). Table S13 in the Supplementary material shows how the assembly quality depends on the filtration parameters. In other tables we use default transcripts with the normal level of filtering.

Software availability

Project name: rnaSPAdes
Project home page: cab.spbu.ru/software/rnaspades/, github.com/ablab/spades
Operating systems: Linux and MacOS
Programming language: C++, Python
Other requirements: no requirements for pre-compiled binaries; g++ 5.3.1+, cmake 2.8.12+, zlib and libbz2 are required for compiling from source code
License: GPLv2
RRID: SCR_016992

Availability of supporting data and materials

All real RNA-Seq datasets are available at short read archive (https://www.ncbi.nlm.nih.gov/sra) with the following accession numbers

- Human: SRR5133163
- Human large: SRR1957703, SRR1957706
- Mouse: SRX648736
- Worm: SRR1560107
- Corn: SRR1588569
- Arabidopsis: SRR5344669, SRR5344670

Simulated data is available on the server

- *H. sapiens*: http://spades.bioinf.spbau.ru/rnaspades/simulated_data/human/
- *M. musculus*: http://spades.bioinf.spbau.ru/rnaspades/simulated_data/mouse/

Declarations

Consent for publication

Not applicable.

Competing Interests

The authors declare that they have no competing interests.

Funding

The work was supported by Russian Foundation for Basic Research (grant number 19-04-01074) and St. Petersburg State University (grant number 15.61.951.2015).

Author's Contributions

Software design and implementation was performed by EB, DA and AP. EB was responsible for data curation, assemblers benchmarking and manuscript editing. AL supervised the project and performed funding acquisition. AP wrote the

manuscript and managed the project. All authors read and approved the final manuscript.

Acknowledgements

The authors would like to thank the staff of following organizations for uploading their data to public databases: Pfizer, Sun Yat-sen University, Shanghai University, Leipzig University, Northwestern University and Medical University Vienna.

References

1. Li B, Dewey CN. RSEM: accurate transcript quantification from RNA-Seq data with or without a reference genome. BMC bioinformatics 2011;12(1):323.
2. Trapnell C, et al. Differential gene and transcript expression analysis of RNA-Seq experiments with TopHat and Cufflinks. Nature protocols 2012;7(3):562.
3. Dobin A, et al. STAR: ultrafast universal RNA-Seq aligner. Bioinformatics 2013;29(1):15–21.
4. Kim D, et al. TopHat2: accurate alignment of transcriptomes in the presence of insertions, deletions and gene fusions. Genome Biol 2013;14(4):R36.
5. Love MI, Huber W, Anders S. Moderated estimation of fold change and dispersion for RNA-seq data with DESeq2. Genome biology 2014;15(12):550.
6. Pertea M, et al. StringTie enables improved reconstruction of a transcriptome from RNA-seq reads. Nature biotechnology 2015;33(3):290.
7. Robertson G, et al. De novo assembly and analysis of RNA-seq data. Nat Methods 2010;7(11):909–912.
8. Grabherr MG, et al. Full-length transcriptome assembly from RNA-Seq data without a reference genome. Nat Biotechnol 2011 Jul;29(7):644–652.
9. Schulz MH, et al. Oases: robust de novo RNA-seq assembly across the dynamic range of expression levels. Bioinformatics 2012;28(8):1086–1092.
10. Peng Y, et al. IDBA-tran: a more robust de novo de Bruijn graph assembler for transcriptomes with uneven expression levels. Bioinformatics 2013;29(13):i326–i334.
11. Xie Y, et al. SOAPdenovo-Trans: De novo transcriptome assembly with short RNA-Seq reads. Bioinformatics 2014;p. btu077.
12. Martin JA, Wang Z. Next-generation transcriptome assembly. Nat Rev Genet 2011 Oct;12(10):671–682.
13. Bankevich A, et al. SPAdes: A New Genome Assembly Algorithm and Its Applications to Single-Cell Sequencing. Journal of Computational Biology 2012;19:455–477. http://dx.doi.org/10.1089/cmb.2012.0021.
14. Nurk S, et al. Assembling Single-Cell Genomes and Mini-Metagenomes From Chimeric MDA Products. Journal of Computational Biology 2013;20:1–24.
15. Lasken RS. Single-cell genomic sequencing using Multiple Displacement Amplification. Current opinion in microbiology 2007 Oct;10:510–516. http://dx.doi.org/10.1016/j.mib.2007.08.005.
16. Bushmanova E, et al. rnaQUAST: a quality assessment tool for de novo transcriptome assemblies. Bioinformatics 2016;32(14):2210–2212.
17. Andrews S, et al., FastQC: a quality control tool for high throughput sequence data; 2010.
18. Bolger AM, Lohse M, Usadel B. Trimmomatic: a flexible trimmer for Illumina sequence data. Bioinformatics 2014;30(15):2114–2120.
19. Liu J, Li G, Chang Z, Yu T, Liu B, McMullen R, et al. BinPacker: packing-based de novo transcriptome assembly from RNA-seq data. PLoS computational biology 2016;12(2):e1004772.
20. Chang Z, Li G, Liu J, Zhang Y, Ashby C, Liu D, et al. Bridger: a new framework for de novo transcriptome assembly using RNA-seq data. Genome biology 2015;16(1):30.
21. Nip KM. RNA-Bloom: de novo RNA-seq assembly with Bloom filters. PhD thesis, University of British Columbia; 2017.
22. Smith-Unna R, et al. TransRate: reference-free quality assessment of de novo transcriptome assemblies. Genome research 2016;2(8):1134–1144.
23. Simão FA, et al. BUSCO: assessing genome assembly and annotation completeness with single-copy orthologs. Bioinformatics 2015;31(19):3210–3212.
24. Li B, et al. Evaluation of de novo transcriptome assemblies from RNA-Seq data. Genome Biol 2014;15(12):553.
25. Bray NL, Pimentel H, Melsted P, Pachter L. Near-optimal probabilistic RNA-seq quantification. Nature biotechnology 2016;34(5):525.
26. Nikolenko SI, Korobeynikov AI, Alekseyev MA. BayesHammer: Bayesian clustering for error correction in single-cell sequencing. BMC Genomics 2013;14.
27. Leung HC, Yiu SM, Parkinson J, Chin FY. IDBA-MT: de novo assembler for metatranscriptomic data generated from next-generation sequencing technology. Journal of Computational Biology 2013;20(7):540–550.
28. Leung HC, Yiu SM, Chin FY. IDBA-MTP: a hybrid Metatranscriptomic assembler based on protein information. Journal of Computational Biology 2015;22(5):367–376.
29. Garalde DR, Snell EA, Jachimowicz D, Sipos B, Lloyd JH, Bruce M, et al. Highly parallel direct RNA sequencing on an array of nanopores. Nature methods 2018;15(3):201.
30. Tilgner H, Grubert F, Sharon D, Snyder MP. Defining a personal, allele-specific, and single-molecule long-read transcriptome. Proceedings of the National Academy of Sciences 2014;p. 201400447.
31. Byrne A, Beaudin AE, Olsen HE, Jain M, Cole C, Palmer T, et al. Nanopore long-read RNAseq reveals widespread transcriptional variation among the surface receptors of individual B cells. Nature Communications 2017;8:16027.
32. Minoche AE, Dohm JC, Schneider J, Holtgräwe D, Viehöver P, Montfort M, et al. Exploiting single-molecule transcript sequencing for eukaryotic gene prediction. Genome biology 2015;16(1):184.
33. Abdel-Ghany SE, Hamilton M, Jacobi JL, Ngam P, Devitt N, Schilkey F, et al. A survey of the sorghum transcriptome using single-molecule long reads. Nature communications 2016;7:11706.
34. Gordon SP, Tseng E, Salamov A, Zhang J, Meng X, Zhao Z, et al. Widespread polycistronic transcripts in fungi revealed by single-molecule mRNA sequencing. PloS one 2015;10(7):e0132628.
35. Antipov D, et al. hybridSPAdes: an algorithm for hybrid assembly of short and long reads. Bioinformatics 2015;32(7):1009–1015.
36. Pevzner PA, et al. An Eulerian path approach to DNA fragment assembly. Proceedings of the National Academy of Sciences 2001;98:9748–9753. http://dx.doi.org/10.1073/pnas.171285098.
37. Zerbino DR, Birney E. Velvet: Algorithms for de novo short read assembly using de Bruijn graphs. Genome Research 2008;18:821–829. http://dx.doi.org/10.1101/gr.074492.107.
38. Prjibelski AD, et al. ExSPAnder: a universal repeat resolver for DNA fragment assembly. Bioinformatics 2014;30(12):i293–i301.
39. Vasilinetc I, et al. Assembling short reads from jumping libraries with large insert sizes. Bioinformatics

2015;31(20):3262–3268.
40. Bankevich A, Pevzner PA. TruSPAdes: barcode assembly of TruSeq synthetic long reads. Nature methods 2016;13(3):248.

Click here to access/download
Supplementary Material
Bushmanova.sup.pdf

Answers to the reviewers' comments

Click here to access/download
Supplementary Material
Answers_rnaSPAdes.pdf
