# Supplementary material for: rnaSPAdes: a de novo transcriptome assembler and its application to RNA-Seq data
Source: Gigascience. 2019 Sep 3;8(9):giz100. doi: 10.1093/gigascience/giz100 (PMC6736328; doi:10.1093/gigascience/giz100)
Supplement: giz100_Supplemental_File [file giz100_supplemental_file.pdf]

Supplementary Material: rnaSPAdes: a *de novo* transcriptome assembler and its application to RNA-Seq data

Elena Bushmanova [1], Dmitry Antipov [1], Alla Lapidus [1], and Andrey D. Prjibelski [1,*]

[1]Center for Algorithmic Biotechnology, Institute of Translational Biomedicine, St. Petersburg State University, St. Petersburg, Russia

S1 Transcriptome assembly quality evaluation

To assess quality of the assembled transcripts we used rnaQUAST (Bushmanova *et al.*, 2016), Transrate (Smith-Unna *et al.*, 2016) and DETONATE package (Li *et al.*, 2014), which were designed specifically for evaluating *de novo* transcriptome assemblies. While rnaQUAST focuses on the reference-based assessment and is useful for testing and benchmarking assemblies on organisms with known genomes sequences and gene database, the latter two tools are also capable of analyzing assemblies using only input reads. Since these tools produce reports containing a lot of various statistics, we selected only a few most significant metrics from each tool.

From rnaQUAST report we decided to select the following metrics:

- Transcripts — the total number of contigs generated by the assembler.
- Duplication ratio — the proportion of overlapping bases among transcripts assigned to the same isoform from the gene database. Since typically a significant fraction of isoforms may not express in the sequenced tissue, rnaQUAST counts only bases that are covered by at least one transcript, thus making an ideal value of the duplication ratio equal to 1. Higher values may reflect the presence of redundant transcripts reported by the assembler.
- Misassemblies — the number of transcripts with discordant alignments, e.g. partial alignments to different loci. A misassembly is reported only if it is confirmed by contig's alignments to the genome (with GMAP) and to the transcriptome (with BLASTN).
- Database coverage — the fraction of nucleotides from all database isoforms covered by all assembled transcripts.
- X% assembled genes/isoforms — the number of genes/isoforms that have at least X% captured by a *single* reported transcript. In case if several transcripts are assigned to a single gene/isoform, rnaQUAST selects the one with the best match.

We also decided to complement rnaQUAST report by the following reference-based Transrate metrics:

- P references with CRBB — the proportion of reference proteins having a CRB-BLAST hit to the assembled transcripts.
- Reference coverage — the proportion of reference protein bases covered by CRB-BLAST hits (correlates with rnaQUAST database coverage).

*To whom correspondence should be addressed. Andrey Prjibelski, e-mail: a.przhibelsky@spbu.ru

- 50% covered / 95% covered — the number of reference sequences with at least 50%/95% of their bases covered by all CRB-BLAST hits (correlates with rnaQUAST 50%-covered / 95%-covered isoforms).

We also added several metrics reported by REF-EVAL from DETONATE package (see Li *et al.* (2014) for details):

- precision, recall and $F1$-score at the contig/nucleotide level;
- k-mer recall and k-mer compression score (KC score).

Even though the benchmarks are performed on the organisms with high-quality reference genomes, we decided to provide several *de novo* metrics. Along with RSEM-EVAL score, we also include the following statistics obtained with Transrate:

- Contigs segmented — number of segmented sequences (i.e. potentially misassembled contigs);
- P bases uncovered — the proportion of transcript bases that are not covered by any reads;
- Contig score — a measure of how well the contigs are supported by read evidence;

In addition, we present the number of missing, fragmented and complete (both single-copy and duplicated) universal single-copy orthologs reported by BUSCO (Simão *et al.*, 2015) as bar plots (Figure S2).

S2 Tools versions used in this work

- BinPacker 1.0 (Liu *et al.*, 2016)
- Bridger 2014-12-01 (Chang *et al.*, 2015)
- IDBA-tran 1.1.3 (Peng *et al.*, 2013)
- RNA-Bloom 0.9.8 (Nip, 2017)
- SOAPdenovo-Trans 1.04 (Xie *et al.*, 2014)
- Trans-ABySS 2.0.1 (Robertson *et al.*, 2010)
  BLAT 36 (Kent, 2002)
- Trinity 2.6.6 (Grabherr *et al.*, 2011)
  Jellyfish 2.2.8 (Marçais and Kingsford, 2011)
  Salmon 0.9.1 (Patro *et al.*, 2017)
  Bowtie 2.3.4.1 (Langmead and Salzberg, 2012)
- rnaSPAdes and SPAdes from SPAdes 3.13.1 package
- rnaQUAST 1.5.2 (Bushmanova *et al.*, 2016)
  GMAP 2018-03-25 (Wu and Watanabe, 2005)
  BLAST package 2.6.0 (Camacho *et al.*, 2009)
- Transrate 1.0.3 (Smith-Unna *et al.*, 2016)
  Salmon 0.9.1 (Patro *et al.*, 2017)
  BLAST package 2.6.0 (Camacho *et al.*, 2009)
  SNAP aligner 1.0dev.96 (Zaharia *et al.*, 2011)

- DETONATE 1.10 (Li *et al.*, 2014)

    Bowtie 2.3.4.1 (Langmead and Salzberg, 2012)

    BLAT 36 (Kent, 2002)

- BUSCO 3.0.1 (Simão *et al.*, 2015)
- FastQC 0.11.7 (Andrews *et al.*, 2010)
- Trimmomatic 0.35 (Bolger *et al.*, 2014)
- kallisto 0.44.0 (Bray *et al.*, 2016)
- GeneMarkS-T 5.1 (Tang *et al.*, 2015)

S3 Command lines for running the tools

Quality control and preprocessing

- FastQC
  ```
  fastqc left.fastq right.fastq
  ```
- Trimmomatic
  ```
  TrimmomaticPE left.fastq right.fastq -baseout trimmed.fastq LEADING:3 TRAILING:3 SLIDINGWINDOW:4:15
  MINLEN:35
  ```

RSEM simulation based on real RNA-Seq

See https://github.com/deweylab/RSEM#simulation for recommendations.

```
rsem-prepare-reference --gtf gene_database.gtf --bowtie2 reference_genome.fa reference_label
rsem-calculate-expression --bowtie2 --paired-end left.fastq right.fastq reference_label sample_name
rsem-simulate-reads reference_label sample_name.stat/sample_name.model sample_name.isoforms.results
sample_name.stat/sample_name.theta NR simulated_reads
```

Assemblers

- Trans-ABySS

    For non-strand-specific data
  ```
  transabyss --pe left.fastq right.fastq --threads 16 --length 200 --outdir OUTPUT_DIR
  ```
    For strand-specific data we used --SS option.

- IDBA-tran
  ```
  fq2fa --merge --filter left.fastq right.fastq reads.fastq
  idba_tran -r reads.fastq -o OUTPUT_DIR --num_threads 16 --min_contig 200
  ```
- SOAPdenovo-Trans
  ```
  soap.config:
  max_rd_len=RL
  [LIB]
  avg_ins=INSERT_SIZE
  q1=left.fastq
  q2=right.fastq

  SOAPdenovo-Trans-31mer all -s soap.config -p 16 -L 200 -o OUTPUT_DIR/SOAP
  ```
  Note: scaffold sequences were used for the evaluation.

- Trinity

  For non-strand-specific data

  ```
  Trinity --seqType fq --max_memory 200G --left left.fastq --right right.fastq --CPU 16
  --min_contig_length 200 --output OUTPUT_DIR
  ```

  For strand-specific data we used `--SS_lib_type RF` option.

- rnaSPAdes

  For non-strand-specific data

  ```
  spades.py --rna -1 left.fastq -2 right.fastq -t 16 -o OUTPUT_DIR
  ```

  For strand-specific data we used `--ss-rf` option.

- SPAdes

  ```
  spades.py --sc -1 left.fastq -2 right.fastq -t 16 -o OUTPUT_DIR
  ```

  Note: scaffold sequences were used for the evaluation.

- BinPacker

  For non-strand-specific data

  ```
  BinPacker -d -q -s fq -p pair -l left.fastq -r right.fastq -o OUTPUT_DIR
  ```

  For strand-specific data we used `-m RF` option.

- Bridger

  For non-strand-specific data

  ```
  Bridger.pl --seqType fq --left left.fastq --right right.fastq --output OUTPUT_DIR --CPU
  16
  ```

  For strand-specific data we used `--SS_lib_type RF` option.

- RNA-bloom

  For non-strand-specific data

  ```
  java -jar RNA-Bloom.jar -left left.fastq -right right.fastq -revcomp-right -t 16 -length
  200 -outdir OUTPUT_DIR
  ```

  For strand-specific data we used `-stranded` option. For anti-sense data the resulting sequences were reverse-complemented since RNA-Bloom does not support strandness type.

Quality evaluation

- rnaQUAST

  ```
  rnaQUAST.py --transcripts transcripts_1.fa transripts_2.fa ...  --reference reference_genome.fa
  --gtf gene_database.gtf --output_dir OUTPUT_DIR --disable_infer_genes --disable_infer_transcripts
  --gene_mark
  ```

- DETONATE

  See http://deweylab.biostat.wisc.edu/detonate/vignette.html for recommendations.

  ```
  rsem-eval-estimate-transcript-length-distribution isoforms.fa species.txt

  rsem-eval-calculate-score --paired-end left.fastq right.fastq transcripts.fa rsem_eval_transcripts
  RL --transcript-length-parameters species.txt -p 16
  rsem-prepare-reference --bowtie isoforms.fa rsem_ref
  rsem-calculate-expression --paired-end left.fastq right.fastq -p 16 rsem_ref rsem_expr
  ref-eval-estimate-true-assembly --reference rsem_ref --expression rsem_expr --assembly
  ta --alignment-policy best
  ```

```
rsem-prepare-reference --bowtie ta_0.fa ta_0_ref
rsem-calculate-expression -p 16 --paired-end left.fastq right.fastq ta_0_ref ta_0_expr

ref-eval --scores kc --A-seqs transcripts.fa --B-seqs ta_0.fa --B-expr ta_0_expr.isoforms.results
--kmerlen RL --readlen RL --num-reads NR | tee kc_transcripts.txt

blat -minIdentity=80 ta_0.fa transcripts.fa transcripts_to_ta_0.psl
blat -minIdentity=80 transcripts.fa ta_0.fa ta_0_to_transcripts.psl

ref-eval --scores contig,nucl --weighted no --A-seqs transcripts.fa --B-seqs ta_0.fa --A-to-B
transcripts_to_ta_0.psl --B_to_A ta0_to_transcripts.psl --min-frac-identity 0/90 | tee
contig_nucl_transcripts.txt
```

Note: for Z.mays dataset we had to change detonate according to the authors suggestion given here: https://groups.google.com/forum/#!topic/detonate-users/IzfXjVttDPg

- Transrate

```
transrate --assembly transcripts_1.fa,transcripts_2.fa,...  --left left.fastq --right right.fastq
--reference peptides_database.fasta --threads 16 --output OUTPUT_DIR
```

- BUSCO

```
run_BUSCO.py -i transcripts.fa -o OUTPUT_DIR -l BUSCO_LINEAGE_DATA -m tran
```

- kallisto

For non-strand-specific data

```
kallisto index -i isoforms.idx isoforms.fa
kallisto quant -i isoforms.idx -o kallisto_quant -b 100 left.fastq right.fastq --threads
16
```

For strand-specific data we used `--rf-stranded` option.

S4 Quality reports

Table S1: Benchmarking of **BinPacker**, **Bridger**, **IDBA**-tran, RNA-**Bloom**, **rnaSPAdes**, **SOAP**denovo-Trans, **SPAdes**, Trans-**ABySS** and **Trinity** on Human simulated RNA-seq dataset. All contigs shorter than 200 bp were filtered out prior to the analysis. The annotated transcriptome of *H. sapiens* GRCh38.82 consists of 57820 genes and 196520 isoforms. The best values for each metric are highlighted with bold.

Assembler	BinPacker	Bridger	IDBA	Bloom	rnaSPAdes	SOAP	SPAdes	ABySS	Trinity
Options							`--sc`		
K	25	25	20-60		49, 73	23	21, 33, 55	32	25
**rnaQUAST metrics**									
Transcripts	76736	52151	58466	65968	37730	35096	42264	67511	62831
Misassemblies	7919	3512	174	358	309	198	443	**126**	1554
Duplication ratio	2.19	1.38	1.01	1.93	1.26	1.08	**1.00**	1.24	1.74
Database coverage, %	20.9	18.5	21.4	**24.6**	23.2	19.4	20.5	23.1	24.4
50%-assembled genes	11828	11476	13175	12869	**14075**	12610	13569	12740	13289
95%-assembled genes	7320	6417	2729	8910	**10934**	7685	8526	7225	9049
50%-assembled isoforms	17415	15423	18181	**21035**	19531	15638	16437	19250	20965
95%-assembled isoforms	9091	7298	2744	12108	**13387**	8151	8638	7662	12301
**Detonate scores**									
Nucleotide precision	0.39	0.68	**0.94**	0.47	0.74	0.87	0.93	0.73	0.52
Nucleotide recall	0.84	0.79	0.72	0.85	0.82	0.68	0.73	0.74	**0.87**
Nucleotide F1	0.54	0.73	0.81	0.61	0.78	0.76	**0.82**	0.73	0.65
Contig precision	0.09	0.128	0.085	0.137	**0.232**	0.214	0.177	0.117	0.128
Contig recall	0.057	0.054	0.04	**0.074**	0.071	0.061	0.061	0.064	0.066
Contig F1	0.07	0.08	0.055	0.096	**0.109**	0.095	0.091	0.083	0.087
k-mer recall	0.96	0.94	0.68	**0.99**	0.97	0.86	0.91	0.95	0.97
KC score	0.91	0.92	0.66	**0.95**	**0.95**	0.85	0.9	0.93	0.93
RSEM-EVAL score ($\times 10^9$)	-1.92	-2.08	-5.65	**-1.43**	-1.48	-2.98	-2.65	-2.02	-1.71
**Transrate comparative metrics**									
Contig score	0.00	0.09	0.16	0.03	0.15	0.14	**0.25**	0.10	0.05
P bases uncovered	0.78	0.43	**0.02**	0.66	0.37	0.15	**0.02**	0.33	0.64
Contigs segmented	5520	4860	3097	10521	6006	3513	**2356**	6513	6679
P references with CRBB	0.19	0.17	0.20	**0.21**	0.17	0.13	0.16	0.20	0.20
Reference coverage	0.25	0.23	0.18	**0.27**	0.23	0.17	0.18	0.23	**0.27**
50% covered	17862	15637	14625	**19149**	15819	11959	13133	16686	19005
95% covered	14492	12350	5338	**16007**	13057	7808	8747	11952	15696

Table S2: Benchmarking of **BinPacker**, **Bridger**, **IDBA**-tran, RNA-**Bloom**, **rnaSPAdes**, **SOAP**denovo-Trans, **SPAdes**, Trans-**ABySS** and **Trinity** on Mouse simulated RNA-seq dataset. All contigs shorter than 200 bp were filtered out prior to the analysis. The annotated transcriptome of *M. musculus* GRCm38.75 consists of 38924 genes and 94545 isoforms. The best values for each metric are highlighted with bold.

Assembler	BinPacker	Bridger	IDBA	Bloom	rnaSPAdes	SOAP	SPAdes	ABySS	Trinity
Options							`--sc`		
K	25	25	20-60	25	33, 49	23	21, 33, 55	32	25
**rnaQUAST metrics**									
Transcripts	24099	36945	34431	21908	35053	25535	38044	25761	43858
Misassemblies	3732	425	208	**5**	30	15	141	12	162
Duplication ratio	1.74	1.09	1.01	1.25	1.06	**1.00**	1.01	1.07	1.16
Database coverage, %	6.5	13.6	14.9	6.9	15.9	11.2	**17.4**	10.3	16.2
50%-assembled genes	3001	4888	5485	2981	6108	4696	**6514**	4186	5756
95%-assembled genes	1461	2567	2279	1302	3705	2226	**3916**	2151	3279
50%-assembled isoforms	3249	5135	5712	3299	6395	4811	**6712**	4570	6396
95%-assembled isoforms	1505	2640	2280	1386	3774	2229	**3921**	2190	3482
**Detonate scores**									
Nucleotide precision	0.51	0.85	0.92	0.78	0.89	**0.96**	0.9	0.89	0.77
Nucleotide recall	0.35	0.65	0.66	0.31	0.7	0.51	**0.76**	0.46	0.7
Nucleotide F1	0.41	0.73	0.77	0.44	0.78	0.67	**0.82**	0.61	0.73
Contig precision	0.074	0.12	0.135	0.081	**0.174**	0.141	0.155	0.118	0.123
Contig recall	0.016	0.041	0.043	0.016	**0.056**	0.033	0.054	0.028	0.049
Contig F1	0.027	0.061	0.065	0.027	**0.084**	0.053	0.08	0.045	0.07
k-mer recall	0.9	0.9	0.27	0.91	0.92	0.74	0.56	0.9	**0.95**
KC score	0.87	0.88	0.25	0.89	0.9	0.73	0.53	0.89	**0.92**
RSEM-EVAL score ($\times 10^9$)	-1.72	-1.68	-2.61	-1.7	**-1.62**	-1.93	-2.17	-1.72	-1.65
**Transrate comparative metrics**									
Contig score	0.01	0.12	0.06	0.03	0.11	0.13	**0.14**	0.12	0.03
P bases uncovered	0.07	0.03	**0.01**	0.04	**0.01**	**0.01**	**0.01**	0.02	0.06
Contigs segmented	566	894	780	1197	751	666	**321**	810	1860
P references with CRBB	0.12	0.19	0.19	0.13	0.19	0.16	**0.21**	0.16	**0.21**
Reference coverage	0.08	0.13	0.13	0.07	0.15	0.10	**0.16**	0.10	0.15
50% covered	5092	7757	7745	4378	8140	5969	**8692**	5914	8619
95% covered	3046	4593	4025	2474	5314	3200	**5551**	3518	5289

Table S3: Benchmarking of **BinPacker**, **Bridger**, **IDBA**-tran, RNA-**Bloom**, **rnaSPAdes**, **SOAP**denovo-Trans, **SPAdes**, Trans-**ABySS** and **Trinity** on Human RNA-seq dataset. All contigs shorter than 200 bp were filtered out prior to the analysis. The annotated transcriptome of *H. sapiens* GRCh38.82 consists of 57820 genes and 196520 isoforms. The best values for each metric are highlighted with bold.

Assembler	BinPacker	Bridger	IDBA	Bloom	rnaSPAdes	SOAP	SPAdes	ABySS	Trinity
Options							`--sc`		
K	25	25	20-60	25	49, 73	23	21, 33, 55	32	25
**rnaQUAST metrics**									
Transcripts	144598	191459	173330	239912	167710	140769	223917	190798	234074
Misassemblies	9898	7487	1015	1643	2111	**450**	3190	916	5183
Duplication ratio	2.03	1.61	1.02	2.75	1.36	1.12	**1.01**	1.25	2.00
Database coverage, %	17.2	16.6	19.6	**24.8**	21.3	18.5	18.4	22.5	24.2
50%-assembled genes	10763	10534	11712	12779	**13377**	12154	12395	12621	12902
95%-assembled genes	4457	4226	1334	6121	**7094**	5051	4427	4844	5398
50%-assembled isoforms	15133	14032	16260	**22547**	18619	15302	15533	19817	21876
95%-assembled isoforms	5080	4680	1338	7976	**8026**	5259	4455	5046	6753
**Detonate scores**									
Nucleotide precision	0.28	0.32	0.43	0.21	0.35	**0.43**	0.36	0.39	0.26
Nucleotide recall	0.84	0.83	0.73	0.88	0.84	0.72	0.76	0.78	**0.89**
Nucleotide F1	0.42	0.46	**0.55**	0.34	0.49	0.54	0.49	0.52	0.4
Contig precision	0.028	0.022	0.015	0.032	0.031	**0.036**	0.014	0.029	0.023
Contig recall	0.031	0.032	0.02	**0.059**	0.040	0.039	0.025	0.043	0.041
Contig F1	0.029	0.026	0.017	**0.042**	0.035	0.038	0.019	0.035	0.029
k-mer recall	0.86	0.86	0.63	**0.91**	0.89	0.8	0.78	0.87	0.88
KC score	0.81	0.82	0.6	0.84	**0.85**	0.78	0.74	0.83	0.82
RSEM-EVAL score ($\times 10^9$)	-3.23	-3.18	-6.76	-3.12	**-2.52**	-3.81	-4.69	-3.11	-2.99
**Transrate comparative metrics**									
Contig score	0.03	0.04	0.09	0.01	**0.11**	0.09	**0.11**	0.10	0.03
P bases uncovered	0.52	0.35	**0.05**	0.58	0.21	0.12	**0.05**	0.19	0.48
Contigs segmented	**14231**	22648	19908	28072	16299	20240	16450	22428	27960
P references with CRBB	0.19	0.18	0.21	0.23	0.18	0.14	0.16	**0.24**	0.23
Reference coverage	0.23	0.22	0.16	**0.27**	0.22	0.16	0.17	0.22	0.26
50% covered	16183	15231	13920	**19997**	15279	11826	13139	17036	19613
95% covered	11800	10971	4043	**14861**	11376	7221	6704	10707	13042

Table S4: Benchmarking of **BinPacker**, **Bridger**, **IDBA**-tran, RNA-**Bloom**, **rnaSPAdes**, **SOAP**denovo-Trans, **SPAdes**, Trans-**ABySS** and **Trinity** on Human large RNA-seq dataset. All contigs shorter than 200 bp were filtered out prior to the analysis. The annotated transcriptome of *H. sapiens* GRCh38.82 consists of 57820 genes and 196520 isoforms. The best values for each metric are highlighted with bold.

Assembler	BinPacker	Bridger	IDBA	Bloom	rnaSPAdes	SOAP	SPAdes	ABySS	Trinity
Options							`--sc`		
K	25	25	20-60	25	33, 49	23	21, 33, 55	32	25
**rnaQUAST metrics**									
Transcripts	157974	214502	206225	330948	225731	417161	226850	262474	280359
Misassemblies	8233	8048	825	9262	5023	**275**	3003	1138	4766
Duplication ratio	1.66	1.57	1.02	4.22	1.50	1.12	**1.01**	1.41	2.23
Database coverage, %	16.5	17.3	20.4	**26.5**	22.9	21.0	19.2	23.9	25.0
50%-assembled genes	11345	11619	12898	**14299**	14524	13028	13415	13866	14178
95%-assembled genes	4731	4769	1879	**7481**	7373	4690	4614	5347	6301
50%-assembled isoforms	15150	15534	17928	**27197**	21157	22187	17484	22549	24669
95%-assembled isoforms	5278	5288	1889	**10475**	8695	4925	4664	5560	7924
**Detonate scores**									
Nucleotide precision	0.34	0.33	**0.42**	0.14	0.31	0.37	0.39	0.35	0.24
Nucleotide recall	0.78	0.80	0.73	0.87	0.85	0.72	0.75	0.75	**0.86**
Nucleotide F1	0.48	0.46	**0.53**	0.24	0.45	0.49	0.51	0.48	0.37
Contig precision	**0.031**	0.025	0.021	0.027	0.029	0.023	0.020	0.030	0.023
Contig recall	0.025	0.028	0.022	0.045	0.034	**0.049**	0.023	0.040	0.033
Contig F1	0.028	0.026	0.022	**0.034**	0.031	0.031	0.021	**0.034**	0.027
k-mer recall	0.87	0.87	0.53	**0.94**	0.89	0.78	0.76	0.88	0.88
KC score	0.86	0.85	0.52	**0.90**	0.88	0.76	0.75	0.86	0.86
RSEM-EVAL score ($\times 10^9$)	-7.89	-7.87	-19.41	-7.59	**-6.70**	-11.54	-12.46	-7.94	-7.52
**Transrate comparative metrics**									
Contig score	0.12	0.13	0.25	0.01	0.11	0.07	**0.46**	0.15	0.05
P bases uncovered	0.30	0.27	**0.01**	0.67	0.23	0.09	**0.01**	0.22	0.47
Contigs segmented	14885	**19210**	24031	35601	23591	32939	19951	30833	28469
P references with CRBB	0.18	0.19	0.22	0.26	0.21	0.19	0.20	**0.27**	0.25
Reference coverage	0.20	0.20	0.15	**0.28**	0.21	0.15	0.16	0.21	0.24
50% covered	15363	15433	13613	**23311**	17031	12349	13542	17972	20390
95% covered	10778	10523	3861	**16648**	11396	5710	5737	10605	12723

Table S5: Benchmarking of **BinPacker**, **Bridger**, **IDBA**-tran, RNA-**Bloom**, **rnaSPAdes**, **SOAP**denovo-Trans, **SPAdes**, Trans-**ABySS** and **Trinity** on Mouse RNA-seq dataset. The annotated transcriptome of *M. musculus* GRCm38.75 consists of 38924 genes and 94545 isoforms. All contigs shorter than 200 bp were filtered out prior to the analysis. The best values for each metric are highlighted with bold.

Assembler	BinPacker	Bridger	IDBA	Bloom	rnaSPAdes	SOAP	SPAdes	ABySS	Trinity
Options							`--sc`		
K	25	25	20-60	25	33, 49	23	21, 33, 55	32	25
**rnaQUAST metrics**									
Transcripts	27234	42029	38313	46440	41975	31878	42949	36488	47746
Misassemblies	947	923	387	732	306	**37**	497	194	459
Duplication ratio	1.12	1.09	**1.00**	1.33	1.03	**1.00**	**1.00**	1.09	1.15
Database coverage, %	14.4	16.3	16.9	13.8	17.8	15.1	17.7	16.2	**18.2**
50%-assembled genes	6005	6090	6558	4859	**7001**	6241	6890	6321	6633
95%-assembled genes	1917	1909	1602	1256	**2494**	1653	2450	1798	2272
50%-assembled isoforms	6360	6451	6790	5591	7309	6376	7053	6931	**7386**
95%-assembled isoforms	1992	1982	1602	1346	**2536**	1655	2450	1850	2406
**Detonate scores**									
Nucleotide precision	0.76	0.76	0.86	0.59	0.80	**0.89**	0.82	0.79	0.7
Nucleotide recall	0.69	0.77	0.75	0.59	0.78	0.68	**0.79**	0.71	**0.79**
Nucleotide F1	0.73	0.76	0.8	0.59	0.79	0.77	**0.81**	0.75	0.74
Contig precision	0.204	0.163	0.174	0.1	0.188	0.22	0.164	**0.218**	0.159
Contig recall	0.052	0.064	0.062	0.043	0.074	0.066	0.066	**0.075**	0.071
Contig F1	0.083	0.092	0.092	0.06	0.106	0.101	0.094	**0.111**	0.098
k-mer recall	0.88	0.89	0.34	**0.92**	0.89	0.67	0.6	0.88	0.79
KC score	0.85	0.85	0.31	**0.89**	0.86	0.65	0.57	0.85	0.75
RSEM-EVAL score ($\times 10^9$)	-1.02	-1.02	-2.31	-1.1	**-0.93**	-1.46	-1.7	-1.03	-1.08
**Transrate comparative metrics**									
Contig score	0.23	0.25	0.19	0.05	0.20	0.37	**0.41**	0.38	0.14
P bases uncovered	0.18	0.14	**0.01**	0.33	0.06	0.02	**0.01**	0.13	0.21
Contigs segmented	**904**	1319	1383	3257	1366	1099	991	1798	1961
P references with CRBB	0.18	0.21	0.21	0.21	0.21	0.19	0.21	0.21	**0.22**
Reference coverage	0.17	**0.18**	0.17	0.14	**0.18**	0.15	**0.18**	0.17	**0.18**
50% covered	9125	9606	9331	7988	9630	8377	9537	9310	**10112**
95% covered	6292	6344	5511	4756	6608	5063	6414	6115	**6710**

Table S6: Benchmarking of **BinPacker**, **Bridger**, **IDBA**-tran, RNA-**Bloom**, **rnaSPAdes**, **SOAP**denovo-Trans, **SPAdes**, Trans-**ABySS** and **Trinity** on Worm RNA-seq dataset. The annotated transcriptome of *C. elegans* WBcel235.82 consists of 46748 genes and 57834 isoforms. All contigs shorter than 200 bp were filtered out prior to the analysis. The best values for each metric are highlighted with bold.

Assembler	BinPacker	Bridger	IDBA	Bloom	rnaSPAdes	SOAP	SPAdes	ABySS	Trinity
Options							`--sc`		
K	25	25	20-60	25	29, 45	23	21, 33, 55	32	25
**rnaQUAST metrics**									
Transcripts	20460	22147	23172	29995	23654	18801	21235	46145	24428
Misassemblies	294	256	40	96	105	**28**	130	37	217
Duplication ratio	1.20	1.14	1.01	1.59	1.11	1.01	**1.00**	1.68	1.17
Database coverage, %	33.3	33.7	33.2	**37.9**	36.0	32.1	33.3	37.7	36.1
50%-assembled genes	9907	9950	10075	10028	**10650**	9867	10378	10130	10394
95%-assembled genes	5666	5654	3786	5390	**6306**	5155	5948	5413	5682
50%-assembled isoforms	10398	10352	10227	**11639**	11197	9995	10426	11058	11127
95%-assembled isoforms	5910	5874	3788	6100	**6541**	5198	5949	5487	6030
**Detonate scores**									
Nucleotide precision	0.69	0.72	0.86	0.48	0.72	**0.87**	0.84	0.47	0.68
Nucleotide recall	0.86	0.88	0.85	0.86	**0.89**	0.84	0.87	0.87	0.88
Nucleotide F1	0.77	0.79	**0.86**	0.62	0.80	0.85	**0.86**	0.61	0.77
Contig precision	0.289	0.269	0.205	0.248	0.258	**0.331**	0.27	0.148	0.284
Contig recall	0.151	0.152	0.121	0.19	0.156	0.159	0.146	0.175	**0.177**
Contig F1	0.198	0.195	0.153	0.215	0.195	0.215	0.19	0.161	**0.218**
k-mer recall	0.96	0.96	0.67	**0.99**	0.97	0.91	0.96	0.96	0.96
KC score	0.95	0.95	0.67	**0.98**	0.96	0.9	0.96	0.96	0.95
RSEM-EVAL score ($\times 10^9$)	-1.56	-1.56	-5.24	**-1.31**	-1.41	-2.34	-1.62	-1.49	-1.44
**Transrate comparative metrics**									
Contig score	0.26	0.34	0.34	0.06	0.37	0.50	**0.61**	0.05	0.29
P bases uncovered	0.26	0.19	**0.00**	0.51	0.15	0.02	**0.00**	0.51	0.25
Contigs segmented	3067	3301	4215	4497	3640	**2886**	2978	5035	3875
P references with CRBB	0.39	0.41	0.41	**0.44**	0.43	0.39	0.41	**0.44**	0.43
Reference coverage	0.34	0.35	0.32	**0.38**	0.35	0.30	0.33	0.36	0.36
50% covered	10822	10908	10643	**11638**	11391	9937	10668	11270	11446
95% covered	8615	8589	6480	**9084**	8885	7004	8015	8355	9039

Table S7: Benchmarking of **BinPacker**, **IDBA**-tran, RNA-**Bloom**, **rnaSPAdes**, **SOAP**denovo-Trans, **SPAdes**, Trans-**ABySS** and **Trinity** on Corn SS dataset. Assemblers having strand-specific mode were run with the corresponding options. The annotated transcriptome of *Z. mays* AGPv3.29 consists of 39479 genes and 63230 isoforms. All contigs shorter than 200 bp were filtered out prior to the analysis. The best values for each metric are highlighted with bold. *Note, that Bridger failed to assemble this dataset.*

Assembler	BinPacker	IDBA	Bloom	rnaSPAdes	SOAP	SPAdes	ABySS	Trinity
Options						`--sc`		
K	25	20-60	25	33, 49	23	21, 33, 55	32	25
**rnaQUAST metrics in normal mode**								
Transcripts	20981	123286	152794	99255	191089	96929	139985	150288
Misassemblies	3833	**303**	4313	2534	430	1481	794	3301
Duplication ratio	1.92	1.06	2.94	1.49	1.07	**1.04**	1.48	2.08
Database coverage, %	11.7	32.2	**38.7**	35.6	30.3	31	35.2	37.3
50%-assembled genes	5397	9005	14644	**15374**	10322	12946	12918	14605
95%-assembled genes	2204	840	4617	**4999**	2456	3150	3306	4457
50%-assembled isoforms	6106	9431	**19412**	17789	10726	13356	14630	18139
95%-assembled isoforms	2394	840	**5460**	5377	2489	3161	3405	4998
**rnaQUAST metrics in strand-specific mode**								
Database coverage, %	11.7	16.4	**38.2**	35.1	15.4	15.9	34.7	36.8
50%-assembled genes	5368	4603	14457	**15128**	5220	6687	12701	14373
95%-assembled genes	2189	454	4543	**4915**	1260	1612	3246	4381
50%-assembled isoforms	6077	4713	**19201**	17542	5376	6801	14401	17890
95%-assembled isoforms	2379	454	**5384**	5290	1273	1615	3343	4921
**Detonate scores**								
Nucleotide precision	0.32	**0.40**	0.15	0.29	0.39	**0.40**	0.32	0.23
Nucleotide recall	0.44	0.77	0.87	0.87	0.72	0.80	0.82	**0.88**
Nucleotide F1	0.37	0.53	0.26	0.44	0.51	**0.53**	0.46	0.36
Contig precision	**0.087**	0.026	0.053	0.043	0.027	0.027	0.048	0.046
Contig recall	0.020	0.034	**0.088**	0.046	0.056	0.029	0.072	0.074
Contig F1	0.032	0.029	**0.066**	0.044	0.036	0.028	0.057	0.056
k-mer recall	0.61	0.55	**0.78**	0.71	0.51	0.66	0.69	0.70
KC score	0.59	0.53	**0.72**	0.68	0.49	0.64	0.67	0.66
RSEM-EVAL score ($\times 10^9$)	-2.80	-5.72	**-1.64**	-1.86	-4.91	-2.86	-2.22	-1.96
**Transrate comparative metrics**								
Contig score	0.027	0.196	0.015	0.142	0.036	**0.388**	0.109	0.061
P bases uncovered	0.479	**0.015**	0.637	0.291	0.097	0.019	0.222	0.481
Contigs segmented	**3870**	13678	13865	9431	16305	7347	14029	10701
P references with CRBB	0.076	0.247	0.273	0.222	0.220	0.213	**0.284**	0.264
Reference coverage	0.066	0.104	**0.198**	0.149	0.088	0.107	0.144	0.174
50% covered	8531	17537	**27336**	21258	12476	16768	21278	24849
95% covered	5844	4361	**14672**	11504	4709	6547	9848	12921

Table S8: Benchmarking of **BinPacker**, **Bridger**, **IDBA**-tran, RNA-**Bloom**, **rnaSPAdes**, **SOAP**denovo-Trans, **SPAdes**, Trans-**ABySS** and **Trinity** on Arabidopsis SS dataset. Assemblers having strand-specific mode were run with the corresponding options. The annotated transcriptome of *A. thaliana* TAIR10.29 consists of 31496 genes and 40646 isoforms. All contigs shorter than 200 bp were filtered out prior to the analysis. The best values for each metric are highlighted with bold.

Assembler	BinPacker	Bridger	IDBA	Bloom	rnaSPAdes	SOAP	SPAdes	ABySS	Trinity
Options							`--sc`		
K	25	25	20-60	25	43, 63	23	21, 33, 55	32	25
**rnaQUAST metrics in normal mode**									
Transcripts	82756	89058	72148	148393	61951	151437	63846	114944	118184
Misassemblies	5490	5368	205	2078	567	**182**	632	418	2296
Duplication ratio	1.82	1.72	**1.05**	4.00	1.74	1.14	**1.05**	1.53	2.62
Database coverage, %	49.8	50.5	51.8	**63.8**	57	52.2	51.1	56.8	61
50%-assembled genes	16871	16928	16454	**20606**	20187	17450	17633	19140	20439
95%-assembled genes	12242	12211	4455	**16783**	16217	8925	11267	10891	15580
50%-assembled isoforms	17969	18019	16490	**24061**	21687	17687	17670	19532	22901
95%-assembled isoforms	12876	12795	4455	**18700**	16959	8983	11268	10898	16763
**rnaQUAST metrics in strand-specific mode**									
Database coverage, %	45.4	45.7	26.6	**62.1**	56	27.4	29	54.2	60
50%-assembled genes	16460	16507	8523	**20473**	19894	9244	9936	18924	20281
95%-assembled genes	12208	12175	2360	**16774**	16103	4858	6347	10846	15556
50%-assembled isoforms	17391	17427	8526	**23832**	21400	9369	9950	19161	22718
95%-assembled isoforms	12844	12761	2360	**18694**	16855	4888	6348	10852	16743
**Detonate scores**									
Nucleotide precision	0.37	0.37	**0.67**	0.17	0.41	0.53	0.64	0.47	0.26
Nucleotide recall	0.94	0.95	0.88	**0.97**	0.96	0.88	0.91	0.92	**0.97**
Nucleotide F1	0.53	0.54	**0.76**	0.29	0.58	0.66	0.75	0.62	0.41
Contig precision	0.07	0.06	0.03	0.07	**0.11**	0.04	0.05	0.07	0.07
Contig recall	0.13	0.13	0.06	**0.26**	0.16	0.16	0.08	0.20	0.20
Contig F1	0.087	0.082	0.041	0.114	**0.132**	0.070	0.063	0.107	0.104
k-mer recall	0.92	0.92	0.58	**0.99**	0.94	0.73	0.82	0.92	0.92
KC score	0.92	0.92	0.58	**0.98**	0.94	0.72	0.81	0.92	0.91
RSEM-EVAL score ($\times 10^9$)	-12.19	-12.20	-30.31	**-9.77**	-11.05	-20.56	-18.90	-12.81	-12.39
**Transrate comparative metrics**									
Contig score	0.030	0.029	0.062	0.004	0.063	0.023	**0.126**	0.045	0.017
P bases uncovered	0.28	0.25	0.03	0.60	0.20	0.10	**0.03**	0.14	0.48
Contigs segmented	17238	18195	21694	20102	**14615**	17124	14733	22702	20610
P references with CRBB	0.51	0.52	0.48	**0.58**	0.50	0.46	0.47	0.56	0.57
Reference coverage	0.45	0.45	0.36	**0.54**	0.46	0.33	0.37	0.44	0.51
50% covered	21423	21356	18333	**25458**	21925	16606	18305	21120	24321
95% covered	17723	17387	8992	**22247**	18765	9381	12150	16235	20380

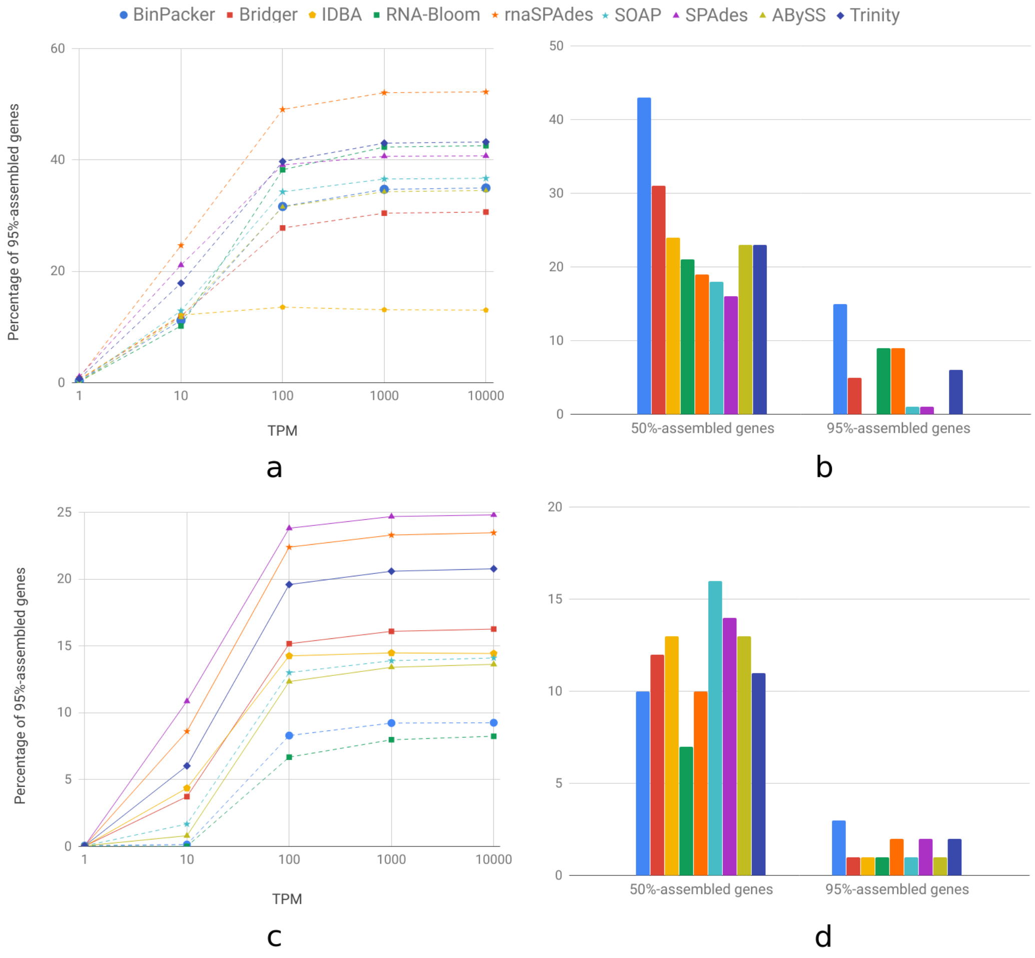

Figure S1: (a, c) Cumulative plot showing how fraction of 95%-assembled genes in each assembly depends on the gene coverage by reads in TPM (Transcripts Per Kilobase Million) reported by RSEM simulator; (b, d) Number of 50%/95%-assembled genes in each assembly that have zero reads generated by RSEM simulator (i.e. falsely assembled genes). Plots (a, b) are constructed for assemblies obtained from Human simulated data, (c, d) represent Mouse simulated dataset.

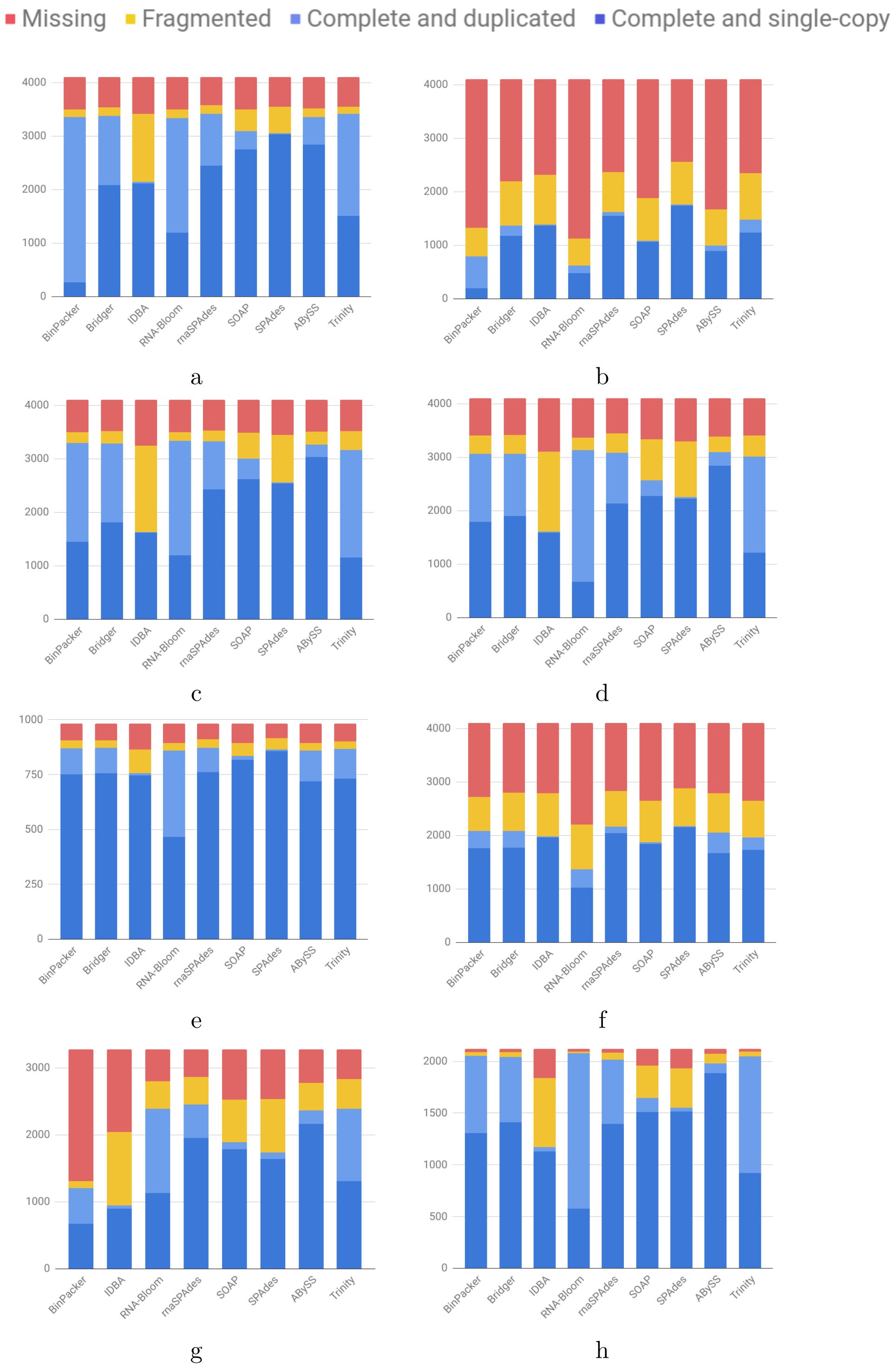

Figure S2: BUSCO results for (a) Human simulated, (b) Mouse simulated, (c) Human, (d) Human large, (e) Mouse, (f) Worm, (g) Corn SS and (h) Arabidopsis SS assemblies. Dark blue indicates complete and single-copy genes, light blue — complete and duplicated, yellow — fragmented and red corresponds to missing BUSCOs. The following BUSCO gene databases were used for the analysis: mammalian for all *H. sapiens* and *M. musculus* assemblies, nematoda for *C. elegans*, liliopsida for *Z. mays* and eudicotyledons for *A. thaliana*.

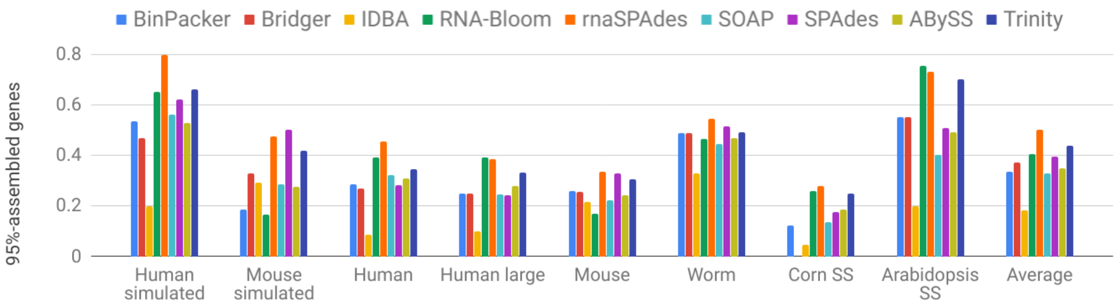

a

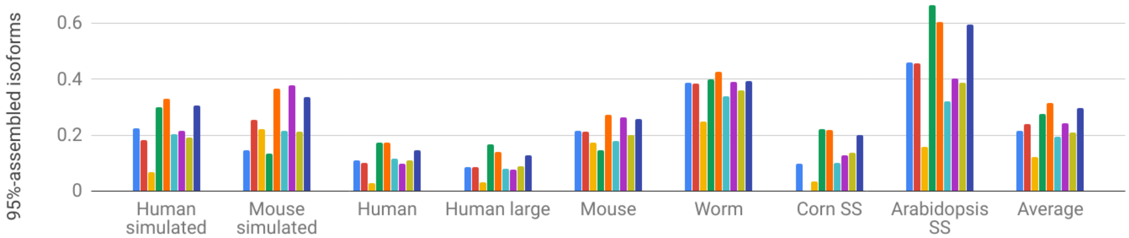

b

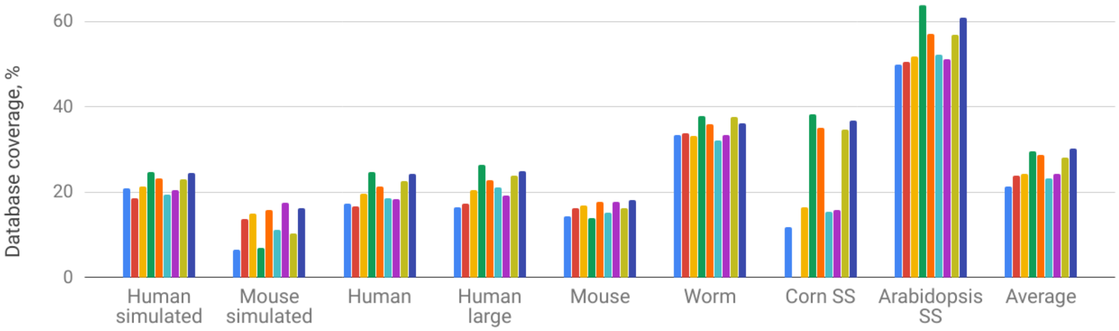

c

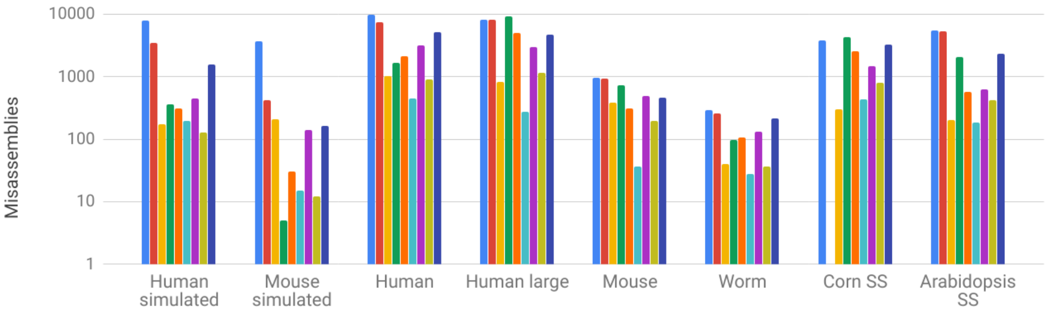

d

Figure S3: (a) Fraction of 95%-assembled genes, (b) fraction of 95%-assembled isoforms, (c) database coverage and (d) misassemblies reported by rnaQUAST drawn as bar plots for all generated assemblies. Fraction of assembled genes/isoforms is calculated relative to number of genes/isoforms reported by kallisto (Bray *et al.*, 2016) with per-nucleotide coverage > 5 (see Table S15 for details). Plot for number of misassemblies is given in logarithmic scale. The last columns show average values over all datasets (except for misassemblies).

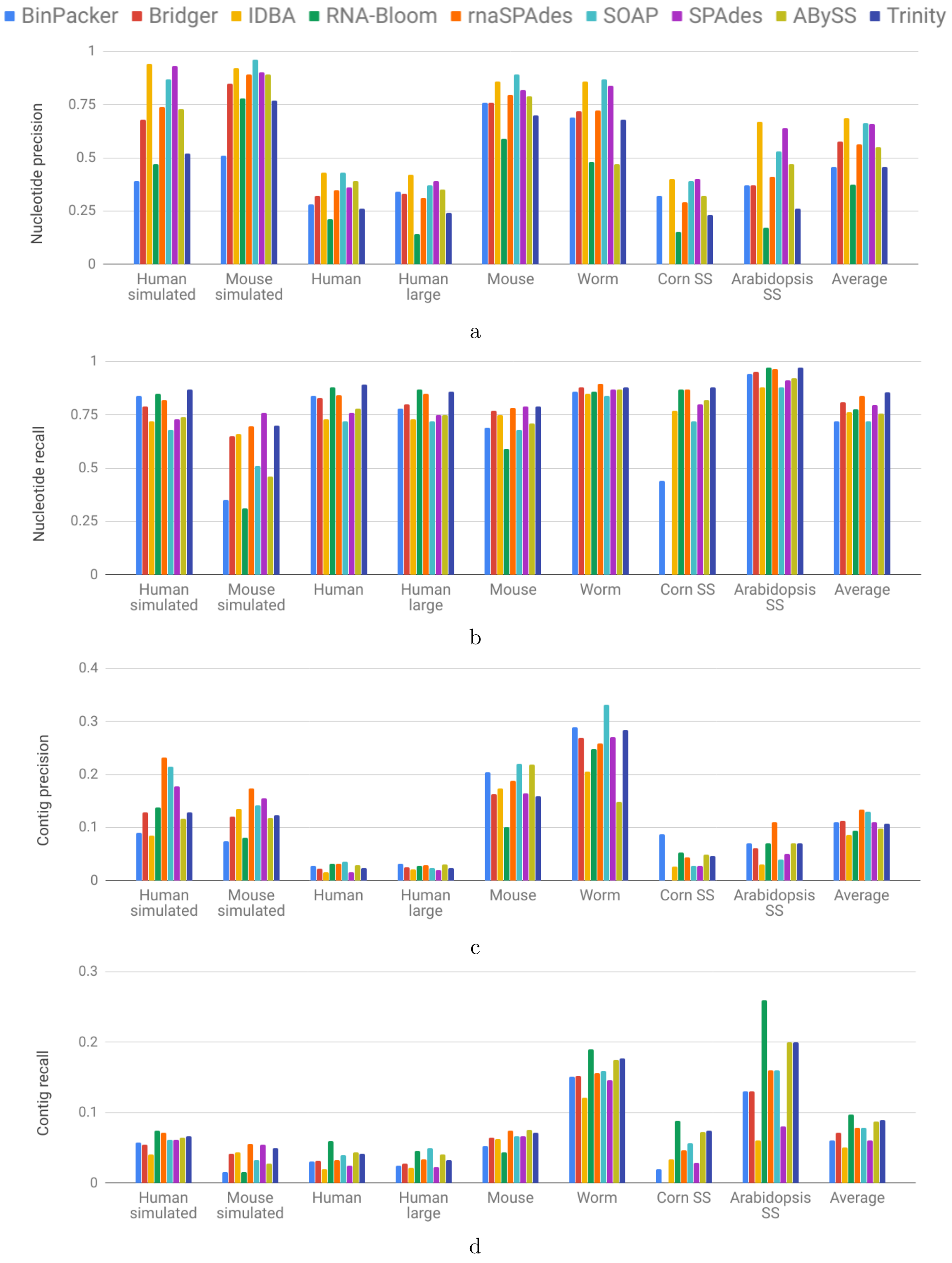

Figure S4: (a) Nucleotide precision, (b) nucleotide recall, (c) contig precision, and (d) contig recall reported by DETONATE REF-EVAL drawn as bar plots for all generated assemblies. The last columns show average values over all datasets.

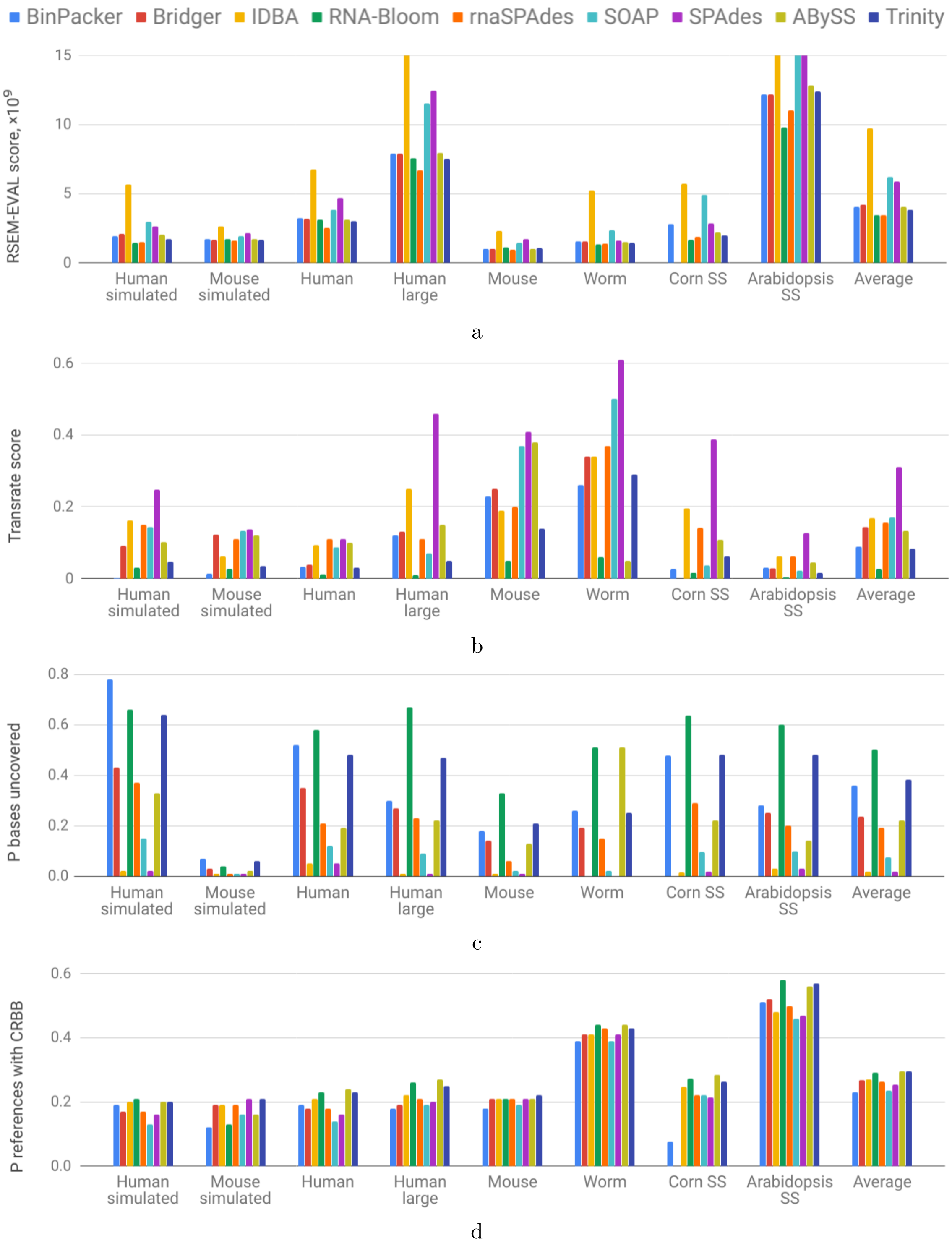

Figure S5: (a) Detonate RSEM-EVAL score, (b) Transrate score, (c) fraction of uncovered bases and (d) fraction of reference sequences covered by CBBR hits reported by Transrate drawn as bar plots for all generated assemblies. The last columns show average values over all datasets. For better visibility, the absolute value of original RSEM-EVAL score was taken, thus, the lower the value, the better the assembly according to this metric. Note, that some bars were cut to the maximal Y axis value.

Table S9: rnaSPAdes performance with different k-mer sizes on Worm dataset (90 bp long paired-end reads). Upper part of the table contains results for single-k runs. Lower part shows rnaSPAdes performance for different lower k values with fixed upper one (45). Default k-mer sizes and the best values for each metric are highlighted with bold. *Note, that these experiments were performed during rnaSPAdes development and thus results may not be exactly the same as in comparison tables.*

**Single k-mer size**													
K value	21	25	29	33	37	41	**45**	49	53	57	61	65	69
Transcripts	32626	33207	33133	33402	33598	33728	34015	34594	35567	36850	38803	41401	45218
Misassemblies	572	233	180	146	114	88	86	84	72	**54**	63	79	77
Database coverage, %	35	36.6	36.7	**37**	**37**	36.8	36.7	36.5	36.4	36	35.8	35.6	35.4
50%-assembled genes	10142	10664	10752	10821	**10854**	10833	10778	10752	10713	10637	10537	10422	10218
95%-assembled genes	5627	6260	6354	**6406**	6403	6389	6338	6194	6062	5880	5765	5601	5405
**Iterative run with fixed upper k-mer size 45**													
Lower k-mer size	21	25	**29**	33	37	41							
Transcripts	32936	33190	33259	33461	33583	33755							
Misassemblies	177	108	94	102	100	**93**							
Database coverage, %	36.5	36.8	36.8	**36.9**	36.8	36.8							
50%-assembled genes	10792	10870	**10887**	10884	10855	10832							
95%-assembled genes	6382	6441	**6446**	6438	6405	6388							

Table S10: rnaSPAdes performance with different k-mer sizes on Mouse dataset (101 bp long paired-end reads). Upper part of the table contains results for single-k runs. Lower part shows rnaSPAdes performance for different lower k values with fixed upper one (49). Default k-mer sizes and the best values for each metric are highlighted with bold.*Note, that these experiments were performed during rnaSPAdes development and thus results may not be exactly the same as in comparison tables.*

**Single k-mer size**													
K value	21	25	29	33	37	41	45	**49**	53	57	61	65	69
Transcripts	62702	68785	71583	72341	73263	74624	76692	78965	81989	86209	92204	99998	110489
Misassemblies	1443	634	397	328	249	220	205	**173**	220	247	362	516	664
Database coverage, %	17.4	19.1	19.6	**19.7**	19.6	19.6	19.5	19.4	19.3	19.1	18.9	18.6	18.3
50%-assembled genes	6301	6904	7023	**7068**	7029	6968	6890	6737	6568	6334	6029	5634	5180
95%-assembled genes	1808	2324	2495	**2517**	2496	2477	2383	2309	2200	2071	1941	1708	1507
**Iterative run with fixed upper k-mer size 49**													
Lower k-mer size	21	25	29	**33**	37	41	45						
Transcripts	70814	71625	72355	73185	74161	75399	76977						
Misassemblies	443	332	284	250	231	229	**206**						
Database coverage, %	19.5	**19.8**	**19.8**	19.7	19.7	19.6	19.5						
50%-assembled genes	7245	**7273**	7217	7142	7074	6992	6890						
95%-assembled genes	2667	**2681**	2660	2593	2537	2472	2393						

Table S11: rnaSPAdes performance with different k-mer sizes on Human dataset (150 bp long paired-end reads). Upper part of the table contains results for single-k runs. Lower part shows rnaSPAdes performance for different lower k values with fixed upper one (73). Default k-mer sizes and the best values for each metric are highlighted with bold. *Note, that these experiments were performed during rnaSPAdes development and thus results may not be exactly the same as in comparison tables.*

**Single k-mer size**											
K value		31	37	43	49	55	61	67	**73**	79	85
Transcripts		210121	212907	214312	213817	215063	216767	219770	223354	229623	237872
Misassemblies		4720	4347	3804	3403	3032	2624	2369	**2145**	2284	2732
Database coverage, %		20.7	21	**21.1**	21	20.8	20.9	20.7	20.8	20.7	20.6
50%-assembled genes		12712	12945	13120	13177	13239	13350	13353	**13368**	13289	13204
95%-assembled genes		5107	5420	5679	5859	6067	6230	6255	**6317**	6212	6021
**Iterative run with fixed upper k-mer size 73**											
Lower k-mer size	25	31	37	43	**49**	55	61	67			
Transcripts	225165	224457	222862	221591	221268	220526	220554	221764			
Misassemblies	2771	2841	2981	2869	2709	2679	2462	**2314**			
Database coverage, %	**20.9**	**20.9**	20.8	20.8	20.8	20.8	20.8	20.8			
50%-assembled genes	13459	**13498**	13454	13463	13468	13445	13469	13427			
95%-assembled genes	**6376**	6372	6368	6326	6333	6302	6307	6288			

Table S12: Comparison between simplification procedures of SPAdes and rnaSPAdes on Mouse, Worm and Human datasets. The table shows the number of erroneous/correct edges and k-mers in the initial de Bruijn graph and the final assembly graphs constructed by SPAdes and rnaSPAdes. A k-mer is considered to be correct if it has exact match to a reference transcript. The edge is defined as erroneous if it has less than a half non-reference k-mers.

The table demonstrates that rnaSPAdes simplification algorithms typically preserve more reference k-mers, which allow to restore more isoforms. At the same time, in some cases it also keeps more erroneous k-mers in the graph. However, a major fraction of these k-mers are contained in short isolated edges, which are removed by SPAdes simplification algorithms, but preserved by rnaSPAdes. However, in rnaSPAdes such edges are removed later during the path filtration step and thus do not affect the assembly quality.

	Initial graph	SPAdes simplification	rnaSPAdes simplification	Difference
M. musculus				
Correct k-mers	31322711	30669080	30734404	65324
Correct edges	1578840	173086	171635	-1451
Erroneous k-mers	40617576	15727283	15495169	-232114
Erroneous edges	3080748	269288	260604	-8684
C. elegans				
Correct k-mers	19083410	18749538	18792620	43082
Correct edges	1587285	52932	54133	1201
Erroneous k-mers	19635777	4855550	4893985	38435
Erroneous edges	2628040	67948	68326	378
H. sapiens				
Correct k-mers	59669678	55575499	55647509	72010
Correct edges	2807476	125508	122740	-2768
Erroneous k-mers	236599327	143949729	143970896	21167
Erroneous edges	7322492	1205761	1204244	-1517

Table S13: Exact parameters for filtering constructed paths. The path is removed if it satisfies one of the following conditions: (i) the path is shorter than *min_len*, (ii) the path is shorted than $relative\_min\_len \cdot read\_length$, (iii) the path has coverage lower than *min_cov* and is shorter than *cov_min_len* or $cov\_rel\_min\_len \cdot read\_length$, (iii) the path contains a single isolated edge, which has coverage lower than *iso_min_cov* and is shorter than *iso_min_len* or $iso\_rel\_min\_len \cdot read\_length$.

	Parameter value		
Filtration level	Soft	Normal	Hard
min_length	95	110	130
relative_min_len	1.05	1.3	1.5
min_cov	1	2	3
cov_min_len	130	140	180
cov_rel_min_len	1.5	1.6	2.0
iso_min_cov	2	4	8
iso_min_len	100	130	180
iso_rel_min_len	1.2	1.5	2.0

Table S14: Comparison of different rnaSPAdes filtration levels on Human and Arabidopsis SS data. Relaxed filtration parameters result in higher database coverage and larger number of 50%/95%-assembled genes/isoforms, but at the same time increases the number of misassembled sequences and the total amount of assembled contigs.

Dataset		Human			Arabidopsis SS	
Filtration level	Soft	Normal	Hard	Soft	Normal	Hard
Transcripts	215997	167884	120219	95004	65063	54291
Misassemblies	2209	2111	**2033**	610	571	**562**
Database coverage, %	**0.217**	0.213	0.208	**0.576**	0.571	0.566
50%-assembled genes	**13467**	13377	13218	**20223**	20190	20135
95%-assembled genes	**7112**	7094	7064	**16224**	16217	16205
50%-assembled isoforms	**18736**	18619	18392	**21723**	21690	21635
95%-assembled isoforms	**8044**	8026	7996	**16966**	16959	16947

Table S15: Amount of genes and isoforms detected by kallisto (Bray *et al.*, 2016) that have nucleotide coverage higher than respective threshold. Per-base coverage value for each gene/isoform was estimated as $2 \times RL \times C/GL$, where RL is read length, GL is gene/isoform length and C is the fragment counts reported by kallisto (for paired-end reads each fragment contains two reads).

Coverage cut-off	2	3	4	5	10	20	30
**Genes**							
Human simulated	15969	14936	14216	13700	12121	10523	9359
Mouse simulated	11072	9776	8732	7828	4996	2751	1832
Human	19875	17911	16601	15650	13009	10711	9261
Human large	23880	21709	20212	19071	15916	13132	11693
Mouse	10641	9373	8337	7450	4692	2585	1706
Worm	13600	12650	12047	11577	10253	8962	8172
Corn SS	21331	19877	18764	17869	14996	11681	9617
Arabidopsis SS	23264	22825	22483	22223	21360	20378	19643
**Isoforms**							
Human simulated	55182	48844	44061	40401	29702	20800	16332
Mouse simulated	17334	14150	11989	10334	6017	3159	2081
Human	66806	57677	51269	46205	32235	21283	16132
Human large	82165	73504	67295	62521	48376	35429	28816
Mouse	15788	12746	10762	9318	5470	2891	1877
Worm	18131	16810	15943	15268	13294	11344	10130
Corn SS	31907	28879	26611	24805	19085	13641	10859
Arabidopsis SS	29653	29008	28482	28063	26538	24687	23358

References

Andrews, S. *et al.* (2010). Fastqc: a quality control tool for high throughput sequence data.

Bolger, A. M., Lohse, M., and Usadel, B. (2014). Trimmomatic: a flexible trimmer for illumina sequence data. *Bioinformatics*, **30**(15), 2114–2120.

Bray, N. L., Pimentel, H., Melsted, P., and Pachter, L. (2016). Near-optimal probabilistic rna-seq quantification. *Nature biotechnology*, **34**(5), 525.

Bushmanova, E. *et al.* (2016). rnaQUAST: a quality assessment tool for de novo transcriptome assemblies. *Bioinformatics*, **32**(14), 2210–2212.

Camacho, C. *et al.* (2009). Blast+: architecture and applications. *BMC bioinformatics*, **10**(1), 421.

Chang, Z., Li, G., Liu, J., Zhang, Y., Ashby, C., Liu, D., Cramer, C. L., and Huang, X. (2015). Bridger: a new framework for de novo transcriptome assembly using rna-seq data. *Genome biology*, **16**(1), 30.

Grabherr, M. G. *et al.* (2011). Full-length transcriptome assembly from RNA-Seq data without a reference genome. *Nat. Biotechnol.*, **29**(7), 644–652.

Kent, W. J. (2002). BLAT–the BLAST-like alignment tool. *Genome Res.*, **12**(4), 656–664.

Langmead, B. and Salzberg, S. (2012). Fast gapped-read alignment with bowtie 2. *Nature Methods*, **9**, 357–359.

Li, B. *et al.* (2014). Evaluation of de novo transcriptome assemblies from RNA-Seq data. *Genome Biol.*, **15**(12), 553.

Liu, J., Li, G., Chang, Z., Yu, T., Liu, B., McMullen, R., Chen, P., and Huang, X. (2016). Binpacker: packing-based de novo transcriptome assembly from rna-seq data. *PLoS computational biology*, **12**(2), e1004772.

Marçais, G. and Kingsford, C. (2011). A fast, lock-free approach for efficient parallel counting of occurrences of k-mers. *Bioinformatics*, **27**(6), 764–770.

Nip, K. M. (2017). *RNA-Bloom: de novo RNA-seq assembly with Bloom filters*. Ph.D. thesis, University of British Columbia.

Patro, R., Duggal, G., Love, M. I., Irizarry, R. A., and Kingsford, C. (2017). Salmon provides fast and bias-aware quantification of transcript expression. *Nature methods*, **14**(4), 417.

Peng, Y. *et al.* (2013). IDBA-tran: a more robust de novo de bruijn graph assembler for transcriptomes with uneven expression levels. *Bioinformatics*, **29**(13), i326–i334.

Robertson, G. *et al.* (2010). De novo assembly and analysis of RNA-seq data. *Nat. Methods*, **7**(11), 909–912.

Simão, F. A. *et al.* (2015). BUSCO: assessing genome assembly and annotation completeness with single-copy orthologs. *Bioinformatics*, **31**(19), 3210–3212.

Smith-Unna, R. *et al.* (2016). TransRate: reference-free quality assessment of de novo transcriptome assemblies. *Genome research*, **2**(8), 1134–1144.

Tang, S., Lomsadze, A., and Borodovsky, M. (2015). Identification of protein coding regions in rna transcripts. *Nucleic acids research*, **43**(12), e78–e78.

Wu, T. D. and Watanabe, C. K. (2005). GMAP: a genomic mapping and alignment program for mRNA and EST sequences. *Bioinformatics*, **21**(9), 1859–1875.

Xie, Y. *et al.* (2014). SOAPdenovo-Trans: De novo transcriptome assembly with short rna-seq reads. *Bioinformatics*, page btu077.

Zaharia, M., Bolosky, W. J., Curtis, K., Fox, A., Patterson, D., Shenker, S., Stoica, I., Karp, R. M., and Sittler, T. (2011). Faster and more accurate sequence alignment with snap. *arXiv preprint arXiv:1111.5572*.
