# Supplementary material for: rnaSPAdes: a de novo transcriptome assembler and its application to RNA-Seq data
Source: Gigascience. 2019 Sep 3;8(9):giz100. doi: 10.1093/gigascience/giz100 (PMC6736328; doi:10.1093/gigascience/giz100)
Supplement: giz100_Response_to_Reviewer_Comments_Original_Submission [file giz100_response_to_reviewer_comments_original_submission.pdf]

Author's Response To Reviewer Comments

Close

Attached as PDF

Close
