# Supplementary material for: rnaSPAdes: a de novo transcriptome assembler and its application to RNA-Seq data
Source: Gigascience. 2019 Sep 3;8(9):giz100. doi: 10.1093/gigascience/giz100 (PMC6736328; doi:10.1093/gigascience/giz100)
Supplement: giz100_Response_to_Reviewer_Comments_Revision_1 [file giz100_response_to_reviewer_comments_revision_1.pdf]

Author's Response To Reviewer Comments

Close

Dear Editor

Thanks a lot for the positive feedback! Although you suggested not to carry out any further analyses, we decided to run several assemblers with k-mer sizes provided by rnaSPAdes (as suggested by Reviewer 3). Result for different assemblers are rather inconsistent (e.g. in some cases the default k is better, in some it is not). Even in cases when an assembly is improved, it still does not outperform rnaSPAdes.

We have added a paragraph regarding k-mer size selection in the beginning of the Analyses section. Detailed response to the Reviewer's comments are attached.

Best regards
Elena Bushmanova and Andrey Prjibelski

Close
