# Supplementary material for: rnaSPAdes: a de novo transcriptome assembler and its application to RNA-Seq data
Source: Gigascience. 2019 Sep 3;8(9):giz100. doi: 10.1093/gigascience/giz100 (PMC6736328; doi:10.1093/gigascience/giz100)
Supplement: giz100_Response_to_Reviewer_Comments_Revision_2 [file giz100_response_to_reviewer_comments_revision_2.pdf]

Author's Response To Reviewer Comments

Close

Dear Hans Zauner,

We have added the reference to GigaDB dataset and cited it in data availability section as instructed.

Best regards
Andrey

Close
