# Supplementary material for: rnaSPAdes: a de novo transcriptome assembler and its application to RNA-Seq data
Source: Gigascience. 2019 Sep 3;8(9):giz100. doi: 10.1093/gigascience/giz100 (PMC6736328; doi:10.1093/gigascience/giz100)
Supplement: giz100_Reviewer_1_Report_Original_Submission — Camille Marchet -- 12/10/2018 Reviewed [file giz100_reviewer_1_report_original_submission.pdf]

**Reviewer Report**

**Title: rnaSPAdes: a de novo transcriptome assembler and its application to RNA-Seq data**

**Version: Original Submission** **Date:** 12/10/2018

**Reviewer name: Camille Marchet**

**Reviewer Comments to Author:**

Summary
This work presents algorithmic adaptations of the assembler Spades to process RNA-seq data and produce RNA transcript assembly.
Spades is already designed to work on read coverage skewed distributions since it was originally designed for single cell data. The authors identify and justify the necessary modifications to Spades pipeline in order to adapt it to RNA-seq specifics. They present results on several datasets (simulated and real) from various model species. They compare their pipeline to the main state of the art RNA-seq assemblers. The results are mainly assessed by a tool that was developed in the author's group as well. They also provide results from independent assessments in the supplementary.
The paper is well written, methods and results are overall well explained, and clear figures are provided.
Minor comments/questions
In the following, I will refer to positive/negative/neutral comments using "+", "-", "o".
* Methods
+ the graph cleaning step is very well described. It correctly identifies and addresses the specific issues that occur in DBG built on RNA.
+ RNAspades' pipeline benefits from an implementation that can be easily accessed, downloaded, installed and that provides results.
- The authors state that exon skipping is the most frequent alternative splicing event to justify their bubble crushing algorithm . However, alternative start/end of exons can be both short and biologically extremely meaningful. I think the lack of resolution for this type of events (though acceptable) should not be understated.
o Did the authors assess the impact of BayesHammer on their assembly? The tip removal described in the paper seems cautious and efficient, could you explain the importance of BayesHammer in addition to this step? The main pitfalls of BayesHammer's correction step are not well described.
o How marginal is the effect described in figure 5?
o Could you clarify exactly when the paired end information is used within the pipeline, and succinctly recall how it is included to the DBG
* Results
+ the authors are honest about the difficulty to select a "good" assembler and provide comprehensive benchmarks
- however, Spades seems to be a serious concurrent to RNAspades, in particular on real data, even when only referring to one of the metrics the authors pointed out as of major importance to assess assembly quality ( i.e. 95% assembled genes). Can you explain this difficulty to show that RNASpades outperforms

Spades on RNA ? In particular, how do you explain that all tendencies remain the same between human simulated and real datasets (figure 3) at the exception of Spades / RNASpades results ?
o Were real datasets reads filtered/trimmed prior to assembly ?
* Discussion/conclusion
- in potential impact, you should either show some results of your metatranscriptomics analysis (that can be in the supplementary data) or not mention it.
o I feel that the paragraph [Reports presented in this manuscript include large variety of metric … does not suit well for further reference-free analysis.] should be placed on top of the discussion. This would help to better apprehend the summary about each metric.
o In the conclusion I'd like to see clear points that demonstrate the advantage of RNASpades over its original pipeline Spades (see my comments in Results)
Finally, a remark on the supplementary data: is there an error in RNASpades's color bar on figures of the supplementary ? It seems it is dark blue instead of purple.

**Level of Interest**

Please indicate how interesting you found the manuscript: Choose an item.

**Quality of Written English**

Please indicate the quality of language in the manuscript: Choose an item.

**Declaration of Competing Interests**

Please complete a declaration of competing interests, considering the following questions:

- Have you in the past five years received reimbursements, fees, funding, or salary from an organisation that may in any way gain or lose financially from the publication of this manuscript, either now or in the future?
- Do you hold any stocks or shares in an organisation that may in any way gain or lose financially from the publication of this manuscript, either now or in the future?
- Do you hold or are you currently applying for any patents relating to the content of the manuscript?
- Have you received reimbursements, fees, funding, or salary from an organization that holds or has applied for patents relating to the content of the manuscript?
- Do you have any other financial competing interests?
- Do you have any non-financial competing interests in relation to this paper?

If you can answer no to all of the above, write 'I declare that I have no competing interests' below. If your reply is yes to any, please give details below.

I declare that I have no competing interests.

I agree to the open peer review policy of the journal. I understand that my name will be included on my report to the authors and, if the manuscript is accepted for publication, my named report including any attachments I upload will be posted on the website along with the authors' responses. I agree for my report to be made available under an Open Access Creative Commons CC-BY license (http://creativecommons.org/licenses/by/4.0/). I understand that any comments which I do not wish to be included in my named report can be included as confidential comments to the editors, which will not be published.

Choose an item.

To further support our reviewers, we have joined with Publons, where you can gain additional credit to further highlight your hard work (see: https://publons.com/journal/530/gigascience). On publication of this paper, your review will be automatically added to Publons, you can then choose whether or not to claim your Publons credit. I understand this statement.

Yes Choose an item.
