# Supplementary material for: rnaSPAdes: a de novo transcriptome assembler and its application to RNA-Seq data
Source: Gigascience. 2019 Sep 3;8(9):giz100. doi: 10.1093/gigascience/giz100 (PMC6736328; doi:10.1093/gigascience/giz100)
Supplement: giz100_Reviewer_2_Report_Original_Submission — Zhong Wang -- 12/17/2018 Reviewed [file giz100_reviewer_2_report_original_submission.pdf]

Reviewer Report

Title: rnaSPAdes: a de novo transcriptome assembler and its application to RNA-Seq data

Version: Original Submission **Date:** 12/17/2018

Reviewer name: Zhong Wang

Reviewer Comments to Author:

General comments:
Drawing a close analogy between single-cell genome assembly and transcriptome assembly, Bushmanova et al developed rnaSPAdes for de novo transcriptome assembly. The authors benchmarked their software on several synthetic and real-world datasets. They also compared the performance of rnaSPAdes with a few alternative assemblers by measuring several independent metrics. Based on these results, the authors conclude that rnaSPAdes is a "decent" transcriptome assembler, as it offers very good sensitivity, good specificity, and good computing efficiency. The analyses presented in this work are comprehensive, and their conclusion is sufficiently supported by the presented evidence. The paper is well written and easy to follow.
I have the following specific comments for minor revision:
1) Figure 3, the bars for Z. mayes are rather low. It should help reads to make a better comparison by using a different vertical scale than the rest, as the absolute total number of genes assembled here are not important.
2) The specific version numbers for each software tools should be noted
3) This could be optional: some further discussion of the misassemblies could be very informative. For example, one of these reasons may lead to misassemblies: a) paralogous genes b) complex splicing, or genes with many exons/isoforms c) Merge of overlapping genes because of non-strand-specific data is used

Level of Interest

Please indicate how interesting you found the manuscript: Choose an item.

Quality of Written English

Please indicate the quality of language in the manuscript: Choose an item.

Declaration of Competing Interests

Please complete a declaration of competing interests, considering the following questions:

- Have you in the past five years received reimbursements, fees, funding, or salary from an organisation that may in any way gain or lose financially from the publication of this manuscript, either now or in the future?

- Do you hold any stocks or shares in an organisation that may in any way gain or lose financially from the publication of this manuscript, either now or in the future?
- Do you hold or are you currently applying for any patents relating to the content of the manuscript?
- Have you received reimbursements, fees, funding, or salary from an organization that holds or has applied for patents relating to the content of the manuscript?
- Do you have any other financial competing interests?
- Do you have any non-financial competing interests in relation to this paper?

If you can answer no to all of the above, write 'I declare that I have no competing interests' below. If your reply is yes to any, please give details below.

I declare that I have no competing interests.

I agree to the open peer review policy of the journal. I understand that my name will be included on my report to the authors and, if the manuscript is accepted for publication, my named report including any attachments I upload will be posted on the website along with the authors' responses. I agree for my report to be made available under an Open Access Creative Commons CC-BY license (http://creativecommons.org/licenses/by/4.0/). I understand that any comments which I do not wish to be included in my named report can be included as confidential comments to the editors, which will not be published.

Choose an item.

To further support our reviewers, we have joined with Publons, where you can gain additional credit to further highlight your hard work (see: https://publons.com/journal/530/gigascience). On publication of this paper, your review will be automatically added to Publons, you can then choose whether or not to claim your Publons credit. I understand this statement.

Yes Choose an item.
