# Supplementary material for: rnaSPAdes: a de novo transcriptome assembler and its application to RNA-Seq data
Source: Gigascience. 2019 Sep 3;8(9):giz100. doi: 10.1093/gigascience/giz100 (PMC6736328; doi:10.1093/gigascience/giz100)
Supplement: giz100_Reviewer_3_Report_Original_Submission — Marcel Schulz -- 12/19/2018 Reviewed [file giz100_reviewer_3_report_original_submission.pdf]

Reviewer Report

Title: rnaSPAdes: a de novo transcriptome assembler and its application to RNA-Seq data

Version: Original Submission **Date:** 12/19/2018

Reviewer name: Marcel Schulz

Reviewer Comments to Author:

In the manuscript, Bushmanova et al have proposed an extension of the SPAdes genome assembler named "rnaSPAdes" and have shown parallels between rnaSPAdes and the SPAdes assembler in single cell mode (due to the fact that single cell sequencing gives rise to non-uniform coverage).
The authors have also compared rnaSPAdes to various other transcriptome assemblers. They have presented their results in the form of statistics obtained from assembly evaluation tools such as DETONATE, rnaQUAST and Transrate. I have the following concerns:
Major:
-     Overall it is hard to digest novel methodological contributions from the paper. One of the major modifications from their SPAdes genome assembler is the graph simplification process. Here the authors have removed the bubbles and tips present in the graph based on kmer coverage information, length of the tip/bubble and the sequence similarity between the tip/bubble and the alternate edge. This is similar to previous work, except for the fact maybe that tips are only removed if they have similar sequence, which is not done by other methods. But how large the effect due to this simple change is, remains unclear. The authors have also modified the path extension algorithm of the SPAdes assembler to allow for paths belonging to various isoforms, but the greedy algorithm is similar to other assemblers. They mention strategies to remove chimeric reads, but it is unclear what the impact of these removal strategies is. Overall, it is not clear whether methodological differences make for the improvement in their current experiments, due to the similarities to the other methods.
-     One other distinction to most other methods compared to in the manuscript is that rnaSPAdes includes an external error correction step inherited from SPAdes using Bayeshammer (which does not work on the de Bruijn graph). I am not sure whether any methodological change has been made to the BayesHammer approach in order to account for the specifics of RNA-seq data (its original purpose was single cell genomics data which shares the non-uniformity), but it has been shown several times before that error correction of RNA-seq data before assembly improves the contiguity of RNA assemblies. Tools like Rcorrector and SEECER that are made specifically for RNA-seq data, are likely to lead to a bigger boost than what is reported here (when one would compare the assembly result of any method after correcting the reads). And clearly any of these de novo correction methods can be used before the assembly with one of the assemblers tested here. For example, it would be interesting to see what difference it makes to assemble the Bayeshammer corrected reads with the competing methods, how does that compare to the results with rnaSPAdes?
-     The authors have compared rnaSPAdes against various other transcriptome assembler and have shown that rnaSPAdes performs sometimes better (in some statistics). The kmer parameter is one of the most important parameter in an assembly procedure. The

authors have optimized the kmer parameter for their own algorithm but have kept the default kmer parameter for other algorithms, which are completely different than the rnaSPAdes kmer often. Hence, the comparison is unfair as they are not made on similar grounds. Combined with the fact that there are no clear methodological improvements the results remain mostly inconclusive, except maybe the additional use of error correction is helpful as reported before.
-      All the datasets which the authors have used have very low coverage (less than 11 million for all but one dataset with 30 million). This is a bit strange as generation of high coverage datasets is quite common these days. Including at least one other high coverage dataset that is more standard right now would be important to judge the assembly performance as well as runtime and memory consumption. In terms of runtime and memory rnaSPAdes is neither particularly fast nor memory-efficient compared to current tools.
-     The authors have claimed that they have tested the algorithm on metatranscriptomic data and they have obtained decent assemblies. But no results have been shown in the manuscript as well as in the supplementary data.

Level of Interest

Please indicate how interesting you found the manuscript: Choose an item.

Quality of Written English

Please indicate the quality of language in the manuscript: Choose an item.

Declaration of Competing Interests

Please complete a declaration of competing interests, considering the following questions:

- Have you in the past five years received reimbursements, fees, funding, or salary from an organisation that may in any way gain or lose financially from the publication of this manuscript, either now or in the future?
- Do you hold any stocks or shares in an organisation that may in any way gain or lose financially from the publication of this manuscript, either now or in the future?
- Do you hold or are you currently applying for any patents relating to the content of the manuscript?
- Have you received reimbursements, fees, funding, or salary from an organization that holds or has applied for patents relating to the content of the manuscript?
- Do you have any other financial competing interests?
- Do you have any non-financial competing interests in relation to this paper?

If you can answer no to all of the above, write 'I declare that I have no competing interests' below. If your reply is yes to any, please give details below.

I declare that I have no competing interests

I agree to the open peer review policy of the journal. I understand that my name will be included on my report to the authors and, if the manuscript is accepted for publication, my named report including any attachments I upload will be posted on the website along with the authors' responses. I agree for my report to be made available under an Open Access Creative Commons CC-BY license (http://creativecommons.org/licenses/by/4.0/). I understand that any comments which I do not wish to be included in my named report can be included as confidential comments to the editors, which will not be published.

Choose an item.

To further support our reviewers, we have joined with Publons, where you can gain additional credit to further highlight your hard work (see: https://publons.com/journal/530/gigascience). On publication of this paper, your review will be automatically added to Publons, you can then choose whether or not to claim your Publons credit. I understand this statement.

Yes Choose an item.
