# Supplementary material for: rnaSPAdes: a de novo transcriptome assembler and its application to RNA-Seq data
Source: Gigascience. 2019 Sep 3;8(9):giz100. doi: 10.1093/gigascience/giz100 (PMC6736328; doi:10.1093/gigascience/giz100)
Supplement: giz100_Reviewer_3_Report_Revision_1 — Marcel Schulz -- 5/17/2019 Reviewed [file giz100_reviewer_3_report_revision_1.pdf]

Reviewer Report

Title: rnaSPAdes: a de novo transcriptome assembler and its application to RNA-Seq data

Version: Revision 1 **Date:** 5/17/2019

Reviewer name: Marcel Schulz

Reviewer Comments to Author:

In this work, Bushmanova et al have proposed an algorithm for transcriptome assembly inspired from the genome assembler SPAdes. Compared to the method previously proposed, the authors have made certain changes to their algorithm namely 1) Removal of the error correction step using BayesHammer error correction algorithm and 2) Addition of the step 'removal of isolated edges' in the graph processing step. The authors have also added two new high coverage datasets for analyzing the performance of their algorithm. I appreciate the response by the authors to my previous comments, but unfortunately they have not addressed the major caveat of the study.
1.My previous question:
- The authors have compared rnaSPAdes against various other transcriptome assembler and have shown that rnaSPAdes performs sometimes better (in some statistics). The kmer parameter is one of the most important parameter in an assembly procedure. The authors have optimized the kmer parameter for their own algorithm but have kept the default kmer parameter for other algorithms, which are completely different than the rnaSPAdes kmer often. Hence, the comparison is unfair as they are not made on similar grounds. Combined with the fact that there are no clear methodological improvements the results remain mostly inconclusive, except maybe the additional use of error cor-rection is helpful as reported before.
2. Their answer
We absolutely agree that k-mer size is one of the most important parameters for de novo sequence assembly, and this is exactly the reason why we decided to optimize it. However, we did not choose an optimal k value for each dataset separately, but developed a universal strategy that au-tomatically computes nearly optimal k for any kind of data depending on the read length. Thus, se-lecting an optimal value for each assembler on every dataset seems to be unfair, especially taking into account the fact that in real assembly projects the ground truth is unknown and choosing the best assembly becomes non-trivial.
The procedure of selecting optimal k-mer values can be also considered as methodological im-provement comparing to other tools (which have just a fixed k-mer size(s) for all cases) and a part of the developed pipeline. Based on our experience with the assembly software users we see that the majority of them use the default k-mer values and rarely change it.
Additionally, running all assemblers with different k-mer sizes on several datasets and assessing their quality would require roughly several processor years.
3. my new response
It is good that we agree on the importance of kmer values. However, it is not novel to suggest to use

more than one k-mer value for transcriptome de novo assembly. The trans-Abyss paper (cited, 2010), the Oases paper (cited, 2013) and more recent work (KREATION package, Informed kmer selection for de novo transcriptome assembly, Bioinformatics 2016) has clearly demonstrated that using more than one k-mer especially a smaller (more sensitive for lowly-expressed) and a higher one (to deal with excessive coverage and resolve repeats) clearly boosts the overall assembly performance. Thus, there is no novelty in their observation that using two kmers are better than using one. Even worse, trans-ABYSS for example allows to merge the results for two kmers, why was this not done, if the authors believe that using two kmers is much better than one? Why do they not use the other assemblers at these good k-mer values (if possible) ?
Concerning this point, I think the contribution of this work could, in the best case, be that they say that easy rules suffice for the selection of two kmers, for example in comparison to the more data-driven strategy of the KREATION approach. But this is not what they analyze with their method comparison. Instead they use a bunch of diverse assemblers each at their default kmer values, which are, of course, not ideal for all datasets. The only exception is IDBA-trans which runs over several kmer values by default.
Unless they change the parameters of the other assemblers and run the assemblers for which this is feasible with the same two kmers (trans-abyss, Bridger, IDBA-tran) it remains unclear whether their software in fact has an advantage.
Similarly, the first results part of the paper where they state that Spades performs better than other de novo transcriptome assemblers is unfair, and if they correct the use of kmers may look different (table 1).
Minor comment:
In all the tables authors have given names (column names) of the assemblers as IDBA, SOAP, ABySS and Bloom which are genome assemblers (Although they have named it correctly in the table legends). I would suggest to keep the naming consistent as in the current form it might create confusion for the readers.

Level of Interest

Please indicate how interesting you found the manuscript: Choose an item.

Quality of Written English

Please indicate the quality of language in the manuscript: Choose an item.

Declaration of Competing Interests

Please complete a declaration of competing interests, considering the following questions:

- Have you in the past five years received reimbursements, fees, funding, or salary from an organisation that may in any way gain or lose financially from the publication of this manuscript, either now or in the future?
- Do you hold any stocks or shares in an organisation that may in any way gain or lose financially from the publication of this manuscript, either now or in the future?
- Do you hold or are you currently applying for any patents relating to the content of the manuscript?
- Have you received reimbursements, fees, funding, or salary from an organization that holds or has applied for patents relating to the content of the manuscript?
- Do you have any other financial competing interests?
- Do you have any non-financial competing interests in relation to this paper?

If you can answer no to all of the above, write 'I declare that I have no competing interests' below. If your reply is yes to any, please give details below.

I declare I have no competing interests

I agree to the open peer review policy of the journal. I understand that my name will be included on my report to the authors and, if the manuscript is accepted for publication, my named report including any attachments I upload will be posted on the website along with the authors' responses. I agree for my report to be made available under an Open Access Creative Commons CC-BY license (http://creativecommons.org/licenses/by/4.0/). I understand that any comments which I do not wish to be included in my named report can be included as confidential comments to the editors, which will not be published.

Choose an item.

To further support our reviewers, we have joined with Publons, where you can gain additional credit to further highlight your hard work (see: https://publons.com/journal/530/gigascience). On publication of this paper, your review will be automatically added to Publons, you can then choose whether or not to claim your Publons credit. I understand this statement.

Yes Choose an item.
