# Supplementary material for: rnaSPAdes: a de novo transcriptome assembler and its application to RNA-Seq data
Source: Gigascience. 2019 Sep 3;8(9):giz100. doi: 10.1093/gigascience/giz100 (PMC6736328; doi:10.1093/gigascience/giz100)
Supplement: giz100_Reviewer_4_Report_Revision_1 — Christian Cole -- 12/20/2018 Reviewed [file giz100_reviewer_4_report_revision_1.pdf]

Reviewer Report

Title: rnaSPAdes: a de novo transcriptome assembler and its application to RNA-Seq data

Version: Original Submission **Date:** 12/20/2018

Reviewer name: Christian Cole

Reviewer Comments to Author:

The manuscript reports on the adaptation of the well-known SPAdes de novo genome assembler for use on transcriptome assembly which is called rnaSPAdes. It provides a thorough comparison with 7 other de novo transcriptome tools using three benchmarking metrics.

Major comments

Concerned that results are more to do with the samples chosen rather than the assemblers themselves. See poor Transrate scores.

It is great that the authors chose to re-use existing datasets for their testing, however, the choice of samples should have been better. The size of the datasets are the barely enough for an RNA-seq differential expression study and inadequate for de novo assembly. I am concerned that the low coverage (18-37%) seen in Tables 1 & 2 and the poor Transrate contig scores (mostly <0.25) in Table S1-S4 is dominated by the low numbers of reads for the size of the transcriptome in the mammalian species. Better assemblies are achieved with larger datasets (see [1] 55M reads for mouse & [2] 100-400M reads for lower eukaryote) and being at the margins may be the reason why there isn't any consistency in performance. For example, SPAdes is best (in terms of rnaQUAST) for the simulated mouse data, but rnaSPAdes is best for the real mouse data (Tables S2 & S4) and the reverse is true for the human data (Tables S1 & S3).

There is no information from the authors regarding how the raw data was quality checked and trimmed. It is common practice to verify the quality of the data with FastQC[3] and trim low quality reads/bases or adapter contamination with a trimming tool like Trimmomatic[4]. See Kerr et al[5] as an example. As per the comment above I'm concerned that the lack of QC has negatively impacted on the final assemblies[6].

Not withstanding the comments above SPAdes regularly outperforms rnaSPAdes (e.g. Figures 2 & 3 plus simulated mouse data and read human data). The authors do not mention that despite the aim of the study is to produce an improvement to SPAdes for de novo transcriptome assembly. Do they have any ideas why that may be?

De novo assembly attempts to recover the transcriptome present in a sample without prior knowledge. This has advantages over standard read mapping to transcripts or genomes in that novel transcripts or even genes can be

identified. The authors don't mention this nor highlight any numbers for novel transcripts identified.
Given the above regarding the low read depth of the original datasets, it would be of interest to see how much better transcript coverage is gained in performing a de novo transcriptome assembly over a straight-forward read mapping to the transcriptome with salmon[7] or kallisto[8].
Minor comments
In the abstract the authors state that there are "surprising computational parallels between assembly of transcriptomes and single-cell genomes" (p.1 line 33). I'm not sure it is particularly surprising nor do the authors expand on why it would be.
The authors should be acknowledged for the re-use of public and existing data, although it would be useful to know the tissue source of the data as it is relevant to the diversity and quantity of transcripts expected.
The use of the word "superiorities" is not correct (p.2 lines 30 & 49), better to use "strengths".
Figure 1 needs to be clearer. Which data set does it represent? The colours chosen are too similar to be able to differentiate well and for (a) the use of symbols and/or dashed lines would help enormously. (b) is probably not worth showing as the authors themselves admit the numbers are "insignificant" (p. 3 line 59). It would be better used for presenting a score that is more relevant such as misassemblies or duplication ratio.
Typo p3, column 2, line 60 "manages to maintains" should be "manages to maintain".
p4, column 1, lines 44-47 need to clarify how the BUSCO results "confirms .. the problem of high duplication levels".
Figure 2 needs to clarify whether the data are for the real or simulated mouse and human results.
In the discussion, very vague terms are used to describe the results. For example, "rather decent" (p5, col1, l25) or "moderate amount" (p5, col2, l9). The authors need to be more precise in their analysis by using numbers and refer to tables, otherwise it is essentially meaningless. Similarly, the 'soft', 'normal' and 'hard' filtering settings mentioned in Table S11 and p10, col1, l17-18 are too vague and need detailing.
In the conclusion, the terms 'sensitivity' and 'specificity' need to be clarified as nowhere in the results are they defined or quantified for any of the tools.
Typo p6, col2, l18 "alternations" should be "alterations".
As a non-expert on de Bruijn graphs I could not confidently review that part of the methods although it generally appears reasonable.
[1] https://doi.org/10.1038/nbt.1883
[2] https://doi.org/10.1186/s12864-017-3505-0

[3] https://www.bioinformatics.babraham.ac.uk/projects/fastqc/
[4] https://doi.org/10.1093/bioinformatics/btu170
[5] https://doi.org/10.1186/s12864-017-3577-x
[6] https://doi.org/10.3389/fgene.2014.00013
[7] https://doi.org/10.1038/nmeth.4197
[8] https://doi.org/10.1038/nbt.3519

Level of Interest

Please indicate how interesting you found the manuscript: Choose an item.

Quality of Written English

Please indicate the quality of language in the manuscript: Choose an item.

Declaration of Competing Interests

Please complete a declaration of competing interests, considering the following questions:

- Have you in the past five years received reimbursements, fees, funding, or salary from an organisation that may in any way gain or lose financially from the publication of this manuscript, either now or in the future?
- Do you hold any stocks or shares in an organisation that may in any way gain or lose financially from the publication of this manuscript, either now or in the future?
- Do you hold or are you currently applying for any patents relating to the content of the manuscript?
- Have you received reimbursements, fees, funding, or salary from an organization that holds or has applied for patents relating to the content of the manuscript?
- Do you have any other financial competing interests?
- Do you have any non-financial competing interests in relation to this paper?

If you can answer no to all of the above, write 'I declare that I have no competing interests' below. If your reply is yes to any, please give details below.

I declare that I have no competing interests.

I agree to the open peer review policy of the journal. I understand that my name will be included on my report to the authors and, if the manuscript is accepted for publication, my named report including any attachments I upload will be posted on the website along with the authors' responses. I agree for my report to be made available under an Open Access Creative Commons CC-BY license (http://creativecommons.org/licenses/by/4.0/). I understand that any comments which I do not wish to

be included in my named report can be included as confidential comments to the editors, which will not be published.

Choose an item.

To further support our reviewers, we have joined with Publons, where you can gain additional credit to further highlight your hard work (see: https://publons.com/journal/530/gigascience). On publication of this paper, your review will be automatically added to Publons, you can then choose whether or not to claim your Publons credit. I understand this statement.

Yes Choose an item.
